# Supplementary material for: Preconception care services in Northern Ethiopia: A qualitative exploration of awareness, experiences, challenges, opportunities, and prospects
Source: PLoS One. 2025 Dec 3;20(12):e0337548. doi: 10.1371/journal.pone.0337548 (PMC12674573; doi:10.1371/journal.pone.0337548)
Supplement: S1 File — (PDF) [file pone.0337548.s001.pdf]

Consolidated criteria for reporting qualitative studies (COREQ): 32-item checklist

(Source: Tong A, Sainsbury P, Craig J. Consolidated criteria for reporting qualitative research (COREQ): a 32-item checklist for interviews and focus groups. Int J Qual Health Care. 2007 Dec;19(6):349-57. doi: 10.1093/intqhc/mzm042. Epub 2007 Sep 14. PMID: 17872937.)

No Item	Whether an item is stated in the manuscript (Yes/No)
Domain 1: Research team and reflexivity	Yes
1. Interviewer/facilitator	Yes
2. Credentials	Yes
3. Occupation	Yes
4. Gender	Yes
5. Experience and training	Yes
6. Relationship established	Yes
7. Participant knowledge of the interviewer	Yes
8. Interviewer characteristics	Yes
Domain 2: study design	
9. Methodological orientation and Theory	Yes
10. Sampling	Yes
11. Method of approach	Yes
12. Sample size	Yes
13. Non-participation	Yes
14. Setting of data collection	Yes
15. Presence of non-participants	Yes
16. Description of sample	Yes
17. Interview guide	Yes
18. Repeat interviews	No
19. Audio/visual recording	Yes
20. Field notes	Yes
21. Duration	Yes
22. Data saturation	Yes
23. Transcripts returned	No
Domain 3: analysis and findings	
24. Number of data coders	Yes
25. Description of the coding tree	No
26. Derivation of themes	Yes
27. Software	Yes
28. Participant checking	No
29. Quotations presented	Yes
30. Data and findings consistent	Yes
31. Clarity of major themes	Yes
32. Clarity of minor themes	Yes
