# Supplementary material for: Preconception care services in Northern Ethiopia: A qualitative exploration of awareness, experiences, challenges, opportunities, and prospects
Source: PLoS One. 2025 Dec 3;20(12):e0337548. doi: 10.1371/journal.pone.0337548 (PMC12674573; doi:10.1371/journal.pone.0337548)
Supplement: Attachment — Submitted filename: PONE-D-24-41198_R1_reviewer_With comments.pdf [file pone.0337548.s006.pdf]

PLOS ONE

Awareness and uptake of preconception care services in Northern Ethiopia – a qualitative exploration of experiences, challenges, opportunities, and prospects

--Manuscript Draft--

**Manuscript Number:**	PONE-D-24-41198R1
**Article Type:**	Research Article
**Full Title:**	Awareness and uptake of preconception care services in Northern Ethiopia – a qualitative exploration of experiences, challenges, opportunities, and prospects
**Short Title:**	Awareness and uptake of preconception care services
**Corresponding Author:**	Gebremedhin Gebreegziabher Gebretsadik<br>Adigrat University<br>Mekelle, ETHIOPIA
**Keywords:**	Key words: Preconception Care Services, Experiences, Challenges, Opportunities, Explorative Qualitative Study, Tigray
**Abstract:**	Abstract<br>Introduction<br>Preconception care (PCC) has emerged as a key component of maternal continuum care worldwide, focusing on reducing poor pregnancy outcomes. Improving services requires addressing opportunities and challenges within the health system, but in Ethiopia it is often a neglected service . Hence, this study explores the experiences, challenges, and opportunities related to PCC services in Tigray, Northern Ethiopia.<br>Methods<br>We conducted an exploratory qualitative study involving 21 in-depth interviews with mothers who experienced adverse pregnancy outcomes and HCPs, who work in maternal, neonatal, and child health, and health extension workers. Additionally, we held six focus group discussions with women who had history of pregnancy. we also conducted key informant interviews with 10 maternal, newborn and child health experts from regional health bureau, district health offices, and professional associations. The study was conducted from January 26, 2024, to April 4, 2024 across four rural districts and two urban areas in Tigray, Northern Ethiopia. Discussions and interviews were audio-recorded, transcribed to the local language “Tigrigna”, then translated into English and thematically coded using ATLAS-ti v.7.5.4 software.<br>Results<br>Some women, particularly those belonging to high-risk groups, are aware of PCC services. A significant proportion of HCPs, especially gynecologists and physicians have some knowledge of PCC, recognize its importance, and provide specific components of PCC interventions. However, these services are often delivered in a fragmented manner, primarily targeting high-risk women. Identified challenges include traditional beliefs and misconceptions, insufficient counseling on contraceptive services, social influences, service costs, high workloads, perceived shortages of medicines and medical equipment, and the fragmented nature of service delivery. Conversely, opportunities include utilizing existing community platforms and an expressed desire for PCC services. Moreover, the use of diverse communication strategies, linking communities with health facilities, involving high-risk mothers as educational role models, and integrating package-based PCC services into the healthcare system were explored as perceived suggestions.<br>Conclusion<br>With the exception of high-risk women, most women have little to no knowledge about PCC services. Furthermore, although many HCPs possess some understanding of PCC, they deliver only a limited range of interventions, primarily catering to self-initiated high-risk mothers. Challenges identified include traditional beliefs and misconceptions, inadequate counseling on contraceptive services, social influences, high service costs, and fragmented service delivery. Existing community platforms and the perceived desire for PCC services were highlighted as opportunities to enhance PCC services. Strategies such as utilizing diverse communication methods, involving high-risk mothers as role models, strengthening community engagement activities, and improving linkages between communities and health facilities were proposed. Additionally, promoting home-based self-care was explored as a suggestion for

Powered by Editorial Manager® and ProduXion Manager® from Aries Systems Corporation

	improving PCC services. Integrating package-based PCC services into the healthcare system to routinely serve all eligible women of reproductive age was recommended to improve both awareness and uptake of PCC. Finally, tailored interventions were deemed essential for improving PCC awareness and utilization both at the community and facility levels.
**Order of Authors:**	Gebremedhin Gebreegziabher Gebretsadik
	Alemayehu Bayray Kahsay
	Andargachew Kassa
	Amanuel Gessessew
	Zohra Lassi
	Afework Mulugeta
**Response to Reviewers:**	we respond your comments and suggestion point by point. the detailed description was included in the respond to review document
**Additional Information:**	
**Question**	**Response**
**Financial Disclosure**<br><br>Enter a financial disclosure statement that describes the sources of funding for the work included in this submission. Review the submission guidelines for detailed requirements. View published research articles from *PLOS ONE* for specific examples.<br><br>This statement is required for submission and **will appear in the published article** if the submission is accepted. Please make sure it is accurate.<br><br>**Funded studies**<br>Enter a statement with the following details:<br>• Initials of the authors who received each award<br>• Grant numbers awarded to each author<br>• The full name of each funder<br>• URL of each funder website<br>• Did the sponsors or funders play any role in the study design, data collection and analysis, decision to publish, or preparation of the manuscript?<br><br>Did you receive funding for this work?	The author(s) received no specific funding for this work.
**Competing Interests**<br><br>Use the instructions below to enter a	The authors have declared that no competing interests exist.

Powered by Editorial Manager® and ProduXion Manager® from Aries Systems Corporation

competing interest statement for this submission. On behalf of all authors, disclose any competing interests that could be perceived to bias this work—acknowledging all financial support and any other relevant financial or non-financial competing interests.<br><br>This statement is required for submission and **will appear in the published article** if the submission is accepted. Please make sure it is accurate and that any funding sources listed in your Funding Information later in the submission form are also declared in your Financial Disclosure statement.<br><br>View published research articles from *PLOS ONE* for specific examples.<br><br>**NO authors have competing interests**<br><br>Enter: *The authors have declared that no competing interests exist.*<br><br>**Authors with competing interests**<br><br>Enter competing interest details beginning with this statement:<br><br>*I have read the journal's policy and the authors of this manuscript have the following competing interests: [insert competing interests here]*<br><br>* typeset	
**Ethics Statement**<br><br>Enter an ethics statement for this submission. This statement is required if the study involved:<br><br>• Human participants<br>• Human specimens or tissue<br>• Vertebrate animals or cephalopods<br>• Vertebrate embryos or tissues<br>• Field research	The Institutional Review Board of Mekelle University, College of Health Sciences (reference: MU-IRB2075/2023), granted ethical approval. The Tigray Health Bureau issued a support letter, and the relevant district health offices and villages granted permission. Before data collection, we attached a one-page consent form to the questionnaire, explaining participants' autonomy. We fully explained the study's objectives, risks, and benefits and obtained informed consent from all participants. We prioritized privacy and confidentiality and informed participants of their right to withdraw at any time. We also requested permission to record focus group discussions and interviews.

Powered by Editorial Manager® and ProduXion Manager® from Aries Systems Corporation

Write "N/A" if the submission does not require an ethics statement.

General guidance is provided below. Consult the submission guidelines for detailed instructions. **Make sure that all information entered here is included in the Methods section of the manuscript.**

Format for specific study types

Human Subject Research (involving human participants and/or tissue)

- Give the name of the institutional review board or ethics committee that approved the study
- Include the approval number and/or a statement indicating approval of this research
- Indicate the form of consent obtained (written/oral) or the reason that consent was not obtained (e.g. the data were analyzed anonymously)

Animal Research (involving vertebrate animals, embryos or tissues)

- Provide the name of the Institutional Animal Care and Use Committee (IACUC) or other relevant ethics board that reviewed the study protocol, and indicate whether they approved this research or granted a formal waiver of ethical approval
- Include an approval number if one was obtained
- If the study involved *non-human primates*, add *additional details* about animal welfare and steps taken to ameliorate suffering
- If anesthesia, euthanasia, or any kind of animal sacrifice is part of the study, include briefly which substances and/or methods were applied

Field Research

Include the following details if this study involves the collection of plant, animal, or other materials from a natural setting:

- Field permit number
- Name of the institution or relevant body that granted permission

Powered by Editorial Manager® and ProduXion Manager® from Aries Systems Corporation

**Data Availability**<br><br>Authors are required to make all data underlying the findings described fully available, without restriction, and from the time of publication. PLOS allows rare exceptions to address legal and ethical concerns. See the PLOS Data Policy and FAQ for detailed information.<br><br>A Data Availability Statement describing where the data can be found is required at submission. Your answers to this question constitute the Data Availability Statement and **will be published in the article**, if accepted.<br><br>**Important:** Stating 'data available on request from the author' is not sufficient. If your data are only available upon request, select 'No' for the first question and explain your exceptional situation in the text box.<br><br>Do the authors confirm that all data underlying the findings described in their manuscript are fully available without restriction?	Yes - all data are fully available without restriction
**Describe where the data may be found in full sentences. If you are copying our sample text, replace any instances of XXX with the appropriate details.**<br><br>• If the data are **held or will be held in a public repository**, include URLs, accession numbers or DOIs. If this information will only be available after acceptance, indicate this by ticking the box below. For example: *All XXX files are available from the XXX database (accession number(s) XXX, XXX.*).<br>• If the data are all contained **within the manuscript and/or Supporting Information files**, enter the following: *All relevant data are within the manuscript and its Supporting Information files.*<br>• If neither of these applies but you are able to provide **details of access elsewhere**, with or without limitations, please do so. For example:	All relevant data are within the manuscript and its Supporting Information files

Data cannot be shared publicly because of [XXX]. Data are available from the XXX Institutional Data Access / Ethics Committee (contact via XXX) for researchers who meet the criteria for access to confidential data.<br><br>*The data underlying the results presented in the study are available from (include the name of the third party and contact information or URL).*<br>• This text is appropriate if the data are owned by a third party and authors do not have permission to share the data.<br><br>* typeset	
Additional data availability information:	Tick here if the URLs/accession numbers/DOIs will be available only after acceptance of the manuscript for publication so that we can ensure their inclusion before publication.

Powered by Editorial Manager® and ProduXion Manager® from Aries Systems Corporation

Manuscript

Click here to access/download;Manuscript;Manuscript.docx

Awareness and uptake of preconception care services in Northern Ethiopia – a qualitative exploration of experiences, challenges, opportunities, and prospects

Gebremedhin Gebreegziabher Gebretsadik[1, 2*], Alemayehu Bayray Kahsay [2], Andargachew Kassa [4], Amanuel Gessessew [3], Zohra Lassi, [5, 6], Afework Mulugeta [2]

[1] College of Medicine and Health Sciences, Adigrat University, Tigray, Ethiopia

[2] School of Public Health, College of Health Sciences, Mekelle University, Tigray, Ethiopia

[3] School of Medicine, College of Health Sciences, Mekelle University, Tigray, Ethiopia

[4] School of Public Health, College of Medicine and Health Sciences, Hawassa University, Ethiopia

[5] School of Public Health, Faculty of Health and Medical Sciences, University of Adelaide, Australia

[6] Robinson Research Institute, University of Adelaide, Australia

***Corresponding author:**
Email**:** gebremedhingebretsad@gmail.com (GG)

Abstract

Introduction

Preconception care (PCC) has emerged as a key component of maternal continuum care worldwide, focusing on reducing poor pregnancy outcomes. Improving services requires addressing opportunities and challenges within the health system, but in Ethiopia it is often a neglected service . Hence, this study explores the experiences, challenges, and opportunities related to PCC services in Tigray, Northern Ethiopia.

Methods

We conducted an exploratory qualitative study involving 21 in-depth interviews with mothers who experienced adverse pregnancy outcomes and HCPs, who work in maternal, neonatal, and child health, and health extension workers. Additionally, we held six focus group discussions with women who had history of pregnancy. we also conducted key informant interviews with 10 maternal, newborn and child health experts from regional health bureau, district health offices, and professional associations. The study was conducted from January 26, 2024, to April 4, 2024 across four rural districts and two urban areas in Tigray, Northern Ethiopia. Discussions and interviews were audio-recorded, transcribed to the local language "Tigrigna", then translated into English and thematically coded using ATLAS-ti v.7.5.4 software.

Results

Some women, particularly those belonging to high-risk groups, are aware of PCC services. A significant proportion of HCPs, especially gynecologists and physicians have some knowledge of PCC, recognize its importance, and provide specific components of PCC interventions. However, these services are often delivered in a fragmented manner, primarily targeting high-risk women. Identified challenges include traditional beliefs and misconceptions, insufficient counseling on contraceptive services, social influences, service costs, high workloads, perceived shortages of medicines and medical equipment, and the fragmented nature of service delivery. Conversely, opportunities include utilizing existing community platforms and an expressed desire for PCC services. Moreover, the use of diverse communication strategies, linking communities with health facilities, involving high-risk mothers as educational role models, and integrating package-based PCC services into the healthcare system were explored as perceived suggestions.

Conclusion

With the exception of high-risk women, most women have little to no knowledge about PCC services. Furthermore, although many HCPs possess some understanding of PCC, they deliver only a limited range of interventions, primarily catering to self-initiated high-risk mothers. Challenges identified include traditional beliefs and misconceptions, inadequate counseling on contraceptive services, social influences, high service costs, and fragmented service delivery. Existing community platforms and the perceived desire for PCC services were highlighted as opportunities to enhance PCC services. Strategies such as utilizing diverse communication methods, involving high-risk mothers as role models, strengthening community engagement activities, and improving linkages between communities and health facilities were proposed. Additionally, promoting home-based self-care was explored as a suggestion for improving PCC services. Integrating package-based PCC services into the healthcare system to routinely serve all eligible women of reproductive age was recommended to improve both awareness and uptake of PCC. Finally, tailored interventions were deemed essential for improving PCC awareness and utilization both at the community and facility levels.

Key words: Preconception Care Services, Experiences, Challenges, Opportunities, Explorative Qualitative Study, Tigray

Introduction

Preconception care (PCC) has garnered significant attention globally, primarily due to its ability to reduce the risk of adverse pregnancy outcomes (APOs). The existing continuum of care lacks pre-pregnancy care, and PCC addresses this gap by mitigating parental risk factors before conception, thereby enhancing outcomes for both mothers and infants (1). To mitigate the burden

of APOs, both the World Health Organization (WHO) and the Centers for Disease Control and Prevention (CDC) universally recommend PCC as an essential part of maternal healthcare. They advise that women intending to become pregnant should receive at least one preconception care checkup (2). Following CDC/WHO recommendations, several low- and middle-income countries (LMICs) including Bangladesh, the Philippines, and Sri Lanka have innovatively integrated PCC into their health systems (2, 3). Besides, some Sub-Saharan Africa(SSA) countries, like South Africa(4), Kenya (5) and Ethiopia (6, 7), have also incorporated PCC into their healthcare systems and are working to leverage this initiative to achieve the Sustainable Development Goals (SDGs) in a timely manner.

Worldwide, approximately 295,000 maternal deaths occur each year due to complications from pregnancy or childbirth (8). Additionally, there are around 23 million miscarriages (9), 14.8 million live preterm births(10), and 295,000 neonatal deaths caused by congenital anomalies annually(11). The prevalence of congenital anomalies in LMICs is significantly high, with 94% of cases being severe(12). Similarly, the burden of APOs in LMICs remains a significant global issue. For example, the burden of APOs in SSA is 29.7% (13). Ethiopia is among the LMICs with high rates of maternal and neonatal deaths, at 412 per 100,000 live births and 33 per 1000 live births, respectively (14, 15). Before the war, Tigray's health facilities ranked among the best in Ethiopia for maternal, newborn, and child health services(16). In the Tigray region, neural tube defects occur at a rate of 131 per 10,000 births (17). However, since the conflict began in Since the conflict began in November 2020, significant damage to the health system has severely disrupted maternal care and essential services. This has exacerbated preconception risks, including unwanted pregnancies, unsafe abortions, home deliveries, malnutrition, and increased

maternal, newborn, infant, and child mortality rates (18-20). For instance, the maternal mortality rate during the conflict reached 840 deaths per 100,000 live births.(21).

Despite the WHO's recognition of its feasibility in LMICs, PCC has not been successfully adopted in Africa (2). HIV testing, family planning, and contraceptive services are commonly utilized components in PCC interventions, while supplementation of folic acid is less widely used (22). A recent review in SSA found that the uptake and knowledge of PCC were only 24.05% and 33.27% (23), respectively. A systematic review further revealed that in Ethiopia, only 30.95% of women had knowledge of PCC, with just 16.27% utilizing it (24), and the provision was low at 15% (25). Ethiopia has set ambitious targets under its reproductive health strategic plan to align with SDG 3.1, aiming to significantly reduce preventable pregnancy-related morbidity and mortality by 2025 (26). These targets include increasing the proportion of pregnant women receiving PCC to 25%, reducing the neonatal mortality rate from 33 to 21, and decreasing the maternal mortality rate to 271 per 100,000 live births (26). However, information is lacking on how the healthcare system has implemented PCC services.

Studies have indicated that identifying practical and feasible interventions based on local contexts is essential for raising awareness and improving PCC service adoption (27, 28). A qualitative study in high-income countries revealed that unfavorable attitudes, a lack of knowledge about PCC, limited resources such as time and guidelines, and unclear responsibilities for providing PCC are barriers to its provision (29). In Southwest Ethiopia, a study focused on barriers to the uptake of PCC identified some barriers, including poor knowledge, unplanned pregnancies, heavy workloads, service costs, distance, unavailability of services, and insufficient attention from media personnel (22).

Although Ethiopia has strategically integrated PCC into its health system with newly developed guidelines (6, 7), there is limited evidence, particularly on the challenges and opportunities within the system that influence service enhancement. This study addresses this gap by examining experiences, challenges, and opportunities from the perspectives of experts, HCPs, and women with pregnancy histories. It evaluates current practices and provides recommendations to improve PCC awareness and uptake, guiding the development and implementation of effective interventions.

Materials and methods

Study design: We employed an exploratory qualitative study design to investigate the experience of the current practices, challenges and opportunities for PCC services. The study was reported in accordance with the Consolidated Criteria for Reporting Qualitative Research (COREQ) checklist (30).

Study setting

We conducted the study in the eastern and central regions of Tigray, Northern Ethiopia, with populations of 994,346 and 1,522,596, respectively. According to the Tigray Regional Health Bureau, in 2020, the region had a total of 14,423,731 healthcare providers (HCPs), including 3,074 health extension workers (HEWs). These zones include 591,481 women of reproductive age (23.5% of the population). The study focused on specific districts and urban areas in both zones, from January 26, 2024, to April 4, 2024. We specifically targeted two rural districts and one urban district in the eastern zone, along with two rural districts and one urban woreda in the central zone of Tigray. The region invested in primary health care units, achieving a 91.7% coverage rate. However, the war damaged over 80% of health facilities, leading to a 40% decline in maternal and child health services, including institutional deliveries (31).

Participants

In the focus group discussions (FGDs), we included women who have had a history of pregnancy or are currently pregnant and who have the intention to be pregnant. We recruited high-risk mothers (history of APOs like stillbirth, neonatal death, congenital anomalies, perinatal death, miscarriage, post-partum hemmoragge, recurrent abortion (32) or history of chronic medical disease like diabetic mellitus , hypertension or HIV) for the in-depth interviews (IDIs). Using purposive sampling, we identified mothers who communicated with HEWs and women development groups (WDGs) from HEW registers, considering their pregnancy and risks. Furthermore, healthcare providers HCPs, including midwives, nurses, health officers, and medical doctors stationed in Maternal, Newborn, and Child Health (MNCH) units, were identified with the guidance of the medical director or MNCH coordinators of these health facilities for IDIs. Additionally, experts from the District Health Office of MNCH or Health Extension Program (HEP) case team in selected districts, the Reproductive maternal neonatal child health (RMNCH) case team from the regional health bureau, and professionals from the Ethiopian Midwives Association (EMA) and Ethiopian Obstetrics and Gynecology Association (ESOGA) participated in the Key informant interviews (KIIs). Furthermore, we selected HCPs for both IDIs and KIIs based on their substantial experience in MNCH and their ability to communicate effectively, thereby providing valuable insights into the current state of PCC.

Data collection

We obtained participant information through IDIs, KIIs, and FGDs. To triangulate and validate the data, we included mothers, medical professionals working in MNCH units, MCNH experts from district and regional health offices, and associations.

Mothers for FGDs and IDIs were identified with the assistance of HEWs and WDGs within the community at their households. After obtaining their consent, we interviewed in private settings, such as village health posts or households, to ensure privacy and minimize background noise. Medical directors supported selecting HCPs for IDIs, who were then interviewed in private rooms at their workplaces. Similarly, we conducted face-to-face interviews with MCH experts for KIIs in private rooms at their workplaces. The number of participants was determined based on data saturation, which occurs when participants' descriptions become repetitive. Sampling continued until no new information emerged, indicating saturation was reached (33).

Six FGDs were conducted, one in each district, involving 7-9 mothers to become pregnant, each session lasting 56-96 minutes. Eight mothers with a history of APOs participated in IDIs, averaging 42 minutes per interview. Additionally, 13 HCPs from six health centers and health posts were interviewed, averaging 46 minutes each. KIIs involved 10 MNCH experts, seven from district health offices and RHB, and three clinicians and academics from ESOGA & EMA, with 38-70 minutes of interviews.

The primary investigator (GG) and three other PhD students in public health (GB, KK, and FT) who are experienced and trained in qualitative research conducted the interviews and FGDs. The four interviewers were paired into two groups: one note-taker and one interviewer. We used interviews and discussion guides to explore experiences, challenges, and opportunities in providing PCC services. We developed semi-structured guides for four groups: mothers to be pregnant, high-risk mothers, HCPs working in the MNCH unit, MNCH experts, clinicians and academics in MNCH. These guides were initially drafted in English, translated into the local language, Tigrigna. FGDs were organized in circular seating arrangements to facilitate interactive discussions among participants (34). The interview guides comprised open-ended

questions [S1]. Furthermore, the interview guide incorporated probing questions. Interviewers actively guided respondents through the questions outlined in the guide, using probing techniques to prompt further explanation of participants' responses. Throughout the interviews, they audio-recorded all sessions, took field notes to document key points, and observed participants' non-verbal cues in-depth.

Trustworthiness

We ensured the reliability of our findings by implementing rigorous quality measures. The data collectors were trained on tools, interview techniques, participant selection, concept of reflexivity, and consent procedures. We pretested the topic guide in a similar setting before starting data collection with revisions made based on feedback. During data collection, the team held daily debriefing sessions to address emerging issues and spent extended time with participants to gain deeper insights. We extended the research period to gain an in-depth understanding of the phenomena. We shared participant transcripts for verification and incorporated their feedback. We conducted data collection and analysis simultaneously, triangulating findings from interview transcripts with field notes. Experienced researchers fluent in the local language and culture translated, transcribed, and coded parts of the audio recordings while the primary investigator reviewed their work to ensure accuracy. Preliminary results were presented to experts and peers, leading to further refinement of the guides. While conducting participant interviews, creating codes, and organizing these codes into categories and themes, the researcher team bracketed their prior experiences and knowledge to enhance the quality of the results. To ensure the consistency of our findings, we conducted member checking with four participants, each representing a distinct group: HCPs, MNCH experts, and the two groups of

mothers. Additionally, we employed IDIs, KIIs, and FGDs as data collection methods to inform and guide subsequent discussions.

Data analysis

The FGDs, IDIs and KII were audio recorded, transcribed verbatim in the local language "Tigrigna", and translated into English. Verbatim transcripts of the data saved as an independent MS Word file were imported, stored, managed, and coded using Atlas.ti v.7.5.4 (Scientific Software Development GmbH, Berlin,Germany) qualitative data analysis software. The first author (GG) conducted the initial coding by thoroughly listening to the audio recordings from the FGDs and interviews and carefully reviewing all the transcripts to become familiar with the content and to identify initial coding. In addition, we linked field notes and investigator memos to the respective files in the software to assist in the analysis.

The first author (GG) conducted line-by-line coding. We used both process and values coding to analyze all the transcripts. Additionally, we applied a hybrid approach, inductive and deductive coding methods during the analysis (35). Another investigator (AM) coded the five interviews and the FGDs to check inter-coder reliability. Transcription, translation, analysis, and data collection were conducted simultaneously throughout the data collection period. The investigators grouped similar codes to create categories and subcategories. We reviewed and revised the codes for clarity and consistency then consolidated them to eliminate redundancy and overlap in the analysis. We used a thematic analysis approach to identify the major themes across the categories and subcategories (36). Furthermore, during the write-up, we performed content analysis to describe the frequency of participants in subcategories. Quotes that best described the various categories and frequently expressed sentiments across several groups were chosen and presented in italics.

Ethics approval

The Institutional Review Board of Mekelle University, College of Health Sciences (reference: MU-IRB2075/2023) granted ethical approval. The Tigray Health Bureau issued a support letter, and the respective district health offices and villages granted permission. We fully explained the study's objectives, risks, and benefits and obtained informed consent from all participants and the consent was approved by the college's institutional review board. We prioritized privacy and confidentiality and informed participants about their right to withdraw at any time. We also requested permission to record focus group discussions and interviews.

Results

Socio-demographic characteristics of the respondents

The qualitative study involved 79 participants, including 21 individuals in IDIs with HCPs and high-risk mothers. Additionally, 10 MNCH experts from district health offices, regional health bureaus, and professional associations participated in KIIs. Among the high-risk mothers, 63% were aged between 18 and 34 years, all were housewives, and 12.5 % were grand multiparous. Of the healthcare professional participants, 64% were female, with approximately 74% being midwives, public health professionals, or HEWs (Table 1). Additionally, 48 mothers to become pregnant in the future participated in the FGDs. Among these FGD participants, 63% were between 18 and 34 years old, and 46% had an educational level of high school or above (Table 2).

Table 1: Characteristics of the study participants in Tigray, Northern Ethiopia, 2024

Characteristics	IDIs(N=21)	KIIs(N=10)

		High risk mothers *(n=8)	HCPs* (n=13)	HCP experts*
Sex	Male	NA*	3	5
	Female		10	5
Age of participants, years	18-34	5	8	1
	35–49	3	5	7
	=>50	0	0	2
Work experience, years	<5 years	NA	2	1
	5-10 years		6	5
	11-20years		5	4
Occupation	House wife	8	NA*	NA*
Educational level	Not attended school	1	NA*	NA*
	Primary (1-8th	3		
	Secondary(9-12th)	4		
	Diploma	0	5	0
	BSc. Degree/MD*	0	8	5
	MSc. Master /specialty	0	0	5
Profession	Midwifery	NA*	5	2
	Public Health		1	5
	Family health		0	2
	Nursing		2	0
	MD/specialty		1	1
	Health Extension Workers		4	0
Current working unit	Clinician & academic	NA*	0	3
	MNCH expert		0	7
	MNCH unit		9	0
	HEP*		4	0

Number of births	0-1	2	NA*	NA*
	2-4	5		
	=>5	1		

NB: PCC: Preconception Care, HEP: Health extension program , MD: Medical Doctor, MNCH: Maternal, Neonatal, and Child Health, HCPs: Healthcare Providers working in Maternal, Neonatal, and Child Health related units, NA: Not Applicable: HCP Experts: Healthcare professionals working at woreda health offices, regional health bureaus, and academic institutions: High-risk mother: a mother with a history of adverse pregnancy outcomes or chronic medical conditions

Table 2: Characteristics of FGD participants in Tigray, Northern Ethiopia, 2024

Characteristics		(FGD* 1-6)=48 mothers
Age of participants, years	18-34	30
	35–49	18
Occupation	House wife	41
	Student	2
	Merchant	4
	Daily laborer	1
Educational level	Not attended school	3
	Primary (1-8th)	23
	Secondary(9-12th)	13
	Diploma	8
	Degree	1

Number of births	0-1	14
	2-4	30
	=>5	4

NB: FGDs* , Focussed Group Discussion

Themes: Through an analysis of the transcribed interviews, five major themes, encompassing nineteen sub-themes, emerged. Awareness, experience, challenges, opportunities, and suggestions themes have emerged from the qualitative data on PCC services (Table 3).

Table 3. A list of themes and sub-themes emerged from the data, Tigray, Northern Ethiopia,2024

Major themes	Sub-themes
Awareness of PCC services	Information
	Perceived benefits
Experiences of PCC services	Practices of PCC interventions
	Home-based preparation
Challenges of PCC services	Fragmented services
	Traditional beliefs and misconceptions
	Poor quality of counseling on contraceptive services
	Social influences
	High Workload
	Services cost
	Perceived lack of medicines and medical equipment
Opportunities of PCC services	Existing community platforms
	Perceived desire of PCC services

Perceived suggestions of for PCC services	Home-based initiative
	Use a variety of communication strategies
	Use high-risk mothers as role models
	Community engagement activities
	Community -health facility linkage
	Services integration

The conceptual framework, emerged from the qualitative data, illustrates the overall relationships among the factors involved in PCC service. A higher level of awareness and positive experiences positively infulences PCC services. Conversely, challenges such as traditional beliefs, misconceptions, and fragmented PCC services negatively affect PCC services, including the level of awareness and experiences. This framework) provides a guiding structure for implementing PCC services within the healthcare system (Fig. 1)

Figure 1: Framework of Experiences, Challenges, and Opportunities in PCC Services in Tigray, Ethiopia . Note: The dashed lines represent the negative influence on PCC services, while the solid lines indicate a positive influence.

1. Awareness of PCC services

Information of PCC: Access to information about a service is essential for utilizing it effectively. Without awareness of its existence, people cannot benefit from it. For many women and community members, the concept of PCC remains unfamiliar, as it is a relatively new approach that the healthcare system has not actively promoted. Both mothers and HCPs indicated that most women lack information about PCC, turning to healthcare services only after

pregnancy confirmation or fertility difficulties. This gap in understanding repeatedly results in confusion between PCC and prenatal care. High-risk mothers stated:

....."I do not know what pre-pregnancy care is?" ***(32 years old mother, 5th grade, IDI)***

*."Endie****....****the meaning of this local language is* ***(****I don't know****).*** *I am just keeping silent"* ***(28 years old mother, 8th grade, IDI)***

Similarly, a Clinical midwifery professional said,

".....there is a lack of information regarding preconception in the community; I couldn't expect mothers to visit and utilize preconception care. I believe that we healthcare providers and health professional associations didn't invest in it, and from my observation in our context, I can conclude that no mothers are coming to preconception care, even though it is not supported by research" ***(Midwifery professional, male, KII)***

On the Other hand, participants noted that some high-risk mothers have information about the services. They emphasized that HCPs should advise these mothers, including those with a history of diabetes, hypertension, HIV, spontaneous abortion, infertility, or congenital abnormalities, to undergo various health screenings before conception. These screenings should include tests for non-communicable and communicable diseases, sexually transmitted infections, mental health issues, gender-based violence, and other chronic conditions. Additionally, they should receive advice on proper nutrition and avoid harmful substances like alcohol. A high-risk mother said,

I have heard information on PCC from media outlets such as Tigrai Television, in a program called "Maeda Hakaym." I think it is good and beneficial to have a planned life and children. It sounds nice to have plan, preparations, and examinations and give birth based on planned decisions. Screening on gender-based violence, chronic disease, sexually transmitted infections,

substance use, mental health, nutritional counseling and balanced diet and consultation to a doctor in the presence of these factors and medication use is essential to prevent adverse pregnancy outcome" ***(35 years old mother, 10 th grade, IDI*).

Even some mothers explain the concept of PCC and understand its benefits.

"I understand that PCC means I need to check my health status, should not take alcoholic drinks, need to have mental stability, and limit the number of children that I have according to my income or relation to livelihood. These come to my mind when I am thinking about PCC" ***(29 years old mother, 10th grade, IDI)***

Besides, most HCPs have some knowledge of PCC and emphasize the importance of women's health before conception. Gynecology professionals, medical doctors, and, to a lesser extent, midwifery professionals have a better understanding of PCC concepts and interventions. Key PCC services identified by HCPs include HIV testing, folic acid supplementation, nutritional education, family planning, chronic disease screening, substance use advice, STI/HIV screening, medication management, and assessment of APOs such as Rh incompatibility, Td vaccination, abortion, and congenital anomalies. Among these, folic acid supplementation, nutritional advice, and family planning were most frequently mentioned.

HIV testing, folic acid supplementations, feeding practices, and nutrition are the things I know about preconception care services ***(Midwifery professional, female, IDI)***

All participants concurred that nearly all HCPs are knowledgeable about recommending three months of folic acid supplementation to mothers with a history of congenital anomalies and repeated abortions before conception.

Mothers who had a history of repeated abortion before conception are eligible to take folic acid

for three months. We don't give folic acid supplementation to all eligible women ***(HEW, female, IDI)***

On the other hand, participants reported that some HCPs have no information about PCC and do not even recognize its name.

.... No, I don't know. I don't even think there is such care here. It has been a long time since I started working here; I never heard anyone talking about it, and I never saw such service being provided here (***Nursing professional, female, IDI)***

We don't even know about preconception care ourselves. We health professionals don't know it (***HEW, female, IDI)***

Perceived benefit of PCC services

Mothers and HCPs emphasized that PCC offers valuable opportunities to assess women's health, enabling early identification and management of potential issues. This approach not only enhances maternal healthcare services but also helps avoid unnecessary expenses and reduces the risk of mother-to-child HIV transmission.

Give opportunities for screening of health status: PCC provides opportunities to assess the health status of mothers, enabling early detection of various conditions and diseases and identifying potential preconception risks that may lead to adverse pregnancy outcomes, as noted by participants. This aspect contributes to the promotion of women's health.

"Preconception care plays a crucial role in screening for various health risks such as substance use, gender-based violence, mental illness, HIV/STIs, and chronic conditions like diabetes, hypertension, and heart disease before conception occurs" ***(29 years old mother, 10th grade, IDI)***

Enhance maternal healthcare services: Some participants noted that PCC services would facilitate antenatal, delivery, and postnatal care services.

……."Moreover, providing expectant mothers with guidance on the timing of childbirth, pre-pregnancy preparations, pregnancy care, and postnatal support can significantly ease their journey. As emphasized earlier" ***(Public health professional, male, KII)***

"If we work on preconception care earlier now, they will be able to start ANC contact on their day" ***(Public health professional, female, KII)***

Prevent HIV transmission from mother to child: The optimal time to prevent HIV transmission from mother to child is during the pre-pregnancy period, as identified by participants.

……"It is essential to undergo various tests, such as HIV testing, before pregnancy to prevent the transmission of the virus to the unborn child" ***(24 years old mother, 10th grade, FGD)***

It (PCC) can help to prevent mother-to-child transmission of diseases ***(30 years old mother, diploma, FGD)***

Save unnecessary expenses and efforts: Participants pointed out that some women spend their money and energy on ultrasounds and other laboratory tests privately during pregnancy because they did not consult HCPs before becoming pregnant. However, if these tests were initiated earlier, mothers could avoid unnecessary expenses and efforts.

2. Experience of PCC services

Current Practices in the Components of PCC Interventions

HCPs highlighted a significant gap in healthcare settings, attributing it to the absence of routine PCC services with designated providers or specialized units, leading to missed opportunities for

comprehensive care. They observed that PCC interventions, such as folic acid supplementation and counseling, are delivered in a fragmented manner across various units, including antenatal care, gynecology/obstetrics, youth-friendly services, family planning, chronic disease clinics, and post-abortion care, particularly for high-risk mothers. Although midwives and gynecologists provide some services, participants emphasized that private clinics offer them more consistently.

A public health professional discussed how various units provide components of PCC interventions: *"It is done irregularly in youth-friendly services unit like the family planning itself, reproductive and diet education. But it is not deep. But something is tried. Usually, as I tell you, the peer-to-peer takes the biggest share"* ***(Public health professional, male, KII)***

.....in addition to these, mothers diagnosed with DM, threatened abortion, spontaneous abortion, recurrent stillbirth etc, are informed and appointed to consult a healthcare provider before getting pregnancy in gyn/obs OPD. The consultation is specifically regarding folic acid; there is no more service provided for other possible reasons ***(Midwifery professional, male, KII)***

In general, the majority of participants noted that the components of PCC interventions provided mainly for high-risk women are:

Contraceptive counseling for pregnancy delay: All participants, including both users and providers, noted that contraceptive counseling for delaying pregnancy was offered to all women of reproductive age within the community and healthcare system.

Provide folic acid supplementation: All participants, both users, and providers, agreed that folic acid supplementation was commonly provided to mothers with a history of chronic diseases such as diabetes, congenital anomalies, and spontaneous abortion, making it one of the most frequently practiced components of PCC interventions.

……"We provide folic acid for women who have a history of abnormal pregnancy like spinal bifida, hydrocephalus, and anencephaly. We give priority to these women" ***(MD, male, KII)***

Counseling about medication safety: Participants mentioned that some women with a history of chronic diseases such as diabetes and hypertension received counseling about medication safety before conception when they visited the health facility for follow-up.

" *Need advice about medication with alcohol, if mothers have diabetic mellitus, HIV, and Hypertension should advise her that the medicines should be changed first before pregnant for example ACE inhibitors drugs have teratogenic effect*" ***(MD, male, IDI)***

Counseling about substance use: Some mothers with a history of APOs, including abortion, chronic disease, congenital anomalies, and alcohol intake, received suboptimal counseling. They were advised to avoid medications that cause teratogenic effects and to abstain from substance use. Additionally, they were recommended to take folic acid supplements and undergo HIV testing before conception.

Women are counsel to take folic acid before 3 months and to avoid bad habits such as alcohol, smoking, and other addictive substances ***(Family health professional, female, KII).***

Contrarily, an MCH expert employed at the district health office provided insight into the availability of PCC services within healthcare facilities as follows:

In both urban and rural locations, pre-pregnancy care is currently nonexistent. It hasn't existed before, as far as I can tell. Policy, too, begins with family planning. Additionally, access to preconception care is not as high as indicated. I won't be questioned about whether or not I offer the services. I'm speaking to you based on my level of expertise. It was lately that I attended training. I received thorough training on PCC in Ethiopia , ***XXX town*** *and it isn't offered locally*

(Public health professional, male, KII)

Moreover, most of the mothers agreed that women seek pre-pregnancy care only when concerned about their health. A high-risk mother noted:

As a result of my personal health issues, I sought advice from healthcare providers. I was diagnosed with hypertension, which unfortunately led to a stillbirth in the past. Therefore, I consulted with health workers to ensure the best possible outcome when I planned my next pregnancy. I even visited referral hospitals for further guidance and was advised to visit healthcare facilities ahead of any future pregnancy. I followed this advice accordingly ***(35 years old mother, 9th grade, FGD****)*

Additionally, mothers reported not receiving any counseling as part of pre-pregnancy care, even those who visited health facilities to have their contraceptives removed in preparation for pregnancy.

"When you go to health facilities for contraceptive removal, the care providers will ask you "Is it because you want to conceive? " Then, they would simply remove you and do nothing else, even if you said yes. There is no preconception care, there is no counseling, and they didn't assess your eligibility for pregnancy. Due to this, the community doesn't have the awareness, doesn't know what PCC is or what to do, and as a result, there is no demand for the service."

(35 years old mother, 10th grade, IDI)

Home-based preparation: Adopting a pregnancy plan enables women to prepare effectively for conception, reducing modifiable risks and promoting healthy pregnancies and positive birth outcomes. While most mothers lack access to PCC services, some, especially high-risk mothers,

take proactive steps at home, such as saving for medications, modifying alcohol intake, improving their diet, and focusing on mental readiness.

"Before the mother becomes pregnant, she need to get prepared psychologically and economically. Psychologically prepared means mentally she needs to be stable, she needs to eat more than usual (4-5 times per day)" ***(29 years old mother, 10th grade, IDI)***

A mother mentioned that when planning for pregnancy, she prepares the chicken with local alcohol (siwa) for her husband and drinks " siwa tsiray " herself, believing it benefits the child.

*……."a chicken should be slaughtered and Siwa (local alcohol) should be prepared, it is done before you get pregnant or when you are thinking about pregnancy. It is mostly for my husband but myself also drinks the "*siwa tsiray" *and it is good for the child"* ***(35 years old mother, not attended school, FGD)***

On the contrary, participants emphasized that most mothers, particularly those without known health issues, do not take any action or invest in pregnancy preparation. A high risk mother stated:

……"I didn't plan to get pregnant. In my situation, I did not take any extra precautions before being pregnant, so I continued to eat normally and did not seek medical advice or counsel on PCC until after I became pregnant. ***(29 years old mother, 10th grade, IDI)***

3. Challenges of PCC services

Fragmented-based services: Participants observed that HCPS deliver fragmented PCC components to high-risk women, resulting in low awareness and uptake. Key challenges include the lack of PCC guidelines, limited government focus, and inadequate training.

"In general, the health system gave no focus to PCC. In practice, no structures have been established in healthcare institutions to support PCC. I think this is the main challenge. Hence, if

there is no center for PCC in the health system, if there is no focus on PCC, and if the health care provider does not emphasize PCC, the community will do so. I believe this is the primary challenge" ***(MD, male, KII)***

In general, all participants noted that while limited orientation or information about PCC was provided, the government prioritized and delivered package-based services such as antenatal care (ANC) and delivery services.

"A kind of orientation or information was given about PCC by partners, but they still do not get that much focus from the government or partners on PCC like the other health care services. Through education, the community shows behavioral change about child and maternal nutrition" ***(Family health professional, female, KII)***

Traditional beliefs and Misconceptions

Lack of felt need to disclose desire to conception: Mothers and HCPs have noted that, with few exceptions, most women tend to keep their desire to have a child confidential. Many women even conceal their pregnancies during the early months. They typically do not discuss their intentions to conceive with others unless it is with their husband, a very close friend. While discussing pre-pregnancy care may seem taboo or embarrassing within specific segments of the community, it might be disclosed after pregnancy is confirmed because pregnancy is perceived as a gift from St. Mary or God.

A high-risk mother said:

………" Discussing care before pregnancy with a neighbor or a part of the community is often considered impolite or embarrassing. In stead, it's more common to share this information once pregnancy is officially confirmed, as there's a belief that pregnancy is a blessing from St. Mary or God ***(32 years old mother, 5th grade, IDI****)*

"Mostly, it is in the latter stages of pregnancy that the woman discloses her pregnancy status; except for a for a few, most of the women keep it confidential" ***(29 years old mother, 10th grade, IDI)***

Additionally, an MCH expert said: *"Disclosing or consulting with healthcare providers before getting pregnant is the biggest challenge in the community. Mothers may feel ashamed to consult; normally, it shouldn't be "****(Pubich health professional, female, KII)***

Misconception about fear of side effects: Participants noted that contraception, especially the Depo injection, is believed to cause infertility. Women avoid using Depo because it delays pregnancy after discontinuation. Due to this misconception, many women are discouraged or restricted from using contraceptives.

"Now, for example, faith in the contraceptive (depo) causes a delay of pregnancy and hence many mothers do not use contraception" ***(MD, male, IDI)***

A maternal health expert pointed out that mothers discontinue contraceptives because they believe these may be responsible for the increase in congenital anomalies.

*……" You see, especially now, after encountering many congenital anomalies, numerous mothers have approached us to discontinue family planning"****(Midwifery professional, female, IDI)***

Misconception about intake of alcohol: Participants noted that consuming traditional alcohol ("Siwa" and "Myes") is not harmful except when taken with medication, particularly during the first four months of pregnancy. Additionally, some mothers believe that alcohol does not increase the risk of abortion or stillbirth; instead, they attribute these risks to poor nutrition, lack of adequate rest, and stress. Participants also mentioned that some elders believe consuming honey before pregnancy may facilitate the mental development and good health of the fetus later

during pregnancy.

"There is no problem with the intake of alcohol before pregnancy. Even though it does not cause any harm until four months of pregnancy, according to the lesson we took, Drinking alcohol is not allowed when you are taking medication. However, it has no problem with other issues. Drinking milk is not allowed after six months of pregnancy" ***(35 years old mother, not attended school, IDI)***

"Yes, for instance, our elders say it is good to take honey before pregnancy. They say it facilitates mental development and the good health of the foetus later during pregnancy. Also recommended by the community is "Myes" intake. I don't think these substances can cause abortion or stillbirth. Abortion and stillbirth are mainly caused by the factors I mentioned earlier, such as poor nutrition, a lack of adequate rest, and stress" ***(35 years old mother, 10th grade, IDI.***

Poor quality of counseling on contraceptive services

Mothers and HCPs agreed that inadequate counseling often deters women from using contraceptives, as they worry about side effects like delayed conception and bleeding. When women discontinue contraceptives, switch to alternatives, or stop using them altogether, unintended pregnancies can occur, disrupting the effective delivery of PCC services.

"I was on birth control for four years but stopped because I experienced intense bleeding or a hemorrhagic condition, which led to my pregnancy. It wasn't something I had planned for. I only used contraceptives for the first pregnancy (since it was my first, I didn't use them again), but all my other pregnancies were unplanned. They happened after I stopped using birth control due to negative side effects. I attempted to use birth control again but stopped due to these side effects and tried the calendar method, but that didn't work, and I ended up pregnant again. I didn't have

a specific plan for my reproductive life. A lot of it just happened by chance, not by design." ***(35 years old mother, 10th grade, IDI)***

"The healthcare provider is also sending mothers for each contraceptive without properly explaining what is wrong with it and what kind of mother it should be given to" **(MD, male, IDI)**

Social influences

Participants highlighted the influential role of husbands, older in-laws, and community leaders in encouraging women to plan pregnancies and seek healthcare advice. However, cultural beliefs often dictate that once a woman is pregnant, she must continue the pregnancy, regardless of her mental readiness. In some areas, women fear abandonment if they leave their husbands during pregnancy, while cultural norms discourage open discussions about family planning with in-laws or friends, causing embarrassment. Additionally, some elders advocate seeking blessings from holy water instead of consulting healthcare professionals.

"What is the barrier to pre-pregnancy testing that you explained is the backward culture. Even though she knows it, she wants this, she wants to have a baby because she thinks it will hurt, or she is ashamed, it's a shake to be told this is what she wants, but they know it. It is very embarrassingn't very comfortable to say that you want to have children with your mother-in-law or father-in-law, even with your friends. For example, I can tell them that I want to have a baby here, and my baby is grown. I may even have a desire inside, but they can go out and accuse me of being shameful" ***(35 years old mother, 10th grade, FGD)***

Some would believe in the service, and some would not, especially the old-times people (elders). *The elders may downplay the service like: "we are here and still managed to have kids despite there being no such service in our time," and ask, "Consulting who else were our grandmothers conceiving that you are doing so now?" and the like* **(35 years old mother, 10th grade, IDI)**

Mothers and HCPs observed that while some husbands with higher literacy levels recognize the importance of PCC, the majority perceive it as a low-priority issue. As a result, they do not support women seeking HCPs services before pregnancy.

The majority of them (husbands) are obstacles except a few ***(35 years old mother, not attended school, IDI)***

On the other hand, some participants highlighted that older mothers emphasize the importance of pre-pregnancy care, particularly for women facing infertility.

"They are very supportive of it. They are especially encouraged if you give them recognition regarding mothers and children. It's okay because it's normal in the normal. However, women with the problem of being unable to get pregnant are encouraged to consult healthcare providers by the mothers because they want you to give birth to them. They even tell you to go in hiding" ***(Public health professional, male, KII)***

High workload

HCPs noted that providing PCC services increases their workload and places additional burdens on staff, potentially leading to demands for extra benefits and staffing. When women come for child vaccinations and family planning but also request PCC, it requires additional time and commitment from HCPs. Providers highlighted that due to time constraints, some women were not screened.

"Implementing this program will increase our workload and place additional burdens on the staff, potentially leading to demands for extra benefits. This could pose a challenge if there is no allocated budget" ***(Midwifery professional, female, IDI)***

On the other hand, one HCP noted that if we work on PCC, it will make the provider's job easier because it decreases the risk factors like malnutrition, abortion, and other complications during

pregnancy and childbirth.

….”For sure, but if it is done here, I think it will make our job easier for us. So we have worked on preconception care and are fixing this malnutrition. His silence, which we call abortion, is being fixed while it is there. Stunting and underweight are being fixed there. So the professionals finish their work there” ***(Public health professional, male, KII)***

Services cost

Low socioeconomic status, service costs, and transportation expenses pose major barriers to accessing PCC services. While maternal health services like antenatal care, labor, delivery, and postnatal care are free in Ethiopia, participants highlighted that the costs of PCC, such as lab tests and imaging, are challenging for low-income mothers. This financial burden can result in missed opportunities for PCC, increasing the risk of preventable maternal and child health complications. Additionally, many individuals priorities other needs over PCC if they feel healthy, and there is reluctance among mothers to seek care, even for routine checkups when ill.

“Regarding the affordability of PCC costs, a mother with a low income might not be able to afford to pay, especially for some lab tests and imaging procedures” ***(32 years old mother, 5th grade, IDI)***

*“If it has a cost, those who cannot afford it would miss the service, which could contribute to the occurrence of maternal and child health complications that could have been prevented otherwise “****(29 years old mother, 10th grade, IDI)***

Mothers reported that long distances to health facilities and a lack of money for transportation often lead them to miss PCC services.

“The health facility may be too far from your house, you may face a shortage of money, and you

may be unable to walk there. In that case, how are you supposed to get to the health facility? If you do not have transportation costs, it is necessary to stay at home. Lack of money will prevent you from doing many things. Then, you become stressed and decide to be ignorant" ***(35 years old mother, not attended school, IDI)***

Nearly all mothers and HCPs agreed that PCC services should be accessible to individuals, regardless of economic status, to enhance maternal and child health, reduce costs, and promote equity. Participants stressed the importance of providing these services free of charge, like other MCH programs, to support low-income mothers at higher risk of health complications.

"The service should be free of charge. If it has a cost, those who cannot afford it will miss the service, which could contribute to the occurrence of maternal and child health complications that could have been prevented otherwise" ***(29 years old mother, 10th grade, IDI)***

"The cost of service for PCC should be free, similar to the service provided for ANC. If there is a fee, the community will refuse it. The government should provide subsidies and exempt services related to PCC" ***(HEW, female, IDI)***

A HCP working at MCH said that providing free PCC services is also difficult forthat providing free PCC services is also difficult for the health facility balanced payment may be good for sustaining the services.

"If it is a free service, the health facility may also suffer, but it should be at a reasonable price. For example, a sugar test results in 70 or 80 birr. This should be reduced" ***(Public health professional, male, IDI)***

Scarcity of medicines and medical equipment

The majority of both HCPs and mothers highlighted that shortages of medical equipment and

medicines such as iron and folic acid supplements, anti-hepatitis vaccines, immunoglobulin, laboratory reagents, essential drugs, and diagnostic tests like ultrasound pose significant challenges to PCC services, particularly at health centers. Similarly, they mentioned that due to the scarcity of medical supplies and medications, women are frequently referred to hospitals, exacerbating the situation, especially amid the crisis in the study area (Tigray). A participant working in the delivery unit highlighted a concerning trend of congenital anomalies, which they suspect may be linked to the shortage of folic acid supplements. Additionally, participants reiterated the challenges posed by the lack of guidelines and the shortage of HCPs

"Most of the preconception care services, such as iron and folic acid supplements, ultrasounds, and lab tests for HIV/STI, are not available at the health center" ***(29 years old mother, 10th grade, IDI)***

There is no guideline and a shortage of healthcare providers. …….."A mother who is Hepatitis positive should get Hepatitis vaccine and immunoglobin. But we don't have this drug in the facility" ***(MD, male, IDI)***

4. Opportunities on PCC services

Existing community platforms: Participants emphasized the crucial role of the HEP in ongoing PCC awareness at the community level. They suggested enhancing this program by involving HEWs with WDGs and village health leaders. Participants also highlighted that home-to-home visits strengthen PCC, as some women may feel uncomfortable discussing their desire to conceive openly.

"To make the program effective, it is essential to support WDGs and HEWs. In my previous work, I have seen the significant impact of WDGs in addressing public health issues. They have detailed community knowledge and can provide valuable information about vaccinated children,

pregnant mothers, and other relevant data. By working with community leaders and WDGs, we can increase the acceptance and success of the program" ***(Midwifery professional, female, IDI)***

A Health extension worker said:

"The home-to-home visit should be well strengthened because the woman might have felt ashamed to disclose her desire to get pregnant" ***(HEW, female, IDI)***

An MCH expert said *"A family health card is one. The service could be incorporated into the family health card checklist for the woman to read herself or to be read for her by her kids to improve their awareness"****(Public health professional, male ,KII)***

Participants suggested that marriage certificate issuance venues could be effective platforms for PCC education and counseling. These venues offer couples an entry point for information and provide an opportunity to counsel them in advance, linking eligible women to health facilities.

"Places where marriage certificate is provided would be even much better and of high importance to provide PCC service because it gives an opportunity for the couples to get the information before the start of sexual intercourse" **(35 years old mother, 10 th grade, IDI)**

Social networks such as civic societies, women's development groups, and farmer's associations are effective for community mobilization and spreading information. Participants highlighted their role in increasing PCC awareness and uptake through education and counseling at local venues including churches, mosques, and traditional healing places.

We can use the existing social networks such as civic societies (e.g. women's associations), women's development army, pregnant women forum…etc. Those social networks are the closet to the community; they can provide education to the mothers. First, the social networks need to be well aware of PCC and then provide roles and responsibilities to teach and mobilize mothers

to utilize the services. The social networks are very helpful in strengthening and decentralizing preconception care services ***(29 years old mother, 10th grade, IDI)***

SMART Start model: The SMART Start model focuses on financial preparation and achieving optimal health before pregnancy through contraception, ensuring healthy children. Participants noted that this model highlights the importance of preparation for pregnancy, akin to pre-pregnancy care. To raise awareness of PCC and uptake, the services would be integrated into the SMART Start model program as an entry point for couples, particularly adolescent couples, through activities conducted by WDGs, HEWs, and other social networks.

*SMART Start is very good especially because it advocates for adolescent girls to use contraceptives, and it creates awareness before pregnancy so that we can use it. Similarly, as you have mentioned very well, women's development groups can be used as an entry path way as we know it is vital to be aware of adolescent girls regarding preconception care (M****idwifery professional, male, KII)***

Perceived desire of PCC services: Though there would be a fear of workload, almost all HCPs appreciated the services' benefit in preventing maternal and neonatal problems.

…"There is also a higher need for PCC in the professional that can be described as an opportunity" ***(Public health professional, male, IDI)***

Its necessity is unquestionable! ***(Public health professional, male, KII)***

In addition, the majority of participants emphasized that, although these services are a new concept within the health system, mothers—especially those at high risk—have provided positive feedback.

"If the service is available plenty of mothers have a wish to get the service you desired" ***(35***

years old mother, not attended school, IDI)

This is both appropriate and acceptable. Had education arrived to us in this form, we would not be afflicted by the illnesses and issues that we currently face. We'll say "Amen" to this, bring it down to our people, and persuade them to start working on it. That's a smart idea ***(39 years old mother, 3rd grade, FGD)***

5. Perceived suggestions on PCC services

Home-based initative: Participants pointed out that women intending to become pregnant need to prepare at home in terms of food and money to ensure adequate nutrition, transportation, medications, and other necessities before and during pregnancy. Additionally, some participants emphasized the importance of communicating with their husbands to achieve a common understanding about the intention to get pregnant, being mentally prepared, and avoiding bad habits such as alcohol (including local beverages like (“Siwa, Areki, and Myes”), smoking, and other addictive substances as pre-pregnancy care.

Before her pregnancy, she should have a plan and be prepared economically; other work-related activities will help her overcome the problems that may arise following pregnancy.

In her home, she should communicate and have a common understanding with her husband about her pregnancy. When she plans to be pregnant, she should also avoid bad habits such as alcohol, smoking, and other addictive substances (hashish), and not be comfortable working during pregnancy in her home ***(Family health professional, female, KII)***

Use various communication strategies: All participants agreed that creating awareness for the infant program (PCC) through education on various platforms and social networks should be the first step to improving awareness of PCC and uptake. They emphasized the need for clear, culturally sensitive education on preparation, seeking advice, and understanding preconception

risks to address community beliefs, myths, and misconceptions. Utilizing media such as posters, billboards, and leaflets at community and facility levels is crucial for enhancing awareness. Once the community understands the importance of PCC, they will be more likely to seek it out independently.

……"Anyway, they have to work on awareness creation. Education on the causes and risk factors should be given. Education can be provided in the facilities, in groups, and in the community" ***(28 years old mother, 8th grade, IDI)***

Any cultural or religious barriers are better addressed through education. We should educate the community in an understandable way for them. If we do so, I think the elders, community, and religious leaders would say yes, it is essential; there are only benefits, not harms; let's make use of it; and so on, provided that we educate them. I don't expect the other way around. ***(35 years old mother, 10th grade, IDI)***

A mother from the FGD said, "I prefer that most of the time, PCC *be given on Sunday because both wife and husband could be available at home, and on this day, most people are off from work. If the HEW/WDA conduct home visits after 11:00 AM to provide education about PCC, half an hour is enough"* ***(28 years old mother, diploma, FGD)***

The study participants pointed out that education should be specific, pragmatic, and model-based.

Yes of course: it is often better to see than hear to easily understand and understand everything by looking at a picture. I think it would be better if he did the vegetables or other things diet in pictures ***(Midwifery professional, female, IDI)***

If you give pretend or picture-based lessons or drama-assisted lessons, women understand easily ***(Public health professional, male, IDI)***

"Our education should be unique, which means that if we are pregnant, for example, from pre-pregnancy to pregnancy, our education should not be the same as the previous by saying "listen to her", I mean it should be practical" ***(Public health professional, male, KII)***

Participants suggested several strategies to increase awareness and uptake of PCC, emphasizing the use of various media. Broadcast media like radio and television are particularly effective due to their widespread availability. Print media, including posters, billboards, and brochures, should be used at both community and facility levels. While HEWs play a crucial role, media influence is even more critical, as HEWs cannot reach every area. One participant noted that media can significantly raise awareness, stating that if people hear about PCC on the radio or television, they are more likely to seek health services and receive education through printed materials.

"The greatest power is the media. Whether it is through television, radio, and magazines, the media is very crucial if you want to change the perception of the community about the PCC" ***(MD, male, IDI)***

"Media has a big role. Our community follows the media. Although the role of HEWs is the biggest, the influence of media is more critical than anything because they can't reach every corner" ***(Nursing professional, female, IDI)***

Although orientation on PCC has recently commenced, it remains a new program. Consequently, nearly all individuals involved in the healthcare system, from HEWs to district health experts, possess limited knowledge about PCC. Therefore, both short-term and long-term training are essential for effectively providing these services. All participants emphasized that training and guidelines are crucial for successfully delivering PCC services.

"Training on PCC packages is required for all actors involved in the health care system, starting from the HEW to district/woreda health experts (health care professionals)" ***(Family health***

professional, female, KII)

Community engagement: Some participants emphasized that creating significant awareness about preconception care at the community level requires special efforts, mainly focusing on extensive information dissemination and community engagement.

Special efforts are required, including robust community mobilization and active participation ***(HEW, female, IDI)***

Community health facility Linkage: in general, participants noted that establishing a linkage between the community and the health facility, as well as within the health facility to selected units providing PCC, using referral slips, would be vital for enhancing the uptake of PCC services and reducing delays in the health facility.

An MCH expert said *a referral slip is necessary. Once the eligible woman is identified, the referral slip can be used to link to the service. However, one thing that needs to be taken into consideration is whether or not it will be a free service. Maybe it could have some effect if it is payable service* ***(Public health professional, male, KII)***

Use high-risk mothers as role models for awareness creation: Some participants suggested that mothers with a history of APOs could serve as role models to educate women in women's development groups, pregnancy forums, and similar platforms. This approach could motivate the community to improve awareness and uptake of PCC.

"I consumed three or four beers until I gave birth while I was pregnant. Here, I found myself teaching others and myself about mental retardation, a health issue that my child had developed. Even if you have to consider it before getting pregnant, you should not drink while pregnant. However, since my son experienced it, I have been telling people about it once more. Since a baby is like a piece of paper and can be easily injured, I think that my son's alcohol abuse

contributed to his mental retardation" ***(27 years old mother, 10th grade, IDI)***

"She also influenced other women who had difficulty conceiving. i.e., other women also seek health facilities after taking experience from my sister" ***(30 years old mother, diploma, FGD)***

Services integration

HCPs unanimously recommended integrating PCC into existing services, including voluntary counseling and test, family planning units, cervical cancer screening, youth-friendly services, ANC, ART clinics, post-abortion care, EPI, and under-five clinics. This approach aims to provide services to educate women of reproductive age who visit health facilities for other reasons, using the RLP tool to identify those eligible for PCC services effectively.

"All people who visit a health facility should get information on PCC. We can use health facilities as a means to disseminate information and create awareness" ***(HEW, Female, IDI)***

"As a PCC, I believe that any female patient in the reproductive age group should be able to see you whenever they need something, like an abortion, an STI test, an family planning, a pregnancy test, or a PNC. Right now, all women should receive PCC counseling" ***(MD, male, IDI)***

Some participants suggested that PCC services should be provided as standalone services in a room, which would be more user-friendly. Integrating PCC with other services might make women uncomfortable. However, due to limited space, staff, and resources, offering PCC under the ANC unit could be beneficial, as it allows for the observation and advice on pregnancy-related abnormalities. Some participant suggests that PCC services could be offered alongside family planning services, where women typically seek to prevent pregnancy, providing an opportunity to discuss PCC.

Discussion

This study examined the experiences, challenges, and opportunities of PCC services in Tigray, Ethiopia following the government's prioritization of PCC as a strategic initiative and the introduction of new guidelines (26) (37) (38). While previous qualitative research on PCC focused solely on barriers to its uptake (39), this study took a broader approach by exploring the awareness, experiences, challenges, and opportunities related to PCC services from the perspectives of various participants.

The study identified key themes related to PCC services, including awareness (information and perceived benefits) and experience (practices of PCC interventions and home-based preparation). Participants also explored perceived challenges, such as traditional beliefs and misconceptions, fragmented service delivery, reluctance to disclose conception intentions, inadequate counseling on contraceptive services, societal norms, heavy workloads, high costs, and shortages of medicines and medical equipment. Additionally, they noted perceived opportunities, such as a strong interest in PCC services and the availability of community platforms. Suggestions for improvement included implementing home-based initiatives, adopting diverse communication strategies, involving high-risk mothers as role models, fostering community engagement, strengthening links between communities and healthcare facilities, and integrating PCC services within the healthcare system.

In the study, except a few high-risk mothers, most mothers lack awareness of PCC services, which is consistent with previous studies (24, 39-41). Many people may be unfamiliar with PCC services because the program was introduced only recently (26) (37) (38) and has not yet been fully integrated into the healthcare system. This is especially evident within the health extension program, which is vital for increasing community awareness. This study highlights the issue, showing that HEWs possess less information about the program compared to other HCPs.

Furthermore, HCPs often view PCC as an optional benefit rather than a standard service, leading to limited investment and delayed care-seeking until pregnancy is confirmed. Such neglect can hinder health-seeking behavior and the utilization of services. Overall, participants emphasized that enhancing PCC awareness should be a government priority, leveraging community engagement through social networks like civic organizations, women's groups, and farmers' associations, which are effective for mobilization and information sharing. Home-based preparation was identified as a promising foundation, alongside diverse communication methods and integrating PCC services into healthcare facilities to improve awareness and utilization. Evidence also showed that to enhance PCC awareness, massive public awareness campaigns and education through print media, social forums, and government-led initiatives including integrating PCC into the healthcare services (22, 42).

This study revealed that while most HCPs had some knowledge of PCC services, their practice was limited. The services mainly focused on specific components targeting high-risk mothers such as contraceptive counseling for pregnancy delay, medication safety, substance use counseling, and folic acid supplementation. However, most interventions recommended by the WHO and Ethiopia's national PCC guideline were not practiced (2) (38). This is consistent with previous findings from African countries, where PCC was implemented under inaccessible guidelines and primarily driven by initiatives targeting high-risk women with a limited focus on interventions such as dietary modification counseling (25) (43) (44). The limited practice of PCC is influenced by several factors such as the presence of a relatively new program, HCPs' lack of adequate training, and PCC guidelines not being easily accessible. Moreover, the government prioritizes ANC over PCC, leading HCPs to view PCC as a supplementary rather than a routine service. Evidence suggests that accessible guidelines and regular use of RLP tools can help

HCPs prioritize PCC and emphasize preconception care for women (22) (25). Hence, prioritizing PCC and integrating package-based services into routine care for all women of reproductive age using RLP tools could significantly enhance awareness and utilization.

The study revealed that traditional beliefs often compel women to keep their conception desires private avoiding discussions about PCC. Pregnancy is commonly regarded as a divine gift from St. Mary or God, and many women believe that expressing a desire to conceive before pregnancy contradicts divine will, making such discussions socially and personally taboo. This is consistent with other studies elsewhere (39, 45), indicating that women often perceive conception as a natural event requiring no preparation, resulting in limited communication with HCPs and missed opportunities to utilize PCC services. Evidence suggests that addressing traditional beliefs about conception requires collaboration between health institutions and religious organizations (39). Additionally, integrating preconception health discussions into routine healthcare, beginning with school-based services, has been proposed as a potential solution (46). In this study, participants indicated that women prefer receiving PCC at health posts and health centers, as well as through community-based services, because these settings align with cultural norms and are conveniently located, making it easier for women to discuss personal matters.

Evidence indicates that, without adequate counseling, clients often turn to friends and family for information. Unfortunately, this information is frequently influenced by misinformation (47). For example, in this study, misconceptions about contraceptives, such as the Depo-Provera injection, have led many women to be discouraged or even prevented from using them. These misconceptions, including unfounded fears that contraceptives may cause infertility or congenital anomalies, present significant challenges to raising awareness about PCC and improving its

uptake. HEWs also give due emphasis to the maternal continuum of care; however, they do nothing for PCC as a package that could be considered a missed opportunity.

Evidence shows that family planning unit is vital in improving preconception health by assessing pregnancy intentions and addressing misconceptions through tools like the RLP (48). Therefore, integrating PCC into family planning and antenatal services strengthens the HEP with culturally sensitive education, and utilizing health visits for PCC education can significantly enhance services and reduce misconceptions about contraceptive use.

Involving husbands in PCC through screenings and counseling is vital for reducing risks and promoting healthier pregnancies, as emphasized by Ethiopian (38) and Chinese national guidelines (49). Furthermore, women who made decisions independently or in partnership with their husbands were more likely to utilize PCC services (50) (51) . Our findings showed that husbands often oppose women's PCC, particularly in rural and remote areas. Poor parental behaviors before conception are linked to increased illness and mortality in offspring, while healthier habits can significantly improve pregnancy outcomes. Paternal preconception care emphasizes men's direct contributions to child health, such as genetic and epigenetic factors, lifestyle choices, and environmental exposures, as well as indirect influences through partner health and relationships (52). The paternal origins of health and disease model defines (53) the preconception population as all reproductive-age men and women, highlighting the importance of paternal health. Given men's crucial role in reproductive health decisions, couple-based counseling is strongly recommended (47).

The study found that poor socioeconomic status including service costs and distance hinders PCC use with some viewing pregnancy as a luxury due to basic needs and regional conflicts. These findings align with results from various other studies (39) (54). The implication is that

even when women acknowledge the need for PCC and wish to access it, they face additional challenges such as service charges and transportation costs. Participants suggested women prefer receiving PCC at health posts and health centers alongside community-based services. They feel more comfortable discussing personal matters in culturally familiar local settings rather than hospitals. This preference helps address transportation costs, offers more apparent education, and overcomes barriers related to disclosing pregnancy intentions; which is a major obstacle to PCC utilization. Besides, the current study emphasizes that HCPs and mothers strongly advocate for making PCC services free, as they are part of the maternal continuum of care. This could improve access, particularly for low-income mothers, enhance maternal and child health, reduce costs, and promote equity. Other study in China support this, with China's National Free Preconception Health initiative, for example, reaching over 95% of target couples with preconception health education (49). Additionally, free maternal healthcare including PCC aligns with the WHO's recommendation to eliminate financial barriers and ensure equitable access to healthcare for all. It supports achieving SDG 3.1, which aims to reduce maternal mortality to fewer than 70 per 100,000 live births by 2030 (55).

To enhance awareness of PCC and uptake, the study suggests using marriage certificate settings to provide couples with information and pre-counseling, thereby connecting eligible women to health facilities. This approach could be practical if the Ethiopian government in collaboration with religious leaders commits to issuing marriage certificates particularly for newly married couples. This recommendation aligns with findings from a study in Bangladesh (56). Marriage certificate settings present an ideal opportunity to introduce PCC services as couples are often in the early stages of planning their future. This universal process provides an equitable platform to educate couples on reproductive health, family planning, genetic risks, and the importance of

preparing for a healthy pregnancy.

In Ethiopia, the PCC program was integrated into the reproductive health strategy for 2020–2024/25(26), and the components of PCC interventions were incorporated into the 2022 ANC guidelines (37). Additionally, the 2024 PCC guidelines recommend providing integrated services to women planning to conceive within three months (38). However, in this study, PCC services were only partially implemented hindered by the absence of PCC guidelines, limited government focus, inadequate training, and fragmented service delivery which heavily relied on women's initiation. PCC services was neither prioritized nor adequately resourced, resulting in inconsistent practices and insufficient attention to this critical care area. High-risk women were often not offered PCC unless they explicitly requested it, and PCC was not routinely utilized to assess reproductive intentions even for women who had stopped using contraception to conceive. These findings align with studies from other parts of Africa (44, 57). In contrast, studies (49, 58) indicated that fully implemented services supported by established protocols and strong government backing, improve service utilization compared to fragmented approaches. Overall, the Ethiopian government should prioritize PCC and ensure its full implementation by integrating it into the existing healthcare system. This requires the inclusion of comprehensive PCC guidelines, protocols, and assessment tools as part of routine clinical practice.

According to the Motivational Theory of Role Modeling, role model-based interventions are highly effective in encouraging others to accept new services (59). In our study, participants suggested that high-risk mothers could act as role models to enhance awareness and encourage using PCC services.

In the study, many participants suggested integrating PCC with existing health services like ANC, voluntary counseling and testing , ART clinics, family planning units, and more targeting

women of reproductive age using the RLP tool. This is also in concurrent with other studies in Africa (4, 5). Most preconception counseling was provided opportunistically by HCPs working in gynecology clinics or family planning settings (60). Integrating services can improve access for women with limited awareness of available options, those not actively planning or considering pregnancy, those with low health-seeking behaviors, and those without identified health issues. By routinely offering these services to all women of reproductive age through an integrated approach that includes the RLP tool, the uptake of PCC services can be improved (22, 25).

Study limitations and strengths: This first study in Ethiopia explores PCC services from multiple perspectives, including front-line HCPs in urban and rural areas. The findings significantly contribute to developing locally appropriate programs to increase awareness of PCC and uptake. However, due to resource constraints, our study did not explore the perspectives of husbands, religious leaders, and women without pregnancy histories, emphasizing the need for further research. We held open discussions and regular meetings among the researchers to maintain trustworthiness. However, because the naming of the service is unfamiliar to the community, some women may have confused PCC with ANC, potentially leading to exaggerated responses.

Conclusions

This study reveals that most women lack awareness about PCC. HCPs deliver fragmented PCC interventions without using standardized guidelines or assessment tools focusing mainly on high-risk cases. Key challenges and opportunities identified include fragmented PCC services, traditional beliefs and misconceptions, inadequate counseling on contraceptive services, social

influences, perceived demand for PCC, and the potential to leverage existing community platforms. Integrating services into the existing community platforms, particularly within the HEP, is recommended to enhance awareness of PCC. Diverse communication strategies, including media campaigns and educational initiatives, should address traditional beliefs, misconceptions, and low community awareness. Additionally, participants suggested to strengthening the exisiting home-based initiatives and leveraging high-risk mothers as role models to promote awareness of PCC. Moreover, a package-based approach need to be implemented by integrating PCC as a continuum of maternal care. Introducing the concept of RLP is essential, enabling routine use of RLP tools to identify eligible women for these services. These findings provide valuable insights for Ethiopia's Ministry of Health, guiding policymakers, and program designers to enhance PCC utilization. Additionally, innovative interventions should focus on establishing package-based services, creating demand, and strengthening service linkages for better outcomes.

Abbreviations

ANC: Ante-natal Care

APOs: Adverse Pregnancy Outcomes

ART: Anti retro-viral therapy

CDC: Centers for Disease Control and Prevention

EPI: Expanded Program Immunization

FP: Family planning

HCPs: Healthcare providers

HEP: Health Extension Program

HEWs: Health Extension Workers

MNCH: Maternal, Neonatal and child health

OPD: Out Patient Department

PCC: Preconception Care

RLP: Reproductive Life Plan

SDGs: Sustainable Development Goals

VCT: Voluntary counseling and Test

WDGs: Women Development Group

WHO World Health Organization

References

1. Dean SV, Lassi ZS, Imam AM, Bhutta ZA. Preconception care: closing the gap in the continuum of care to accelerate improvements in maternal, newborn and child health. Reproductive Health. 2014;11:1-8.
2. World Health Organization. Meeting to develop a global consensus on preconception care to reduce maternal and childhood mortality and morbidity: World Health Organization Headquarters, Geneva, 6–7 February 2012: meeting report. 2013.
3. World Health Organization. Preconception care. WHO Regional Office for South-East Asia; 2014.
4. Health NDo. Guidelines for Maternity Care in South Africa: A Manual for Clinics, Community Health Care Centres and District Hospitals. Pretoria. . 2015;Report No.: 4th edition.
5. Health MoP, Sanitation, Services MoM. National Reproductive Health Strategy 2009–2015. 2009.
6. Ethiopia Moh. National Antenatal Care Guideline, Ensuring Positive Pregnancy Experience. 2022.
7. Ethiopia Moh. National Preconception care Guideline, Ensuring a risk free conception experience 2024.
8. World Health Organization. Trends in maternal mortality 2000 to 2017: estimates by WHO, UNICEF, UNFPA, World Bank Group and the United Nations Population Division: executive summary. World Health Organization; 2019.
9. Quenby S, Gallos ID, Dhillon-Smith RK, Podesek M, Stephenson MD, Fisher J, et al. Miscarriage matters: the epidemiological, physical, psychological, and economic costs of early pregnancy loss. The Lancet. 2021;397(10285):1658-67.
10. Chawanpaiboon S, Vogel JP, Moller A-B, Lumbiganon P, Petzold M, Hogan D, et al. Global, regional, and national estimates of levels of preterm birth in 2014: a systematic review and modelling analysis. The Lancet Global Health. 2019;7(1):e37-e46.
11. World Health Organization. Congenital anomalies. Fact sheet N 370. Media centre World Health Organization, Geneva; 2014 http://www who int/mediacentre/factsheets/fs370/en/ Accessed 10 Jan 2015. 2012.

12. Moges N, Anley DT, Zemene MA, Adella GA, Solomon Y, Bantie B, et al. Congenital anomalies and risk factors in Africa: a systematic review and meta-analysis. BMJ Paediatrics Open. 2023;7(1).
13. Tamirat KS, Sisay MM, Tesema GA, Tessema ZT. Determinants of adverse birth outcome in Sub-Saharan Africa: analysis of recent demographic and health surveys. BMC Public Health. 2021;21(1):1-10.
14. Csa I. Central statistical agency (CSA)[Ethiopia] and ICF. Ethiopia demographic and health survey, Addis Ababa, Ethiopia and Calverton, Maryland, USA. 2016;1.
15. EPHI I. Ethiopian Public Health Institute (EPHI)[Ethiopia] and ICF. Ethiopia Mini Demographic and Health Survey 2019: Key Indicators. 2019.
16. Stierman EK, Ahmed S, Shiferaw S, Zimmerman LA, Creanga AA. Measuring facility readiness to provide childbirth care: a comparison of indices using data from a health facility survey in Ethiopia. BMJ Global Health. 2021;6(10):e006698.
17. Berihu BA, Welderufael AL, Berhe Y, Magana T, Mulugeta A, Asfaw S, et al. High burden of neural tube defects in Tigray, Northern Ethiopia: Hospital-based study. PLoS One. 2018;13(11):e0206212.
18. Gesesew HA, Berhe KT, Gebretsadik S, Abreha M, Haftu M. Fistula in War-Torn Tigray: A Call to Action. International journal of environmental research and public health. 2022;19(23):15954.
19. Luber J. Better than Bullets: Ethiopia is Committing War Crimes by Starving Civilian Populations in the Ethiopian Civil War. Am U Int'l L Rev. 2021;37:701.
20. Gesesew H, Berhane K, Siraj ES, Siraj D, Gebregziabher M, Gebre YG, et al. The impact of war on the health system of the Tigray region in Ethiopia: an assessment. BMJ Global Health. 2021;6(11):e007328.
21. Legesse AY, Teka Tseghay H, Abraha HE, Fisseha G, Ebrahim MM, Tsadik M, et al. Maternal mortality during war time in Tigray, Ethiopia: A community-based study. BJOG: An International Journal of Obstetrics & Gynaecology. 2024;131(6):786-94.
22. Ukoha WC, Mtshali NG, Adepeju L. Current state of preconception care in sub-Saharan Africa: A systematic scoping review. African Journal of Primary Health Care & Family Medicine. 2022;14(1):3096.
23. Woldeyohannes D, Tekalegn Y, Sahiledengle B, Hailemariam Z, Erkalo D, Zegeye A, et al. Preconception care in sub-Saharan Africa: A systematic review and meta-analysis on the prevalence and its correlation with knowledge level among women in the reproductive age group. SAGE Open Medicine. 2023;11:20503121231153511.
24. Ayele AD, Belay HG, Kassa BG, Worke MD. Knowledge and utilisation of preconception care and associated factors among women in Ethiopia: systematic review and meta-analysis. Reproductive health. 2021;18:1-15.
25. Kassa A, Human S, Gemeda H. Level of healthcare providers' preconception care (PCC) practice and factors associated with non-implementation of PCC in Hawassa, Ethiopia. Ethiopian journal of health sciences. 2019;29(1).
26. Health EMo. Health Sector Transformation Plan II (HSTP II): 2020/21-2024/25 (2013 EFY-2017 EFY). MOH; 2021.
27. Atrash H, Jack B. Preconception care to improve pregnancy outcomes: the science. Journal of Human Growth and Development. 2020;30(3):355-62.
28. Mason E, Chandra-Mouli V, Baltag V, Christiansen C, Lassi ZS, Bhutta ZA. Preconception care: advancing from 'important to do and can be done'to 'is being done and is making a difference'. Reproductive health. 2014;11:1-9.
29. Goossens J, De Roose M, Van Hecke A, Goemaes R, Verhaeghe S, Beeckman D. Barriers and facilitators to the provision of preconception care by healthcare providers: a systematic review. International journal of nursing studies. 2018;87:113-30.

30. Tong A, Sainsbury P, Craig J. Consolidated criteria for reporting qualitative research (COREQ): a 32-item checklist for interviews and focus groups. International journal for quality in health care. 2007;19(6):349-57.
31. Abraha H, Ebrahim M, Teka H, Yemane A, Mulugeta A. Armed conflict drives most births to occur out of health facilities: evidence from Tigray, Ethiopia. drugs. 2023;2:3.
32. Tsegaye B, Kassa A. Prevalence of adverse birth outcome and associated factors among women who delivered in Hawassa town governmental health institutions, south Ethiopia, in 2017. Reproductive health. 2018;15(1):1-10.
33. Palinkas LA, Horwitz SM, Green CA, Wisdom JP, Duan N, Hoagwood K. Purposeful sampling for qualitative data collection and analysis in mixed method implementation research. Administration and policy in mental health and mental health services research. 2015;42:533-44.
34. Wong LP. Focus group discussion: a tool for health and medical research. Singapore Med J. 2008;49(3):256-60.
35. Guest G, MacQueen KM, Namey EE. Applied thematic analysis: sage; 2012.
36. Vaismoradi M, Turunen H, Bondas T. Content analysis and thematic analysis: Implications for conducting a qualitative descriptive study. Nursing & health sciences. 2013;15(3):398-405.
37. health_Ethiopia Mo. Ensuring Positive Pregnancy Experience. National Antenatal Care Guideline. 2022.
38. health_Ethopia Mo. Ensuring a risk free conception experience! National Preconception care Guideline. 2024.
39. Teshome F, Kebede Y, Abamecha F, Birhanu Z. Why do women not prepare for pregnancy? Exploring women's and health care providers' views on barriers to uptake of preconception care in Mana District, Southwest Ethiopia: a qualitative study. BMC Pregnancy and Childbirth. 2020;20:1-13.
40. Chutke AP, Doke PP, Gothankar JS, Pore PD, Palkar SH, Patil AV, et al. Perceptions of and challenges faced by primary healthcare workers about preconception services in rural India: A qualitative study using focus group discussion. Frontiers in Public Health. 2022;10:888708.
41. Siraha E, Mukona D, Zvinavashe M, Dodzo L. Perceptions of preconception care among pregnant women at Masvingo general hospital, Zimbabwe: a qualitative study. Journal of Midwifery and Reproductive Health. 2020;8(2):2220-9.
42. Okemo J, Temmerman M, Mwaniki M, Kamya D. Preconception care among pregnant women in an urban and a rural health Facility in Kenya: a quantitative study. International journal of environmental research and public health. 2020;17(20):7430.
43. Ojifinni OO, Ibisomi L. "Is the health system ready?" A qualitative exploration of stakeholders' opinions about the feasibility of preconception care services in the Nigerian health system. Reproductive Health. 2022;19(1):153.
44. Ndou NP, Malwela T, Maputle MS, Raliphaswa NS, Mabasa L, Samie A, et al., editors. Factors Related to the Implementation of Preconception Care Recommendations in Selected Districts of Limpopo Province: A Qualitative Study. Healthcare; 2023: MDPI.
45. Bortolus R, Oprandi NC, Rech Morassutti F, Marchetto L, Filippini F, Agricola E, et al. Why women do not ask for information on preconception health? A qualitative study. BMC pregnancy and childbirth. 2017;17:1-11.
46. Barrett G, Smith SC, Wellings K. Conceptualisation, development, and evaluation of a measure of unplanned pregnancy. Journal of Epidemiology & Community Health. 2004;58(5):426-33.
47. Adongo PB, Tabong PT-N, Azongo TB, Phillips JF, Sheff MC, Stone AE, et al. A comparative qualitative study of misconceptions associated with contraceptive use in southern and northern Ghana. Frontiers in Public Health. 2014;2:137.
48. Klerman LV. Family planning services: an essential component of preconception care. Maternal and child health journal. 2006;10:157-60.

49. Xu J, Li X, Zhou Q. Nationwide-free preconception care strategy: experience from China. Frontiers in Public Health. 2022;10:934983.
50. Asresu TT, Hailu D, Girmay B, Abrha MW, Weldearegay HG. Mothers' utilization and associated factors in preconception care in northern Ethiopia: a community based cross sectional study. BMC pregnancy and childbirth. 2019;19:1-7.
51. Abrha MW, Asresu TT, Weldearegay HG. Husband support rises women's awareness of preconception care in Northern Ethiopia. The Scientific World Journal. 2020;2020(1):3415795.
52. Cardenas SI, Morris AR, Marshall N, Aviv EC, Martínez García M, Sellery P, et al. Fathers matter from the start: The role of expectant fathers in child development. Child Development Perspectives. 2022;16(1):54-9.
53. Soubry A. POHaD: why we should study future fathers. Environmental epigenetics. 2018;4(2):dvy007.
54. Lang AY, Bartlett R, Robinson T, Boyle JA. Perspectives on preconception health among migrant women in Australia: a qualitative study. Women and Birth. 2020;33(4):334-42.
55. Sachs JD. From millennium development goals to sustainable development goals. The lancet. 2012;379(9832):2206-11.
56. Jahan S, Shermin S. Retracted: Preconception Care through Religious Leaders in Bangladesh–A Novel Approach. Delta Medical College Journal. 2018;6(2):59-61.
57. Ukoha WC, Mtshali NG, editors. Perceptions and practice of preconception care by healthcare workers and high-risk women in South Africa: A qualitative study. Healthcare; 2021: MDPI.
58. Du L, La X, Zhu L, Jiang H, Xu B, Chen A, et al. Utilization of preconception care and its impacts on health behavior changes among expectant couples in Shanghai, China. BMC Pregnancy and Childbirth. 2021;21:1-8.
59. Morgenroth T, Ryan MK, Peters K. The motivational theory of role modeling: How role models influence role aspirants' goals. Review of general psychology. 2015;19(4):465-83.
60. Shawe J, Delbaere I, Ekstrand M, Hegaard HK, Larsson M, Mastroiacovo P, et al. Preconception care policy, guidelines, recommendations and services across six European countries: Belgium (Flanders), Denmark, Italy, the Netherlands, Sweden and the United Kingdom. The European Journal of Contraception & Reproductive Health Care. 2015;20(2):77-87.

 Click here to access/download;Figure;Figure.tiff

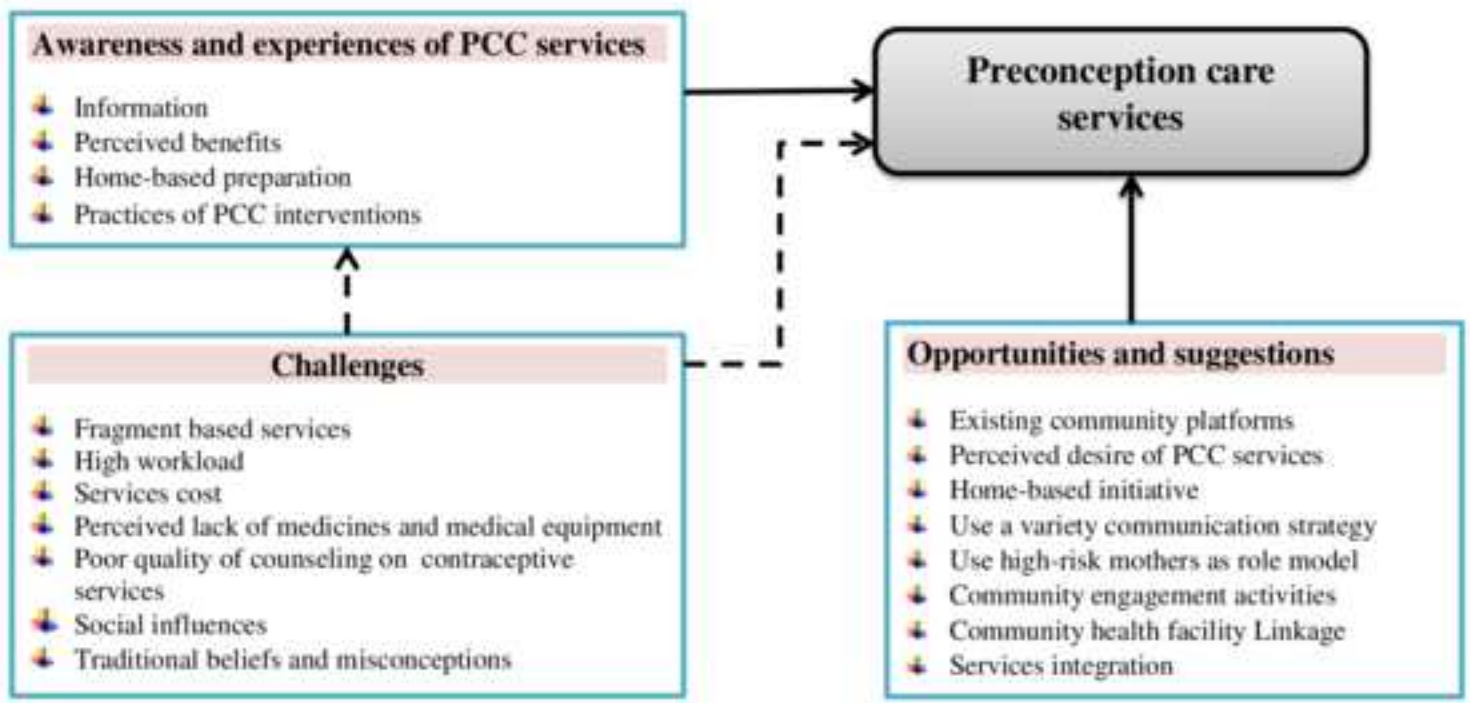

Click here to access/download
Supporting Information
PLOS questionnaire.docx

Click here to access/download
Supporting Information - Compressed/ZIP File Archive
Translated files.zip

Awareness and uptake of preconception care services in Northern Ethiopia – a qualitative exploration of experiences, challenges, opportunities, and prospects

Policy Implications of Preconception Care Services: Experiences, Challenges, and Opportunities in Tigray, Northern Ethiopia; an Exploratory Qualitative Study

Formatted: Line spacing: Double

Gebremedhin Gebreegziabher Gebretsadik[1, 2*], Alemayehu Bayray Kahsay [2], Andargachew Kassa [4], Amanuel Gessessew [3], Zohra Lassi, [5, 6], Afework Mulugeta [2]

Formatted: Line spacing: Double

*Corresponding author:

Email: gebremedhingebretsad@gmail.com (GG)

[1] Department of Midwifery, College of Medicine and Health Sciences, Adigrat University, Tigray, Ethiopia

[2] School of Public Health, College of Health Sciences, Mekelle University, Tigray, Ethiopia

[3] School of Medicine, College of Health Sciences, Mekelle University, Tigray, Ethiopia

[4] School of Public Health, College of Medicine and Health Sciences, Hawassa University, Ethiopia

[5] School of Public Health, Faculty of Health and Medical Sciences, University of Adelaide, Australia

Formatted: Line spacing: Double

[6] Robinson Research Institute, University of Adelaide, Australia

*Corresponding author:

Email**:** gebremedhingebretsad@gmail.com (GG)

Abstract:

Introduction~~:~~

Preconception care (PCC) has emerged as a key component of maternal continuum care ~~healthcare~~ worldwide, focusing on reducing poor pregnancy outcomes.~~ Despite evidence supporting its effectiveness, this critical opportunity remains largely overlooked in developing countries, including Ethiopia~~. Improving services requires addressing opportunities and challenges within the health system, but in Ethiopia it is often a neglected service .~~Therefore~~Hence, this study ~~aims to~~ explores the experiences, challenges, and opportunities related to preconception care services in Tigray, Northern Ethiopia.

Methods~~:~~

We conducted an exploratory qualitative study involving 21 in-depth interviews with mothers who experienced adverse pregnancy outcomes and healthcare ~~providers~~providers, who work in maternal, neonatal, and child health, ~~including~~ and health extension workers. Additionally, we held ~~6~~ six focus group discussions with women who had ~~a~~ history of pregnancy. We also ~~and~~ conducted ~~10~~ = key informant interviews with 10 maternal, newborn and child health experts from regional health bureau~~s~~, district health offices, and ~~relevant~~ professional associations. The

study was conducted from January 26, 2024, to April 4, 2024~~,~~ across four rural districts and two urban areas in Tigray, northern Ethiopia. Discussions and interviews were audio-recorded, transcribed in to the local language "Tigrigna" then , translated into English, and thematically coded using ATLAS.ti v.7.5.4 software.

Results~~:~~

~~Some women have awareness of PCC services mainly among high risk, the majority healthcare providers, especially gynecologists and doctors, have some knowledge of PCC and understand its importance and provide some components of PCC interventions in a fragmented way, primarily targeting high-risk women. Challenges include limited awareness, misconceptions, and fear of contraceptive side effect, workloads, and poor quality family planning services. Opportunities include leveraging community platforms, the SMART Start model, and interest in PCC from both professionals and mothers. Suggested improvements include increased government focus, offering free PCC services, assigning responsible professionals, implementing home-based initiatives, using diverse communication strategies, and involving high-risk mothers as educational role models.~~

Some women, particularly those belonging to high-risk groups, are aware of PCC services. A significant proportion of healthcare providers, especially gynecologists and physicians have some knowledge of PCC, recognize its importance, and provide specific components of PCC interventions. However, these services are often delivered in a fragmented manner, primarily targeting high-risk women. Identified challenges include traditional beliefs and misconceptions, insufficient counseling on contraceptive services, social influences, service costs, high workloads, perceived shortages of medicines and medical equipment, and the fragmented nature of service delivery. Conversely, opportunities include utilizing existing community platforms

and an expressed desire for PCC services. Moreover, the use of diverse communication strategies, linking communities with health facilities, involving high-risk mothers as educational role models, and integrating package-based PCC services into the healthcare system were explored as perceived suggestions.

Conclusion ~~and recommendation:~~

With the exception of high-risk women, most women have little to no knowledge about PCC services. Furthermore, although many healthcare providers possess some understanding of PCC, they deliver only a limited range of interventions, primarily catering to self-initiated high-risk mothers. Challenges identified include traditional beliefs and misconceptions, inadequate counseling on contraceptive services, social influences, high service costs, and fragmented service delivery. Existing community platforms and the perceived desire for PCC services were highlighted as opportunities to enhance PCC services. Strategies such as utilizing diverse communication methods, involving high-risk mothers as role models, strengthening community engagement activities, and improving linkages between communities and health facilities were proposed. Additionally, promoting home-based self-care was explored as a suggestion for improving PCC services. Integrating package-based PCC services into the healthcare system to routinely serve all eligible women of reproductive age was recommended to improve both awareness and uptake of PCC. Finally, tailored interventions were deemed essential for improving PCC awareness and utilization both at the community and facility levels.~~Women have low awareness of PCC, and although most healthcare professionals possess some knowledge, they offer fragmented interventions mainly for high-risk groups. Challenges include limited awareness, misconceptions, and a perceived lack of need for PCC among low-risk~~

~~women. To enhance awareness and uptake, the study recommends, package based services, diverse communication strategies strengthening home based self-care, using high-risk mothers as role models, and integrating the SMART Start model into services. Innovative interventions are essential to boost both awareness and utilization of PCC services.~~

Key words: Preconception Care Services, Experiences, Challenges, Opportunities, ~~Policy Implications~~, Explorative Qualitative Study, Tigray

Introduction

Preconception care (PCC) has garnered significant attention globally, primarily due to its ability to reduce the risk of adverse pregnancy outcomes (APOs). The existing continuum of care lacks pre-pregnancy care, and PCC addresses this gap by mitigating parental risk factors before conception, thereby enhancing outcomes for both mothers and infants (1). To mitigate the burden of APOs, both the World Health Organization (WHO) and the Centers for Disease Control and Prevention (CDC) universally recommend PCC as an essential part of maternal healthcare. They advise that women intending to become pregnant should receive at least one preconception care checkup (2). Following CDC/WHO recommendations, several low- and middle-income countries (LMICs) including Bangladesh, the Philippines, and Sri Lanka ,have innovatively integrated PCC into their health systems (2), (3). Besides, some Sub-Saharan Africa(SSA) countries, like South Africa(4), Kenya (5) and Ethiopia (6) (7), have also incorporated PCC into their healthcare systems and are working to leverage this initiative to achieve the Sustainable Development Goals (SDGs) in a timely manner.

Worldwide, approximately 295,000 maternal deaths occur each year due to complications from pregnancy or childbirth (8). Additionally, there are around 23 million miscarriages (9), 14.8

million live preterm births(10), and 295,000 neonatal deaths caused by congenital anomalies annually(11). The prevalence of congenital anomalies in LMICs is significantly high, with 94% of cases being severe(12). Similarly, the burden of APOs in LMICs remains a significant global issue. For example, the burden of APOs in SSA is 29.7% (13). Ethiopia is among the LMICs with high rates of maternal and neonatal deaths, at 412 per 100,000 live births and 33 per 1000 live births, respectively (14, 15). Before the war, Tigray's health facilities ranked among the best in Ethiopia for maternal, newborn, and child health services(16). In the Tigray region, neural tube defects occur at a rate of 131 per 10,000 births (16). However, since the conflict began in Since the conflict began in November 2020, significant damage to the health system has severely disrupted maternal care and essential services. This has exacerbated preconception risks, including unwanted pregnancies, unsafe abortions, home deliveries, malnutrition, and increased maternal, newborn, infant, and child mortality rates (18-20). For instance, the maternal mortality rate during the conflict reached 840 deaths per 100,000 live births.(21). ~~In the Tigray region, neural tube defects occur at a rate of 131 per 10,000 births (16). The war has worsened preconception risks(17), with a maternal mortality rate of 840 per 100,000 live births during the conflict (18).~~

Despite the WHO's recognition of its feasibility in LMICs, PCC has not been successfully adopted in Africa (2). HIV testing, family planning, and contraceptive services are commonly utilized components in PCC interventions, while supplementation of folic acid is less ~~commonly utilized~~widly used (19). A recent review in SSA found that the uptake and knowledge of PCC were only 24.05% and 33.27% (20), respectively. A systematic review further revealed that in Ethiopia, only 30.95% of women had knowledge of PCC, with just 16.27% utilizing it (21), and the provision was low at 15% (22). Ethiopia has set ambitious targets under its reproductive

health strategic plan to align with SDG 3.1, aiming to significantly reduce preventable pregnancy-related morbidity and mortality by 2025 (23). These targets include increasing the proportion of pregnant women receiving PCC to 25%, reducing the neonatal mortality rate from 33 to 21, and decreasing the maternal mortality rate to 271 per 100,000 live births (23). However, information is lacking on how the healthcare system has implemented PCC services.

Studies have indicated that identifying practical and feasible interventions based on local contexts is essential for raising awareness and improving PCC service adoption (24, 25). A qualitative study in high-income countries revealed that unfavorable attitudes, a lack of knowledge about PCC, limited resources such as time and guidelines, and unclear responsibilities for providing PCC are barriers to its provision [49]. In Southwest Ethiopia, a study focused on barriers to the uptake of PCC identified some barriers, including poor knowledge, unplanned pregnancies, heavy workloads, service costs, distance, unavailability of services, and insufficient attention from media personnel (19).

~~However, this study addresses the lack of qualitative research on PCC by exploring the experiences, challenges, and opportunities from the perspectives of experts, healthcare providers, and women with a history of pregnancy. We examined current practices and gathered suggestions to enhance PCC awareness and uptake, aiming to inform the implementation of effective interventions.~~Although Ethiopia has strategically integrated PCC into its health system with newly developed guidelines (6, 7), there is limited evidence, particularly on the challenges and opportunities within the system that influence service enhancement. This study addresses this gap by examining experiences, challenges, and opportunities from the perspectives of experts, healthcare providers, and women with pregnancy histories. It evaluates current practices

and provides recommendations to improve PCC awareness and uptake, guiding the development and implementation of effective interventions.

Formatted: Font color: Auto

Materials and methods

Study design: We employed an exploratory qualitative study design to investigate the experience of the current practices, challenges and opportunities for PCC services**.** The study was reported in accordance with the Consolidated Criteria for Reporting Qualitative Research (COREQ) checklist (30).

Study setting

~~Since 2020, Ethiopia has strategically integrated PCC into its health system, guided by newly developed guidelines (6, 7)~~. We conducted the study in the eastern and central regions of Tigray, Northern Ethiopia, ~~which have~~with populations of 994,346 and 1,522,596, respectively. According to the Tigray Regional Health Bureau, in 2020, the region had a total of 14,423,731 healthcare providers (HCPs), including 3,074 health extension workers (HEWs)..These zones include 591,481 women of reproductive age (23.5% of the population) .~~and employ 1,731 healthcare providers, including 350 with Health extension workers (HEWs).~~ The study focused on specific districts and urban areas in both zones, ~~taking place~~ from January 26, 2024, to April 4, 2024. We specifically targeted two rural districts and one urban distrct in the eastern zone, along with two rural districts and one urban woreda in the central zone of Tigray. The region invested in primary health care units, achieving a 91.7% coverage rate. However, the war damaged over 80% of health facilities, leading to a 40% decline in maternal and child health services, including institutional deliveries (26).

~~**Study design:** We employed an exploratory qualitative study design to investigate the experience of the current practices, challenges and opportunities of PCC services provision~~.

~~Recruitment of study~~ ~~P~~participants

~~We included pregnant women and women with potential for future pregnancies in~~In the focus group discussions (FGDs), we included women who have had a history of pregnancy or are currently pregnant and who have the intention to be pregnant. We recruited high-risk mothers (history of APOs like stillbirth, neonatal death, congenital anomalies, perinatal death, miscarriage, post-partum hemmoragge, recurrent abortion (119) or history of chronic medical disease like diabetic mellitus , hypertension or HIV)) for the in-depth interviews (IDIs)~~and high risk mother (history of APOs or chronic medical disease) in the in-depth interviews (IDIs).~~ Using purposive sampling, w~~W~~e identified participants communicated with HEWs and women development group (WDGs) from HEW registers, ~~using purposive sampling,~~ considering their pregnancy and risks. Furthermore, healthcare providers including, HEWs, midwives, nurses, health officers, and medical doctors stationed in Maternal, Newborn, and Child Health (MNCH) units were identified with the guidance of the medical director or MNCH coordinators of these health facilities for IDIs. Additionally, experts from the District Health Office MNCH/HEP (Health extension program) case team in selected districts, the Reproductive maternal neonatal child health (RMNCH) case team from the regional health bureau, and professionals from the Ethiopian Midwives Association and Ethiopian Obstetrics and Gynecology Association participated in the Key informant interviews (KIIs).

Furthermore, we selected healthcare providers for both IDIs and KIIs based on their substantial experience in MNCH and their ability to communicate effectively, thereby providing valuable insights into the current state of PCC.

Data collection~~/ Interviews~~

We obtained participant information through IDIs, KIIs, and FGDs. ~~In order to ~~T~~t~~riangulate and validate the data, we included mothers, medical professionals working in MNCH units, MCNH experts from district and regional health offices, and associations.

Mothers for FGDs and IDIs were identified with the assistance of HEWs and WDGs within the community at their households. After obtaining their consent, we ~~conducted the ~~interviewed in private settings, such as village health posts or households, to ensure privacy and minimize background noise. Medical directors supported~~ the~~ selecti~~o~~ng of healthcare providers for IDIs, who were then interviewed in private rooms at their workplaces. Similarly, we conducted face-to-face interviews ~~for KIIs ~~with MCH experts for KIIs in private rooms at their workplaces. The number of participants was determined based on data saturation, which occurs when participants' descriptions become repetitive. Sampling continued until no new information emerged, indicating ~~that ~~saturation ~~had been~~was reached (27).

Six FGDs were conducted, one in each district, involving 7-9 mothers ~~with the intention ~~to become pregnant, each session lasting 56-96 minutes. Eight mothers with a history of APOs participated in IDIs, averaging 42 minutes per interview. Additionally, 13 ~~healthcare providers~~HCPs from six health centers and health posts were interviewed, averaging 46 minutes each. KIIs involved 10 MNCH experts, ~~including ~~seven from district health offices and RHB, and three clinicians and academics from ESOGA & EMA, with ~~interviews ~~lasting 38-70 minutes of interviews~~.~~

The primary investigator (GG) and three other PhD students in public health (GB, KK, and FT) who are experienced and trained in qualitative research conducted the interviews and FGDs. The four interviewers were paired into two groups, ~~each consisting of ~~one note-taker and one interviewer.

We used interviews and discussion guides to explore experiences, challenges, and opportunities in providing PCC services. We developed semi-structured guides for four groups: mothers with an intention to be pregnant, ~~mothers~~ High-risk mothers~~with a history of APOs~~, ~~healthcare providers~~HCPs ~~-~~working in the MNCH unit, MNCH experts, clinicians and academics in MNCH. These guides were initially drafted in English, translated into the local language, Tigrigna.~~, and pre-tested in a similar setting, with revisions made based on feedback. During data collection, the team held daily debriefing sessions to address emerging issues and spent extended time with participants to gain deeper insights. Preliminary results were presented to experts and peers, leading to further refinement of the guides. While conducting participant interviews, creating codes, and organizing these codes into categories and themes, the researcher team bracketed their prior experiences and knowledge to enhance the quality of the results. To enhance the consistency of the results, we conducted member checking with four participants, each representing a different group: healthcare providers, MNCH experts, and both groups of mothers~~. FGDs were organized in circular seating arrangements to facilitate interactive discussions among participants (28). The interview guides comprised open-ended questions [S1]. Furthermore, the interview guide incorporated probing questions. Interviewers actively guided respondents through the questions outlined in the guide, using probing techniques to prompt further explanation of participants' responses. Throughout the interviews, they audio-recorded all sessions and took field notes to document key points and observe participants' non-verbal cues in-depth.

Trustworthiness

We ensured the reliability of our findings by implementing rigorous quality measures. The data collectors were trained on tools, interview techniques, participant selection, and consent procedures. We pre-tested the topic guide in a similar setting before starting data collection with revisions made based on feedback. During data collection, the team held daily debriefing sessions to address emerging issues and spent extended time with participants to gain deeper insights. We extended the research period to gain an in-depth understanding of the phenomena. We shared participant transcripts for verification and incorporated their feedback. We conducted data collection and analysis simultaneously, triangulating findings from interview transcripts with field notes. Experienced researchers fluent in the local language and culture translated, transcribed, and coded parts of the audio recordings while the primary investigator reviewed their work to ensure accuracy. Preliminary results were presented to experts and peers, leading to further refinement of the guides. While conducting participant interviews, creating codes, and organizing these codes into categories and themes, the researcher team bracketed their prior experiences and knowledge to enhance the quality of the results. To ensure the consistency of our findings, we conducted member checking with four participants, each representing a distinct group: healthcare providers, MNCH experts, and the two groups of mothers. Additionally, we employed IDIs, KIIs, and FGDs as data collection methods to inform and guide subsequent discussions.

Data analysis

Formatted: Line spacing: Double

The FGDs, IDIs and KII were audio recorded, transcribed verbatim in the local language “~~(~~Tigrigna”~~)~~, and ~~then~~ translated into English. Verbatim transcripts of the data saved as an independent ~~file in~~ MS Word file were imported, stored, managed, and coded using Atlas.ti v.7.5.4 (Scientific Software Development GmbH, Berlin,Germany) qualitative data analysis

software. The first author (GG) conducted the initial coding by thoroughly listening to the audio recordings from the FGDs and interviews and carefully reviewing ~~the entire~~all the transcripts to become familiar with the content and to identify initial coding. In addition, we linked field notes and investigator memos to the respective files in the software to assist in the analysis.

The first author (GG) conducted line-by-line coding. We used both process and values coding to analyze ~~the entire~~all the transcripts. Additionally, we applied a hybrid approach, inductive and deductive coding methods during the analysis (29). Another investigator (AM) coded ~~each of~~ the five interviews and the FGDs to check inter-coder reliability. Transcription, translation, analysis, and data collection were conducted simultaneously throughout the data collection period. The investigators grouped similar codes to create categories and subcategories. We reviewed and revised the codes for clarity and consistency then consolidated them to eliminate redundancy and overlap in the analysis. We used a thematic analysis approach to identify the major themes across the categories and subcategories (30). Furthermore, during the write-up, we performed content analysis to describe the frequency of participants in subcategories. Quotes that best described the various categories and frequently expressed sentiments across several groups were chosen and presented in italics.

Ethics approval ~~and consent to participate~~

Formatted: Indent: Left: 0.31", Hanging: 0.5", Line spacing: Double

Formatted: Line spacing: Double

The Institutional Review Board of Mekelle University, College of Health Sciences (reference: MU-IRB2075/2023), granted ethical approval. The Tigray Health Bureau issued a support letter, and the relevant district health offices and villages granted permission. ~~Before data collection, we attached a one-page consent form to the questionnaire, explaining participants' autonomy~~. We

fully explained the study's objectives, risks, and benefits and obtained informed consent from all participants and the consent was approved by the college's institutional review board.. We prioritized privacy and confidentiality and informed participants of their right to withdraw at any time. We also requested permission to record focus group discussions and interviews.

Results

Socio-demographic characteristics of the respondents

In thisThe qualitative study, we included a total ofinvolved 79 participants, including. This comprised 21 individuals in IDIs with , including both healthcare providers (HCPs) and highrisk mothers. –Additionally, 10 MNCH experts from district health offices, regional health bureaus, and professional associations participated in KIIs. Among the high-risk mothers, 63% were aged between 18 and 34 years, all were housewives, and 12.5 % were grand multiparous. Of the healthcare professional participants, 64% were female, with approximately 74% being midwives, public health professionals, or HEWs (Table 1). Additionally, 48 mothers to become pregnant in the future participated in the FGDs. Among these FGD participants, 63% were between 18 and 34 years old, and 46% had an educational level of high school or above (Table 2).with histories of APOs; 48 mothers with pregnancy histories or the intention to become pregnant in FGDs; and 10 MNCH experts from district health offices, regional health bureaus, and associations. Among the mothers, 59% were aged 20–34, and 46% had attended primary school. Of the healthcare professional participants, 64% were female. Approximately, 74% of the HCPs were midwives, public health professionals, and HEW (Table 1).

Table 1: Characteristics of the study participants in Tigray, Northern Ethiopia, 2024

Characteristics	IDIs(N=21)	FGDs(N=6)	KIIs(N=10)

		High risk mothers *(n=8)	HCPs* working in MNCH unit & HEWs*, (n=13)	mothers with intention to become pregnant (n=48)	MNCH* experts from RHB* and district health offices (n=7)	Clinicians & Academics in MCNH from associations(n=3)
Sex	Male		3		3	2
	Female		10		4	1
Age of participants, years	15–19	0	0	2	0	0
	20-34	5	8	28	0	1
	35–49	3	5	18	5	2
	=>50	0	0	0	2	0
Work experience, years	<5 years		2		1	0
	5-10 years		6		2	3
	11-20years		5		4	0
Occupation	House wife	8		42		
	Student	0		1		
	Merchant	0		5		
Educational level	Not attended school	1		3		
	Primary (1-8th	3		23		
	Secondary(9-12th)	4		13		
	Diploma	0	5	8	0	0
	BSc. Degree/MD*	0	8	1	4	1
	MSc. Master /specialty	0	0	0	3	2
Profession	Midwifery		5		0	2

Formatted: Line spacing: Double
Formatted: Line spacing: Double
Formatted: Line spacing: Double
Formatted: Line spacing: Double
Formatted: Line spacing: Double
Formatted: Line spacing: Double
Formatted: Line spacing: Double

	~~Public Health~~		~~1~~		~~5~~	~~0~~
	~~Family health~~		~~0~~		~~2~~	~~0~~
	~~Nursing~~		~~2~~		~~0~~	~~0~~
	~~MD/specialty~~		~~1~~		~~0~~	~~1~~
	~~HEW~~		~~4~~		~~0~~	~~0~~
~~Current working unit~~	~~Clinician & academic~~		~~0~~		~~0~~	~~3~~
	~~MNCH expert~~		~~0~~		~~7~~	~~0~~
	~~MNCH unit~~		~~9~~		~~0~~	~~0~~
	~~HEP*~~		~~4~~		~~0~~	~~0~~
~~Number of pregnancy~~	~~1~~	~~0~~		~~8~~		
	~~2-4~~	~~5~~		~~28~~		
	~~=>5~~	~~3~~		~~12~~		
~~Number of birth~~	~~0-1~~	~~2~~		~~14~~		
	~~2-4~~	~~5~~		~~30~~		
	~~=>5~~	~~1~~		~~4~~		
~~High risk mother~~	~~No~~			~~36~~		
	~~Yes~~			~~12~~		
~~History of PCC* service~~	~~No~~	~~4~~		~~32~~		
	~~Yes~~	~~4~~		~~16~~		

Formatted: Line spacing: Double

Formatted: Line spacing: Double

Formatted: Line spacing: Double

Formatted: Line spacing: Double

Formatted: Line spacing: Double

Table 1: Characteristics of the study participants in Tigray, Northern Ethiopia, 2024

Characteristics		IDIs(N=21)		KIIs(N=10)
		High risk mothers *(n=8)	HCPs (n=13)	HCP Experts*

Sex	Male	NA	3	5
	Female		10	5
Age of participants, years	18-34	5	8	1
	35–49	3	5	7
	=>50	0	0	2
Work experience, years	<5 years	NA	2	1
	5-10 years		6	5
	11-20years		5	4
Occupation	House wife	8	NA	NA*
Educational level	Not attended school	1	NA	NA*
	Primary (1-8th	3		
	Secondary(9-12th)	4		
	Diploma	0	5	0
	BSc. Degree/MD*	0	8	5
	MSc. Master /specialty	0	0	5
Profession	Midwifery	NA*	5	2
	Public Health		1	5
	Family health		0	2
	Nursing		2	0
	MD/specialty		1	1
	HEW		4	0
Current working unit	Clinician & academic	NA*	0	3
	MNCH expert		0	7
	MNCH unit		9	0
	HEP*		4	0

Number of births	0-1	2	NA*	NA*
	2-4	5		
	=>5	1		

NB: PCC, preconception care: High-risk mother: a mother with a history of adverse pregnancy outcomes or chronic medical conditions: HEP, Health extension program: MD, Medical doctor: RHB, Regional health bureau: MNCH, Maternal, Neonatal and child health: HCPs, Healthcare providers working in Maternal, Neonatal, and Child Health related units,: HEWs, Health extension workers : HCP Experts: Healthcare professionals working at woreda health offices, regional health bureaus, and academic institutions

Formatted: Line spacing: Double

Table 2: Characteristics of FGD participants in Tigray, Northern Ethiopia, 2024

Characteristics		(FGD* 1-6)=48 mothers
Age of participants, years	18-34	30
	35–49	18
Occupation	House wife	41
	Student	2
	Merchant	4
	Daily laborer	1
Educational level	Not attended school	3
	Primary (1-8th)	23
	Secondary(9-12th)	13
	Diploma	8
	Degree	1

Number of births	0-1	14
	2-4	30
	=>5	4

NB: FGDs* , Focussed Group Discussion

Themes: Through an analysis of the transcribed interviews, five major themes, encompassing nineteen ~~twenty nine~~ sub-themes, emerged. Awareness and experience, current practice of PCC, challenges, opportunities, and suggestions themes have emerged from the qualitative data on PCC services provision (Table 2).

Table 2. A list of t~~T~~hemes and s~~S~~ub-themes e~~E~~merged from the d~~D~~ata, Tigray, Northern Ethiopia,2024

Major themes	Sub-themes
Awareness of PCC services	Information
	Perceived benefits
Experiences of PCC services	Practices of PCC interventions
	Home-based preparation
Challenges of PCC services	Fragmented services
	Traditional beliefs and misconceptions
	Poor quality of counseling on contraceptive services
	Social influences
	High Workload
	Services cost
	Perceived lack of medicines and medical equipment

Formatted: Line spacing: Double

Formatted: Font: 11 pt, Bold

Opportunities of PCC services	Existing community platforms
	Perceived desire of PCC services
Perceived suggestions for PCC services	Home-based initiative
	Use a variety of communication strategies
	Use high-risk mothers as role models
	Community engagement activities
	Community -health facility linkage
	Services integration
Major themes	Sub-themes
Awareness and experience of PCC services	Home-based preparation
	Awareness
	Perceived benefit of PCC
	Knowledge
	Components of PCC services
Challenges of PCC services	Poor awareness
	Deliver in a fragmented
	Belief, and misconceptions
	Unintended pregnancy
	Lack of perceived desire to disclose conception
	Influence of elder person and religious leaders
	Poorly perceived of services for low risk women
	Husband opposition PCC
	Workload

Formatted: Line spacing: Double

Formatted: Line spacing: Double

Formatted: Line spacing: Double

	Services cost
	Scarcity of resources
	Poor quality of family planning services
Opportunities for improving of awareness and uptake of PCC	Smart start model
	Utilize the existing community platforms
	Integrating the services into existing health facilities
	Perceived interest of HCP in PCC services
	Maternal interest in PCC services
Suggestions for increasing PCC awareness and uptake	Home-based initiative
	Use variety communication strategies
	Use high-risk mothers as role model for education
	Community based PCC services
	Linkage
	PCC services would be free of charge
	Need focus from government
	Participants' view on PCC services

Formatted: Line spacing: Double

Formatted: Line spacing: Double

Formatted: Line spacing: Double

The conceptual framework, emerged from the qualitative data, illustrates the overall relationships among the factors involved in PCC service. This framework helps in the implementation of these services within primary healthcare units. The findings derived from the qualitative data highlight the overall relationships between the sub-themes, themes, and their connection to PCC services. A higher level of awareness and positive experiences positively impact PCC services. Conversely, challenges such as traditional beliefs, misconceptions, and fragmented PCC

services negatively affect PCC services, including the level of awareness and experiences. This framework helps in the implementation of these services within primary healthcare units. (Fig 1).

Formatted: Font: 14 pt

Figure 1: Framework of Experiences, Challenges, and Opportunities in PCC Services in Tigray, Ethiopia (Developed by Researchers) **Note:** The dashed lines represent the negative influence on PCC services, while the solid lines indicate a positive influence.

Awareness and experience of PCC services

Home-based preparation: Adopting a pregnancy plan allows women to adequately prepare for conception. Preparedness for pregnancy is crucial to minimize modifiable risk behaviors that can lead to poor pregnancy outcomes. This proactive approach not only encourages planned, healthy pregnancies but also fosters positive birth outcomes and overall well-being for both women and infants. Some mothers, particularly high risk mothers, take proactive steps to prepare for pregnancy. They save money for medications, food, and childcare, and focus on mental preparation to ensure stability when facing the challenges of pregnancy, especially during the initial trimester.

A mother with a history of APOs said: *"before the mother becomes a pregnant, need to get prepared psychologically and economically. Psychologically prepared means mentally she needs to be stable, she need to eat more than usual (4-5 times per day)"* ***(29 years old mother, 10th grade, IDI)***

A mother mentioned that when planning for pregnancy, she prepares the chicken with local alcohol (siwa) for her husband and drinks " siwa siray " herself, believing it benefits the child.

……."a chicken should be slaughtered and Siwa (local alcohol) should be prepared, it is done

before you get pregnant or when you are thinking about pregnancy. It is mostly for my husband but myself also drinks the siwa siray *and it is good for the child"* ***(35 years old mother, not attended school, FGD)***

Pre-pregnancy information was not adequately emphasized in healthcare services, including those within the community, leading to a lack of essential information for women to properly prepare for pregnancy. Participants emphasized that most mothers, particularly those without known health issues, do not take any action or invest in pregnancy preparation. Mothers with a history of APOs stated:

……"I didn't plan ahead to get pregnant." Some mothers are unaware of the kind of care or preparation that should be done prior to becoming pregnant. In my situation, I did not take any extra precautions prior to being pregnant, so I continued to eat normally and did not seek medical advice or counsel on PCC until after I became pregnant. ***(29 years old mother, 10th grade, IDI)***

1. Awareness of PCC services

Information of PCC: Access to information about a service is essential for utilizing it effectively. Awareness of a service is crucial for its utilization. Without awareness of its existence ,knowing it exists, people cannot benefit from it. For many women and community members, the concept of PCC remains unfamiliar, as it is a relatively new approach that the healthcare system has not actively promoted. Both mothers and HCPs indicated that most women lack information about PCC, turning to healthcare services only after pregnancy confirmation or fertility difficulties. This gap in understanding repeatedly frequently results in confusion between PCC and prenatal care. High High-risk mothers statedpeople can't use it. According to the participants, the majority of women was unaware of PCC and only sought health services after confirming pregnancy or facing conception issues.

Formatted: Font: (Default) Times New Roman, 12 pt, Bold, Font color: Text 1

Formatted: Font: (Default) Times New Roman, 12 pt, Bold, Font color: Text 1

Formatted: List Paragraph, Line spacing: Double, Numbered + Level: 1 + Numbering Style: 1, 2, 3, … + Start at: 1 + Alignment: Left + Aligned at: 0.25" + Indent at:

Formatted: Font: (Default) Times New Roman, Bold, Font color: Text 1

Formatted: Line spacing: Double

This lack of awareness led to confusion between PCC and prenatal care. Mothers with a history of APOs said:

….."I do not know what pre-pregnancy care is?" ***(32 years old mother, 5th grade, IDI)***

."Endie….the meaning of this local language is ***(****I don't know***)****. I am just keeping silent"* ***(28 years old mother, 8th grade, IDI)***

.

Similarly, a Clinical midwifery professional said,

"…..there is a lack of information regarding preconception in the community; I couldn't expect mothers to visit and utilize preconception care. I believe that we healthcare providers and health professional associations didn't invest in it, and from my observation in our context, I can conclude that no mothers are coming to preconception care, even though it is not supported by research" ***(Midwifery professional, male, KII)***.

Formatted: Font: Not Italic

On the Other hand, participants noted that some high-risk mothers have information about the services. They emphasized that healthcare providers should advise these mothers, including those with a history of diabetes, hypertension, HIV, spontaneous abortion, infertility, or congenital abnormalities, to undergo various health screenings before conception. These screenings should include tests for non-communicable and communicable diseases, sexually transmitted infections, mental health issues, gender-based violence, and other chronic conditions. Additionally, they should receive advice on proper nutrition and avoid harmful substances like alcohol. A high-risk mother said,

I have heard information on PCC from media outlets such as Tigrai Television, in a program called "Maeda Hakaym." I think it is good and beneficial to have a planned life and children. It sounds nice to have plan, preparations, and examinations and give birth based on planned

decisions. Screening on gender-based violence, chronic disease, sexually transmitted infections, substance use, mental health, nutritional counseling and balanced diet and consultation to a doctor in the presence of these factors and medication use is essential to prevent adverse pregnancy outcome" ***(35 years old mother, 10th grade, IDI)***.

Even some mothers explain the concept of PCC and understand its benefits.

"I understand that PCC means I need to check my health status, should not take alcoholic drinks, need to have mental stability, and limit the number of children that I have according to my income or relation to livelihood. These come to my mind when I am thinking about PCC" ***(29 years old mother, 10th grade, IDI)***

Besides, most HCPs have some knowledge of PCC and emphasize the importance of women's health before conception. Gynecology professionals, medical doctors, and, to a lesser extent, midwifery professionals have a better understanding of PCC concepts and interventions. Key PCC services identified by HCPs include HIV testing, folic acid supplementation, nutritional education, family planning, chronic disease screening, substance use advice, STI/HIV screening, medication management, and assessment of APOs such as Rh incompatibility, Td vaccination, abortion, and congenital anomalies. Among these, folic acid supplementation, nutritional advice, and family planning were most frequently mentioned.

HIV testing, folic acid supplementations, feeding practices, and nutrition are the things I know about preconception care services ***(Midwifery professional, female, IDI)***

All participants concurred that nearly all healthcare providers are knowledgeable about recommending three months of folic acid supplementation to mothers with a history of congenital anomalies and repeated abortions before conception.

Formatted: Justified

Formatted: Font: (Default) Times New Roman, 12 pt

Formatted: Font: Bold

Mothers who had a history of repeated abortion before conception are eligible to take folic acid for three months. We don't give folic acid supplementation to all eligible women ***(HEW, female, IDI)***

On the other hand, participants reported that some HCPs have no information about PCC and do not even recognize its name.

.... No, I don't know. I don't even think there is such care here. It has been a long time since I started working here; I never heard anyone talking about it, and I never saw such service being provided here (**Nursing professional, female, IDI)**

We don't even know about preconception care ourselves. We health professionals don't know it ***(HEW, female, IDI)***

~~Participants noted that awareness varies, but it is relatively higher among mothers with a history of APOs. They emphasized that healthcare providers should advise these mothers, including those with a history of diabetes, hypertension, HIV, spontaneous abortion, infertility, or congenital abnormalities, to undergo various health screenings before conception. These screenings should include tests for non-communicable and communicable diseases, sexually transmitted infections, mental health issues, gender-based violence, and other chronic conditions. Additionally, they should receive advice on proper nutrition and avoid harmful substances like alcohol. Some mothers even explained the concept of PCC and understood its benefits.~~

~~*"Screening for communicable disease (STIs, HIV, Hepatitis b, and malaria), none communicable disease (diabetes, hypertension) and other health problems should be done for a mother at preconception period to know her status"* **(32 years old mother, 5th grade, IDI)**~~

~~*........" So, the woman should take adequate nutrition before and during pregnancy to give birth to a healthy baby"* **(35 years old mother, not attended school, IDI)**~~

Formatted: Font: (Default) Calibri, 11 pt

Formatted: Left

Formatted: Font: Italic

Formatted: Line spacing: Double

Even some mothers explain the concept of PCC and understand its benefits.

I understand that PCC means, I need to check my health status, should not take alcohol drinks, need to have mental stability and limit the number of children that I have according to mu income or relation to livelihood. These come to my mind when I am thinking about PPC ***(29 years old mother, 10th grade, IDI)***

I have ever heard information on PCC from media outlets such as Tigrai Television, in a program known as "Maeda Hakaym." I think it is good and beneficial to have a planned life and children. It sounds nice to be able to have plan, preparations, and examinations and give birth based on make planned decisions. Screening on gender-based violence, chronic disease, sexually transmitted infections, substance use, mental health, nutritional counseling and balanced diet and consultation to a doctor in the presence of these factors and medication use is important to prevent adverse pregnancy outcome" ***(35 years old mother, 10 th grade, IDI)***.

Perceived benefit of PCC services

Mothers and HCPs emphasized that PCC offers valuable opportunities to assess women's health, enabling early identification and management of potential issues. This approach not only enhances maternal healthcare services but also helps avoid unnecessary expenses and reduces the risk of mother-to-child HIV transmission.

Give opportunities for screening of health status: PCC provides opportunities to assess the health status of mothers, enabling early detection of various conditions and diseases and identifying potential preconception risks that may lead to adverse pregnancy outcomes, as noted by participants. This aspect contributes to the promotion of women's health.

"Preconception care plays a crucial role in screening for various health risks such as substance

Formatted: Justified

use, gender-based violence, mental illness, HIV/STIs, and chronic conditions like diabetes, hypertension, and heart disease before conception occurs" ***(29 years old mother, 10th grade, IDI)***

Formatted: Font: (Default) Times New Roman, 12 pt, Italic

Enhance maternal healthcare services: Some participants noted that PCC services would facilitate antenatal, delivery, and postnatal care services.

……."Moreover, providing expectant mothers with guidance on the timing of childbirth, pre-pregnancy preparations, pregnancy care, and postnatal support can significantly ease their journey. As emphasized earlier" ***(Public health professional, male, KII)***

Formatted: Justified, No widow/orphan control, Don't adjust space between Latin and Asian text, Don't adjust space between Asian text and numbers

Formatted: Font:

"If we work on preconception care earlier now, they will be able to start ANC contact on their day" ***(Public health professional, female, KII)***

Prevent HIV transmission from mother to child: The optimal time to prevent HIV transmission from mother to child is during the pre-pregnancy period, as identified by participants.

……"It is essential to undergo various tests, such as HIV testing, before pregnancy to prevent the transmission of the virus to the unborn child" ***(24 years old mother, 10th grade, FGD)***

It (PCC) can help to prevent mother-to-child transmission of diseases ***(30 years old mother, diploma, FGD)***

Formatted: Font: (Default) Arial, 12 pt

Save unnecessary expenses and efforts: Participants pointed out that some women spend their money and energy on ultrasounds and other laboratory tests privately during pregnancy because they did not consult healthcare providers before becoming pregnant. However, if these tests were initiated earlier, mothers could avoid unnecessary expenses and efforts.

~~Perceived benefit of PCC~~

Formatted: Line spacing: Double

Participants emphasized that PCC offers valuable opportunities to assess women's health, enabling early identification and management of potential issues. This approach not only enhances maternal healthcare services but also helps avoid unnecessary expenses and reduces the risk of mother-to-child HIV transmission.

Give opportunities for screening of health status: PCC provides opportunities to assess the health status of mothers, enabling early detection of various conditions and diseases, and identifying potential preconceptional risks that may lead to adverse pregnancy outcomes, as noted by participants. This aspect contributes to the promotion of women's health.

"Preconception care plays a crucial role in screening for various health risks such as substance use, gender-based violence, mental illness, HIV/STIs, and chronic conditions like diabetes, hypertension, and heart disease before conception occurs" ***(29 years old mother, 10th grade, IDI)***

Enhance maternal healthcare services: Some participants noted that PCC services would facilitate antenatal, delivery, and postnatal care services.

……."Moreover, providing expectant mothers with guidance on the timing of childbirth, pre-pregnancy preparations, pregnancy care, and postnatal support can significantly ease their journey. As emphasized earlier" ***(Public health professional, male, KII)***

"If we work on preconception care earlier now, they will be able to start ANC contact on their day" ***(Public health professional, female, KII)***

Prevent HIV transmission from mother to child: The optimal time to prevent HIV transmission from mother to child is during the pre-pregnancy period, as identified by

~~participants.~~

~~……"*It is important to undergo various tests, such as HIV testing, before pregnancy to prevent the transmission of the virus to the unborn child*" ***(24 years old mother, 10th grade, FGD)***~~

~~*It (PCC) can help to prevent mother to child transmission of diseases* ***(30 years old mother, diploma, FGD)***~~

~~**Save unnecessary expenses and efforts:** Participants pointed out that some women spend their money and energy on ultrasounds and other laboratory tests privately during pregnancy because they did not consult healthcare providers before becoming pregnant. However, if these tests were initiated earlier, mothers could avoid many unnecessary expenses and efforts.~~

2. Experience with PCC services

Current Practices in the Components of PCC Interventions

~~Current Practices of Healthcare Providers Regarding Components of PCC interventions~~

Formatted: Line spacing: Double

~~**Knowledge of PCC:** Most HCPs have some knowledge of PCC and emphasize the importance of women's health before conception. Gynecology professionals, medical doctors, and, to a lesser extent, midwifery professionals, have a better understanding of PCC concepts and interventions. Key PCC services identified by HCPs include HIV testing, folic acid supplementation, nutritional education, family planning, chronic disease screening, substance use advice, STI/HIV screening, medication management, and assessment of APOs such as Rh incompatibility, Td vaccination, abortion, and congenital anomalies. Among these, folic acid supplementation, nutritional advice, and family planning were most frequently mentioned. Although there is no formal PCC program, healthcare workers highlight adolescent health programs, premarital HIV~~

testing, and family planning services as essential components related to PCC interventions.

"I have information about how to lose weight (weight loss) through health education, nutritional supplementation, smoking should be reduced, opioid misuse of drugs should be reduced, alcohol should be reduced, and immunizations (vaccination) should be administered before pregnancy" ***(General practitioner professional, male, IDI)***

HIV testing, folic acid supplementations, feeding practices, or nutrition are the things I know about preconception care services ***(Midwifery professional, female, IDI)***

All participants concurred that nearly all healthcare providers are knowledgeable about recommending three months of folic acid supplementation to mothers with a history of congenital anomalies and repeated abortions prior to conception.

Mothers who had history of repeated abortion before conception are eligible to take folic acid for three months. We don't give folic acid supplementation to all eligible women ***(HEW, female, IDI)***

On the other hand, participants reported that majority HCPs have no information about PCC and do not even recognize its name.

……. No, I don't know. I don't even think there is such care here. It has been a long time since I started working here; I never heard anyone talking about it, and I never saw such service being provided here (**Nursing professional, female, IDI)**

……"It is a new concept for the community and most health professionals including HEWs do not know exactly what preconception care is?" ***(Family health professional, female, KII)***

We don't even know about preconception care ourselves. We health professionals don’t know it (***HEW, female, IDI)***

Current practices on Components of PCC interventions: Participants observed that while "PCC" is not widely recognized, providers still practice components like family planning, iron and folic acid supplementation, and premarital HIV testing, often unintentionally. They agreed that primary healthcare settings lack routine PCC services with assigned providers or designated units, missing a significant opportunity. HCPs highlighted a significant gap in healthcare settings, attributing it to the absence of routine PCC services with designated providers or specialized units, leading to missed opportunities for comprehensive care. They observed that They also noted that providers deliver some PCC interventions, such as folic acid supplementation and various forms of counseling, in a fragmented manner across various units like including antenatal care, gynecology/obstetrics, youth-friendly service units, family planning, chronic disease clinics, and post-abortion care, especiallyparticularly for high-risk mothers., aiming to reduce APOs. Although Mmidwives and gynecologists deliver provide some of these services, but participants highlighted that private clinics offer them more commonly provide themconsistently.

A public health professional discussed how various units provide components of PCC interventions as: *“It is done irregularly in youth friendly services unit like the family planning itself, reproductive and diet education. But it is not deep. But something is tried. Usually, as I tell you, the peer to peer takes the biggest share”* ***(Public health professional, male, KII)***

.....in addition to these mothers diagnosed with DM, threatened abortion, spontaneous abortion, recurrent still birth etc are informed and appointed to consult healthcare provider before getting

Formatted: Space After: 0 pt, Line spacing: Double, No widow/orphan control, Don't adjust space between Latin and Asian text, Don't adjust space between Asian text and numbers

Formatted: Line spacing: Double

pregnancy in gyn/obs OPD. The consultation is specifically regarding folic acid, there is no more service provided for other possible reasons ~~**(Clinical**~~ ***M***~~***m***~~***idwifery professional, male, KII***

~~A 35 years old women said:~~ ~~*As a result of my personal health issues, I sought advice from healthcare providers. I diagnosed for hypertension, which unfortunately led to a stillbirth in the past. Therefore, when I planned my next pregnancy, I consulted with health workers to ensure the best possible outcome. I even visited referral hospitals for further guidance and was advised to visit healthcare facilities ahead of any future pregnancy. I followed this advice accordingly*~~ ~~***(35 years old mother, 9th grade, FGD)***~~

~~Contrarily, an MCH expert employed at the district health office provided insight into the availability of PCC services within healthcare facilities as follows:~~

~~*In both urban and rural locations, pre-pregnancy care is currently nonexistent. It hasn't existed before, as far as I can tell. Policy, too, begins with family planning. Additionally, access to preconception care is not as high as indicated. I won't be questioned about whether or not I offer the services. I'm speaking to you based on my level of expertise. It was lately that I attended training. I received thorough training on PCC in **Adama**, and it isn't offered locally **(Public health professional, male, KII)***~~

~~Similarly, women have not received any counseling as pre-pregnancy care, even for those women who have visited the health facility for the removal of their contraceptives for the purpose of pregnancy. Mothers with history of APOs said:~~

~~*……"For instance, before my fifth pregnancy, I used a method of birth control to avoid getting pregnant for eight years. When we decided to have a child for a variety of reasons, I went to a health facility and asked the HCP about our plans and wanted to have the implant contraceptive medication*~~

*removed in accordance with our plan. After removing the contraceptive, I got pregnant. HCPs did not educate us about PCC or other preconception care at that time" **(32 years old mother, 5th grade, IDI)***

A public health professional discussed how various units provide components of PCC interventions as: *"It is done irregularly in youth friendly services unit like the family planning itself, reproductive and diet education. But it is not deep. But something is tried. Usually, as I tell you, the peer to peer takes the biggest share"* ***(Public health professional, male, KII)***

.....in addition to these mothers diagnosed with DM, threatened abortion, spontaneous abortion, recurrent still birth etc are informed and appointed to consult healthcare provider before getting pregnancy in gyn/obs OPD. The consultation is specifically regarding folic acid, there is no more service provided for other possible reasons ***(Clinical midwifery professional, male, KII)***

On the other handin general,, the majority of participants noted that the components of PCC interventions provided mainly for the high risk women are:

Contraceptive counseling for pregnancy delay: All participants, including both users and providers, noted that contraceptive counseling for delaying pregnancy was offered to all women of reproductive age within the community and healthcare system.

Provide folic acid supplementation: All participants, both users and providers, agreed that folic acid supplementation was commonly provided to mothers with a history of chronic diseases such as diabetes, congenital anomalies, and spontaneous abortion, making it one of the most frequently practiced components of PCC interventions.

……"We provide folic acid for women who have history of abnormal pregnancy like spinal

bifida, hydrocephalus, and anencephaly. We give a priority for these women” ***(MD,~~Obstetrics and gynecology professional,~~ male, KII)***

Counseling about medication safety: Participants mentioned that some women with a history of chronic diseases such as diabetes and hypertension received counseling about medication safety before conception when they visited the health facility for follow-up.

“ *Need advice about medication with alcohol, if mothers have diabetic mellitus, HIV, and Hypertension should advise her that the medicines should be changed first before pregnant for example ACE inhibitors drugs have teratogenic effect*” ***(~~General practitioner professional~~MD, male, IDI)***

Counseling about substance use: Some mothers with a history of APOs, including abortion, chronic disease, congenital anomalies, and alcohol intake, received suboptimal counseling. They were advised to avoid medications that cause teratogenic effects and to abstain from substance use. Additionally, they were recommended to take folic acid supplements and undergo HIV testing before conception.

Women counsel to take folic acid before 3 months and to avoid bad habits such as alcohol, smoking, and other addictive substances ***(Family health professional, female, KII)***

Contrarily, an MCH expert employed at the district health office provided insight into the availability of PCC services within healthcare facilities as follows:

In both urban and rural locations, pre-pregnancy care is currently nonexistent. It hasn't existed before, as far as I can tell. Policy, too, begins with family planning. Additionally, access to preconception care is not as high as indicated. I won't be questioned about whether or not I offer

the services. I'm speaking to you based on my level of expertise. It was lately that I attended training. I received thorough training on PCC in Ethiopia xxx town, and it isn't offered locally ***(Public health professional, male, KII)***

Moreover, most of the mothers agreed that women seek pre-pregnancy care only when concerned about their health. A high-risk mother noted:

As a result of my personal health issues, I sought advice from healthcare providers. I was diagnosed with hypertension, which unfortunately led to a stillbirth in the past. Therefore, I consulted with health workers to ensure the best possible outcome when I planned my next pregnancy. I even visited referral hospitals for further guidance and was advised to visit healthcare facilities ahead of any future pregnancy. I followed this advice accordingly ***(35 years old mother, 9th grade, FGD****)*

Additionally, mothers reported not receiving any counseling as part of pre-pregnancy care, even those who visited health facilities to have their contraceptives removed in preparation for pregnancy.

"When you go to health facilities for contraceptive removal, the care providers will ask you "Is it because you want to conceive? " Then, they would simply remove you and do nothing else, even if you said yes. There is no preconception care, there is no counseling, and they didn't assess your eligibility for pregnancy. Due to this, the community doesn't have the awareness, doesn't know what PCC is or what to do, and as a result, there is no demand for the service." ***(35 years old mother, 10th grade, IDI)***

Home-based preparation: Adopting a pregnancy plan allows women to adequately prepare for

Formatted: Line spacing: Double

conception. Preparedness for pregnancy is crucial to minimize modifiable risk behaviors that can lead to poor pregnancy outcomes. This proactive approach not only encourages planned, healthy pregnancies but also fosters positive birth outcomes and overall well-being for both women and infants. Some mothers, particularly high risk mothers, take proactive at home such as saving for medications, modifying alcohol intake, improving their diet, and focusing on mental readiness.steps to prepare for pregnancy. They save money for medications, food, and childcare, and focus on mental preparation to ensure stability when facing the challenges of pregnancy, especially during the initial trimester.

A mother with a history of APOs said: *"before the mother becomes a pregnant, need to get prepared psychologically and economically. Psychologically prepared means mentally she needs to be stable, she need to eat more than usual (4-5 times per day)"* ***(29 years old mother, 10th grade, IDI)***

A mother mentioned that when planning for pregnancy, she prepares the chicken with local alcohol (siwa) for her husband and drinks " siwa siray " herself, believing it benefits the child.

……."a chicken should be slaughtered and Siwa (local alcohol) should be prepared, it is done before you get pregnant or when you are thinking about pregnancy. It is mostly for my husband but myself also drinks the siwa siray *and it is good for the child"* ***(35 years old mother, not attended school, FGD)***

Pre-pregnancy information was not adequately emphasized in healthcare services, including those within the community, leading to a lack of essential information for women to properly prepare for pregnancy. On the contrary, pParticipants emphasized that most mothers, particularly

those without known health issues, do not take any action or invest in pregnancy preparation. A high risk ~~M~~mother~~s~~ with a history of APOs stated:

……"I didn't plan ahead to get pregnant." Some mothers are unaware of the kind of care or preparation that should be done prior to becoming pregnant. In my situation, I did not take any extra precautions prior to being pregnant, so I continued to eat normally and did not seek medical advice or counsel on PCC until after I became pregnant. ***(29 years old mother, 10th grade, IDI)***

~~2.~~ 3. Challenges of ~~preconception care~~PCC services

Formatted: Normal, Indent: Left: 0.25", Line spacing: Double, No bullets or numbering

Formatted: Font: (Default) Times New Roman, 14 pt, Bold, Font color: Text 1

Formatted: Font: (Default) Times New Roman, Bold, Font color: Text 1

Formatted: Line spacing: Double

~~**Poor awareness of PCC**~~

~~**The community lacks information about pre-pregnancy care:** Most women and community members are unfamiliar with PCC, as it is a new concept that the healthcare system does not promote. Health professionals view it as an additional benefit rather than an essential one. Consequently, women typically visit healthcare facilities only after confirming pregnancy or encountering conception issues, resulting in poor awareness and uptake of PCC.~~

~~*"…..there is a lack of information regarding preconception in the community, I couldn't expect mothers to visit and utilize preconception care. I believe that we healthcare providers and health professional associations didn't invest in it, and from my observation in our context, I can conclude that there are no mothers coming to preconception care, even though it is not supported by research"* ***(Clinical midwifery professional, male, KII)***~~

~~Deliver fragmented~~Fragmented~~- services~~based services: Participants observed that ~~healthcare providers~~ HCPs deliver fragmented PCC components to high-risk women, resulting in low awareness and uptake. Key challenges include the lack of ~~package-based services and access to~~

PCC guidelines, limited government focus, and inadequate training~~, and insufficient provider awareness.~~

"In general, the health system gave no focus to PCC. In practice, no structures have been established in ~~any~~ healthcare institutions to support PCC. I think this is the main challenge. Hence, if there is no center for PCC in the health system, if there is no focus on PCC, and if the health care provider ~~will not~~does not ~~give emphasis~~ emphasize to PCC, the community will do so. ~~Moreover, healthcare providers are not receiving adequate training in PCC, leading to poor attitudes and a lack of skill in this area.~~

I believe this is the primary challenge" ***(~~Obstetrics and gynecology professional,~~MD, male, KII)***

In general, all participants noted that while limited orientation or information about PCC was provided, the government prioritized and delivered package-based services such as antenatal care (ANC) and delivery services.

"A kind of orientation or information was given about PCC by partners, but they still do not get that much focus from the government or partners on PCC like the other health care services. Through education, the community shows behavioral change about child and maternal nutrition" ***(Family health professional, female, KII)***

Traditional ~~B~~belief and misconceptions

Formatted: Line spacing: Double

Lack of felt need to disclose desire to conception: Mothers and healthcare providers have noted that, with few exceptions, most women tend to keep their desire to have a child confidential. Many women even conceal their pregnancies during the early months. They typically do not discuss their intentions to conceive with others unless it is with their husband, a very close friend. While discussing pre-pregnancy care may seem taboo or embarrassing within certain segments of the community, it might be disclosed after pregnancy is confirmed because

pregnancy is perceived as a gift from St. Mary or God.

~~Mothers~~High-risk Mother ~~with a history of APOs~~ said:

………" Discussing care before pregnancy with a neighbor or a part of the community is often considered impolite or embarrassing. Instead, it's more common to share this information once pregnancy is officially confirmed, as there's a belief that pregnancy is a blessing from St. Merry or God ***(32 years old mother, 5th grade, IDI***)

"Mostly, it is in the latter stages of pregnancy that the woman discloses her pregnancy status; except for a for a few, most of the women keep it confidential" ***(29 years old mother, 10th grade, IDI***)

Additionally, a MCH expert said: *"Disclosing or consulting with healthcare providers before getting pregnant is the biggest challenge in the community. Mothers may feel ashamed to consult; normally, it shouldn't be* "***(~~Reproductive~~Public health professional, female, KII)***.

Misconception about fear of side effects: Mothers noted that contraception, especially the Depo injection, is believed to cause infertility. Women avoid using Depo because it delays pregnancy after discontinuation. Due to this misconception, many women are discouraged or restricted from using contraceptives.

"Now, for example, faith in the contraceptive (depo) causes a delay of pregnancy and hence many mothers do not use contraception" ***(~~General practitioner professional~~MD, male, IDI)***

A maternal health expert pointed out that mothers discontinue contraceptives because they believe these may be responsible for the increase in congenital anomalies.

……"You see, especially now, after encountering many congenital anomalies, numerous mothers

have approached us to discontinue family planning" **(Midwifery professional, female, IDI)**

Beliefs about conception: participants highlighted an entrenched cultural belief within the community that could impede women from accessing PCC services. This belief revolves around the perception that pregnancy is not solely determined by individual choices but is instead viewed as a divine gift from God or influenced by St. Mary.

……… According to the moms' and society's beliefs, conception is a gift from God or determined by St. Merry, and being pregnant or the moment a mother becomes pregnant is not something a mother chooses to have. ***(Family health professional, female, KII)***

………"When you think of pre-pregnancy testing, you might wonder if God doesn't do it. The question may arise as to how you come by the work of God. It is God's work whether a child is created or not" ***(HEW, female, IDI)***

Misconception about the risks of pregnancy loses and mistrust of providers: Participants noted that mothers often mistakenly believe the TT vaccine during pregnancy may cause pregnancy loss. They also think healthcare providers don't fully understand the natural or genetic causes of recurrent loss, which continues despite treatment, potentially affecting the acceptance of PCC. A mother with history of APOs said;

"Maybe it is a natural problem. I may naturally have a problem. I have abdominal bloating; I used to feel as if something was moving inside my abdomen. I don't know about the causes of the others, but mine might be associated with Mengegna. Mengegna meant something natural, an inborn problem that causes pregnancy loss. Even the doctors didn't know it; they give you medicines, but they don't tell you what the problem is. For instance, in the most recent loss, the doctor told me to take the medicine because I had recurrent pregnancy loss; however, he didn't tell me what exactly the problem behind this recurrent loss was" ***(28 years old mother, 8th***

~~***grade, IDI)***~~

~~Myth about contraception: Some mothers also mentioned that rumors spread by religious leaders, who claim that contraception is a sin, further complicate the situation. This belief may be an obstacle for pre-pregnancy care because, for PCC, women need to have a plan for pregnancy; otherwise, it will be difficult to provide the services.~~

~~*The rumors spread by religious leaders who claim contraception are a sin further complicates the situation* ***(Nursing professional, female, IDI)***~~

~~**Misconception about fear of side effects**: Mothers noted that contraception, especially the Depo injection, is believed to cause infertility. Women avoid using Depo because it delays pregnancy after discontinuation. Due to this misconception, many women are discouraged or restricted from using contraceptives.~~

~~*"Now, for example, faith in the contraceptive (depo) causes a delay of pregnancy and hence many mothers do not use contraception"* ***(General practitioner professional, male, IDI)***~~

~~A maternal health expert pointed out that mothers discontinue contraceptives because they believe these may be responsible for the increase in congenital anomalies.~~

~~*……"You see, especially now, after encountering many congenital anomalies, numerous mothers have approached us to discontinue family planning"* ***(Midwifery professional, female, IDI)***~~

Misconception about intake of alcohol: Participants noted that consuming traditional alcohol ("Siwa" and "Myes") is not harmful except when taken with medication, particularly during the first four months of pregnancy. Additionally, some mothers believe that alcohol does not increase the risk of abortion or stillbirth; instead, they attribute these risks to poor nutrition, lack of adequate rest, and stress. Participants also mentioned that some elders believe consuming

honey before pregnancy may facilitate the mental development and good health of the fetus later during pregnancy.

"There is no problem with the intake of alcohol before pregnancy. Even though it does not cause any harm until four months of pregnancy, according to the lesson we took, Drinking alcohol is not allowed when you are taking medication. However, it has no problem with other issues, including. Drinking milk is not allowed after six months of pregnancy" ***(35 years old mother, not attended school, IDI****)*

"Yes, for instance, our elders say it is good to take honey before pregnancy. They say it facilitates mental development and the good health of the foetus later during pregnancy. Also recommended by the community is Myes intake. I don't think these substances can cause abortion or stillbirth. Abortion and stillbirth are mainly caused by the factors I mentioned earlier, such as poor nutrition, a lack of adequate rest, and stress" ***(35 years old mother, 10th grade, IDI.***

Poor quality of counseling on contraceptive services

~~**Unwanted pregnancies due to concerns about side effects**~~

Formatted: Line spacing: Double

~~Both~~ M~~m~~others and ~~healthcare providers~~HCPs acknowledged that many women are deterred from using contraceptives due to fears of side effects such as delayed pregnancy and bleeding. Discontinuing contraceptives, switching to alternatives, or stopping altogether can lead to unexpected pregnancies, which hinder the provision of PCC services.

"I was on birth control ~~shots~~ for four years but stopped because I experienced intense bleeding or a hemorrhagic condition, which led to my pregnancy. It wasn't something I had planned for. I only used contraceptives for the first pregnancy (since it was my first, I didn't use them again), but all my other pregnancies were unplanned. They happened after I stopped using birth control

due to negative side effects. I attempted to use birth control again but stopped due to these side effects and tried the calendar method, but that didn't work, and I ended up pregnant again. I didn't have a specific plan for my reproductive life. A lot of it just happened by chance, not by design." ***(35 years old mother, 10th grade, IDI)***

~~**Lack of felt need to disclose desire to conception:** Mothers and healthcare providers have noted that, with few exceptions, most women tend to keep their desire to have a child confidential. Many women even conceal their pregnancies during the early months. They typically do not discuss their intentions to conceive with others unless it is with their husband, a very close friend. While discussing pre-pregnancy care may seem taboo or embarrassing within certain segments of the community, it might be disclosed after pregnancy is confirmed because pregnancy is perceived as a gift from St. Mary or God.~~

~~Mothers with a history of APOs said:~~

~~*………" Discussing care before pregnancy with a neighbor or a part of the community is often considered impolite or embarrassing. Instead, it's more common to share this information once pregnancy is officially confirmed, as there's a belief that pregnancy is a blessing from St. Merry or God* ***(32 years old mother, 5th grade, IDI)***~~

~~*"Mostly, it is in the latter stages of pregnancy that the woman discloses her pregnancy status; except for a for a few, most of the women keep it confidential"* ***(29 years old mother, 10th grade, IDI)***~~

~~Additionally, a MCH expert said: *"Disclosing or consulting with healthcare providers before getting pregnant is the biggest challenge in the community. Mothers may feel ashamed to*~~

~~*consult; normally, it shouldn't be "***(Reproductive health professional, female, KII)***~~

Social influences

~~**Influence of elder person and religious leaders**~~

Formatted: Line spacing: Double

~~Resistance to planning pregnancy:~~ Participants highlighted the influential role of husbands, older in-laws, and community leaders in encouraging women to plan pregnancies and seek healthcare advice ~~noted that older in-laws and community leaders play a key role in encouraging women to plan their pregnancies and seek healthcare advice beforehand.~~ However, cultural beliefs often dictate that once a woman is pregnant, she must continue the pregnancy, regardless of her mental readiness. ~~many women, along with their husbands and mothers-in-law, believe that once a woman is pregnant, she must proceed with the pregnancy, regardless of her mental readiness.~~ In some areas~~regions~~, women fear abandonment if they ~~may hesitate to~~ leave their husbands~~, fearing abandonment~~ during pregnancy whiel~~. Additionally,~~ cultural norms discourage ~~women from~~ open~~ly~~ discussion~~ng~~ about family planning with in-laws or friends, causing embarrassment. ~~their desire to have children with in-laws or friends, leading to embarrassment. Some~~ Additionally, elders also promote seeking blessings from holy water rather than consulting healthcare professionals.

"What is the barrier to pre-pregnancy testing that Yau explained is the backward culture. Even though she knows it, she wants this, she wants to have a baby because she thinks it will hurt, or she is ashamed, it's a shake to be told this is what she wants, but they know it. It is very embarrassing to say that you want to have children with your mother-in-law or father-in-law, even with your friends. For example, I can tell them that I want to have a baby here and my baby is grown, I may even have a desire inside, but they can go out and accuse me of being shameful" ***(35 years old mother, 10th grade, FGD)***

Some would believe in the service and some would not, especially the old-times people (elders). *The elders may downplay the service like: "we are here and still managed to have kids despite there being ~~was~~ no such service in our time," and ask "Consulting who else were our grandmothers conceiving that you are doing so now?" and the like* ***(35 years old mother, 10th grade, IDI)***

On the other hand, some participants highlighted that older mothers emphasize the importance of pre-pregnancy care, particularly for women facing infertility ~~pointed out that community leaders and elder mothers advocate for pre-pregnancy care, particularly for women facing infertility to deliver a healthy baby. Furthermore, some elderly mothers advise women to discreetly seek guidance from healthcare providers~~

"They are very supportive of it. They are especially encouraging if you give them recognition when it comes to mothers and children. It's okay because it's normal in the normal. ~~But~~ however, women with the problem of being unable to get pregnant are encouraged to consult healthcare providers by the mothers because they want you to give birth to them. They even tell you to go in hiding" ***(Public health professional, male, KII)***

~~**Poorly perceived PCC for low-risk women:**~~

~~Participants noted that the community may criticize and socially pressure mothers without known health problems to seek PCC services, potentially leading to them being ridiculed.~~

~~*"The community criticizes or said to her as what does it mean to seek care before being pregnant without sick and replay to her this is joking"* ***(32 years old mother, 5th grade, IDI)***~~

~~**Husband opposes PCC:** Mothers iterated that a significant husband may influence women to uptake PCC services. The reason might be they do not perceive seeking care before pregnancy~~

~~for women with no known health issues. Participants also noted that except for some husbands with good literacy levels, most of them understand that PCC is not a priority issue, so not support to consult women's healthcare provider services before pregnancy.~~

~~*The majority of them (husbands) are obstacles except a few* ***(35 years old mother, not attended school, IDI****)*~~

~~*"Some husband makes fun of them. For example, I met a mother who gave birth to 10 children and told me to take contraception but my husband refused. He is still a son of a bitch. Imagine being unable to protect a mother with 9 children whose one is dead. Surprisingly, she told him to use protection in front of me and he joked that she would still be engaged/married"* ***(HEW, female, IDI)***~~

High ~~W~~workload

~~**Extra burden on the healthcare provider:** Healthcare providers~~HCPs noted that providing PCC services increases their workload and places additional burdens on staff, potentially leading to demands for extra benefits and staffing. When women come for child vaccinations and family planning but also request PCC, it requires additional time and commitment from healthcare providers. Providers highlighted that due to time constraints, some women were not screened.

"Implementing this program will increase our workload and place additional burdens on the staff, potentially leading to demands for extra benefits. This could pose a challenge if there is no allocated budget" ***(Midwifery professional, female, IDI)***

On the other hand one healthcare provider noted that, if we work on PCC it will make the provider's job easier because it decreases the risk factors like malnutrition, abortion and other

complications during pregnancy and childbirth.

…."For sure, but if it is done here, I think it will make our job easier for us. So we have worked on preconception care, and we are fixing this malnutrition ~~thing~~. His silence, which we call abortion, is being fixed while it is there. Stunting and underweight are being fixed there. So the professionals finish their work there" ***(Public health professional, male, KII)***

Services cost

Low socioeconomic status, service costs, and transportation expenses pose major barriers to accessing preconception care (PCC) services. While maternal health services like antenatal care, labor, delivery, and postnatal care are free in Ethiopia, participants highlighted that the costs of PCC, such as lab tests and imaging, are challenging for low-income mothers. This financial burden can result in missed opportunities for PCC, increasing the risk of preventable maternal and child health complications. Additionally, many individuals' priorities other needs over PCC if they feel healthy, and there is reluctance among mothers to seek care, even for routine checkups when ill.

"Regarding the affordability of PCC costs, a mother with a low income might not be able to afford to pay, especially for some lab tests and imaging procedures" ***(32 years old mother, 5th grade, IDI)***

*"If it has a cost, those who cannot afford it would miss the service, which could contribute to the occurrence of maternal and child health complications that could have been prevented otherwise "****(29 years old mother, 10th grade, IDI)***

~~**Distance to health facility:**~~ ~~A mother~~Mothers reported that, long distance ~~because of distance~~ to health facility and ~~,~~ ~~lack of money for transport~~ lack of money for transport often leads them to miss the PCC services.~~mothers may induce to for decision to miss the PCC services.~~

"The health facility may be too far from your house, you may face a shortage of money, and you may be unable to walk there. In that case, how are you supposed to get to the health facility? If you do not have transportation costs, it is necessary to stay at home. Lack of money will prevent you from doing many things. Then, you become stressed and decide to be ignorant" ***(35 years old mother, not attended school, IDI****)*

Nearly all mothers and HCPs agreed that PCC services should be accessible to individuals, regardless of economic status, to enhance maternal and child health, reduce costs, and promote equity. Participants stressed the importance of providing these services free of charge, like other MCH programs, to support low-income mothers at higher risk of health complications.

"The service should be free of charge. If it has a cost, those who cannot afford it ~~would~~ will miss the service, which could contribute to the occurrence of maternal and child health complications that could have been prevented otherwise" ***(29 years old mother, 10th grade, IDI)***

Formatted: Justified, Space After: 8 pt, Widow/Orphan control, Adjust space between Latin and Asian text, Adjust space between Asian text and numbers

Formatted: Font: (Default) Times New Roman, 12 pt

"The cost of service for PCC should be free, similar to the service provided for ANC. If there is a fee, the community will refuse it. The government should provide subsidies and exempt services related to PCC" ***(HEW, female, IDI)***

A healthcare provider working at MCH said that providing free PCC services is also difficult for ~~the health facility balanced payment may be good for sustain~~that providing free PCC services is also difficult for the health facility balanced payment may be good for sustaining the services.

"If it is a free service, the health facility may also suffer, but it should be at a reasonable price.

Formatted: Line spacing: Double

For example, a sugar test results in 70 or 80 birr. This should be reduced” ***(Public health professional, male, IDI).***

Formatted: Font: (Default) Nyala, Not Italic

Scarcity of medicines and medical equipment~~resources~~

The majority of both HCPs and mothers highlighted that shortages of medical equipment and medicines such as iron and folic acid supplements, anti-hepatitis vaccines, immunoglobulin, laboratory reagents, essential drugs, and diagnostic tests like ultrasound pose significant challenges to PCC services, particularly at health centers. Similarly they mentioned that due to the scarcity of medical supplies and medications, women are frequently referred to hospitals, exacerbating the situation, especially amid the crisis in the study area (Tigray). A participant working in the delivery unit highlighted a concerning trend of congenital anomalies, which they suspect may be linked to the shortage of folic acid supplements. Additionally, participants reiterated the challenges posed by the lack of guidelines and the shortage of healthcare providers.

“Most of the preconception care services, such as iron and folic acid supplements, ultrasounds, and lab tests for HIV/STI, are not available at the health center~~e~~” ***(29 years old mother, 10 th grade, IDI)***

There is no guideline~~s~~ and a shortage of healthcare providers. …….”A mother who is Hepatitis positive should get ~~anti~~ Hepatitis vaccine and immunoglobin. But we don't have this drug in the facility” ***(General practitioner professional, male, IDI)***

~~On the other hand, some midwives working at MCH described that no need of extra resources for PCC, it can provide together with the routine activities and not demand extra human resources.~~

~~*"I don't believe it requires additional resources. While we are completing our daily tasks, we can*~~

offer the women advice. We will serve the person who has already arrived for another primary complaint if it is in OPD. We can even tell women when they come here for family planning, or EPI. We complete this easy task in addition to our daily responsibilities. Not all of them call for additional human resources. We can combine it with other activities" ***(Midwifery professional, female, IDI)***

Poor quality of family planning services: The study participants identified a lack of access to contraceptives and inadequate contraceptive counseling services as reasons for unintended pregnancies.

"The healthcare provider is also sending mothers for each contraceptive without properly explaining what is wrong with it and what kind of mother it should be given to" ***(General practitioner professional, male, IDI)***

Poor adherence to iron-folic acid supplementation: Due to the frequently cited side effects of gastritis, many mothers, including those with a history of APOs such as congenital anomalies, do not consistently take iron and folic acid, as some participants pointed out.

"In our experience, mothers frequently disregarded our advice to take folic acid. They regularly gave reasons for not complying, such as gastritis, as side effects. A mother in a recent congenital anomaly case acknowledged not taking folic acid or stopping the medication because she had gastritis" ***(Midwifery professional, female, IDI)***

A healthcare participant mentioned a prevalent rumor among primigravida women that iron should not be taken because it can cause the baby to grow too large, making delivery difficult and increasing the risk of surgery.

"If they are primigravida, there is a rumor that iron should not be taken because it makes the baby grow too big, is difficult to deliver by uterus, and exposes them to surgery, even if we tell

~~*them its benefits properly" **(Midwifery professional, female, IDI)***~~

4. Opportunities of ~~for improving of awareness of~~ PCC services ~~and uptake~~

~~**SMART Start model:** The SMART Start model focuses on financial preparation and achieving optimal health before pregnancy through contraception, ensuring healthy children. Participants noted that this model highlights the importance of preparation for pregnancy, akin to pre-pregnancy care. To raise awareness and increase the uptake of PCC, the SMART Start model should be integrated into community health services as an entry point for couples, particularly adolescent couples, through activities conducted by WDGs, HEWs, and other social networks.~~

~~*SMART Start is very good especially because it advocates for adolescent girls to use contraceptives and it creates awareness before pregnancy, so that we can use it. Similarly as you have mentioned very well women's development groups can be used as an entry path way as we know it is vital to be aware of adolescent girls regarding preconception care **Clinical midwifery professional, male, KII)***~~

~~Utilize the~~ ~~E~~existing community platforms: Participants emphasized the crucial role of the HEP in ongoing PCC awareness at the community level. They suggested enhancing this program by involving HEWs with WDGs and village health leaders. Participants also highlighted that home-to-home visits strengthen PCC, as some women may feel uncomfortable discussing their desire to conceive openly.

"To make the program~~me~~ effective, it is essential to support WDGs and HEWs. In my previous work, I have seen the significant impact of WDGs in addressing public health issues. They have detailed community knowledge ~~of the community~~ and can provide valuable information about vaccinated children, pregnant mothers, and other relevant data. By working with community

leaders and WDGs, we can increase the acceptance and success of the program" ***(Midwifery professional, female, IDI)***

A Health extension worker said:

"The home-to-home visit should be well strengthened because the woman might have felt ashamed to disclose her desire to get pregnant" **(~~Nursing professional~~HEW, female, IDI)**

Formatted: Not Highlight

An MCH expert said *"A family health card is one. The service could be incorporated into the family health card checklist for the woman to read herself or to be read for her by her kids to improve their awareness"* ***(Public health professional, male ,KII)***

Participants suggested that marriage certificate issuance venues could ~~serve as~~ beeffective platforms for PCC education and counseling. These venues offer couples an entry point for information and provide an opportunity to counsel them in advance, linking eligible women to health facilities.

"Places where marriage certificate is provided would be even much better and of high importance to provide PCC service because it gives an opportunity for the couples to get the information before the start of sexual intercourse" ***(35 years old mother, 10 th grade, IDI)***

Social networks such as civic societies, women's development groups, and farmer's associations are effective for community mobilization and spreading information. Participants highlighted their role in increasing PCC awareness and uptake through education and counseling at local venues including churches, mosques, and traditional healing places.

We can use the existing social networks such as civic societies (e.g. women's associations), women's development army, pregnant women forum…etc . Those social networks are the closet to the community they can provide education to the mothers. First, the social networks need to be well aware of PCC and then provide roles and responsibilities to teach and mobilize mothers to

utilize the services. The social networks are very helpful in strengthening and decentralizing preconception care services ***(29 years old mother, 10th grade, IDI)***

SMART Start model: The SMART Start model focuses on financial preparation and achieving optimal health before pregnancy through contraception, ensuring healthy children. Participants noted that this model highlights the importance of preparation for pregnancy, akin to pre-pregnancy care. To raise awareness of PCC and uptake, the services would be integrated in to the SMART Start model program as an entry point for couples, particularly adolescent couples, through activities conducted by WDGs, HEWs, and other social networks.

Formatted: Font: Not Bold

*SMART Start is very good especially because it advocates for adolescent girls to use contraceptives and it creates awareness before pregnancy, so that we can use it. Similarly as you have mentioned very well women's development groups can be used as an entry path way as we know it is vital to be aware of adolescent girls regarding preconception care (M****idwifery professional, male, KII)***

Formatted: Space After: 0 pt, Line spacing: Double, Don't adjust space between Latin and Asian text, Don't adjust space between Asian text and numbers

Formatted: Font: Not Bold

~~**Integrating PCC services into existing health facilities:** Many study participants suggested to integrate PCC with existing health services, such as voluntary counseling and testing, family planning units, cervical cancer screening units, youth-friendly services, and antiretroviral therapy (ART) clinics, to create awareness about PCC through education for women of reproductive age who visit the health facility for other reasons using RLP tool.~~

Formatted: Line spacing: Double

~~*"All people who visit a health facility should get information on PCC. We can use health facilities as a means to disseminate information and create awareness"* ***(HEW, Female, IDI)***~~

~~*"As a PCC, I believe that any female patient in the reproductive age group should be able to see you whenever they need something, like an abortion, a STI test, FP, a pregnancy test, or a PNC. Right now, all women should receive PCC counseling"* ***(General practitioner professional,***~~

male, IDI)

Perceived desire interest of healthcare providers of in PCC services: Though there would be a fear of workload, almost all healthcare providers appreciated the services' the benefit of the services in preventing all of the maternal and neonatal problems.

…"There is also a higher need for PCC in the professional that can be described as an opportunity" ***(Public health professional, male, IDI)***

Its necessity is unquestionable! ***(Public health professional, male, KII)***

In addition, the majority of participants emphasized that, although these services are a new concept within the health system, mothers—especially those at high risk—have provided positive feedback.

"If the service is available plenty of mothers have a wish to get the service you desired" ***(35 years old mother, not attended school, IDI)***

This is both appropriate and acceptable. Had education arrived to us in this form, we would not be afflicted by the illnesses and issues that we currently face. We'll say "Amen" to this, bring it down to our people, and persuade them to start working on it. That's a smart idea ***(39 years old mother, 3rd grade, FGD)***

Maternal interest in PCC services: The majority of the participants pointed out that, though the services are a new concept for the health system, mothers give positive feedback, especially those living with a history of APOs.

A mother with a history of APOs said: "If the service is available plenty of mothers have a wish to get the service you desired" ***(35 years old mother, not attended school, IDI)***

Thus, this is both appropriate and acceptable. Had education arrived to us in this form, we

Formatted: Line spacing: Double

~~*would not be afflicted by the illnesses and issues that we currently face. We'll say "Amen" to this, bring it down to our people, and persuade them to start working on it. That's a smart idea* ***(39 years old mother, 3rd grade, FGD)***~~

5. Percived ~~S~~suggestions for increasing awareness of PCC and uptake

Home-based ~~preparation~~initiative: Participants pointed out ~~those~~ that women intending to become pregnant need to prepare at home in terms of food and money to ensure adequate nutrition, transportation, medications, and other necessities before and during pregnancy. Additionally, some participants emphasized the importance of communicating with their husbands to achieve a common understanding about the intention to get pregnant, being mentally prepared, and avoiding bad habits such as alcohol (including local beverages like ("Siwa, Areki, and Myes"), smoking, and other addictive substances as pre-pregnancy care.

~~*The woman should assess if the living condition of her life is suitable for getting pregnant. Ensure economical and psychological readiness first* ***(35 years old mother, 10th grade, IDI)***~~

~~Prior to~~before her pregnancy, she should have a plan and be prepared economically; other work-related activities will help her overcome the problems that may arise following pregnancy. In her home, she should communicate and have a common understanding with her husband about her pregnancy. When she plans to be pregnant, she should also avoid bad habits such as alcohol, smoking and~~,~~ other addictive substances (hashish), and not be comfortable working during pregnancy in her home ***(Family health professional, female, KII)***

Use various~~variety~~ communication strategies: All participants agreed that creating awareness for the infant program (PCC) through education on various platforms and social networks should

be the first step to improving awareness of PCC and uptake. They emphasized the need for clear, culturally sensitive education on preparation, seeking advice, and understanding preconception risks to address community beliefs, myths, and misconceptions. Utilizing media such as posters, billboards, and leaflets at ~~both~~ community and facility levels is crucial for enhancing awareness. Once the community understands the importance of PCC, they will be more likely to seek it out independently.

……"Anyway, they have to work on awareness creation. Education on the causes and risk factors should be given. Education can be provided~~given~~ in the facilities, in groups, and in the community ***" (28 years old mother, 8th grade, IDI)***

Any cultural or religious barriers are better addressed through education. We should educate the community in an understandable way for them. If we do so, I think the elders, community, and religious leaders would say yes, it is essential~~important~~; there are only benefits, not harms; let's make use of it; and so on, provided that we educate them. I don't expect the other way around. ***(35 years old mother, 10th grade, IDI)***

A Mother from the FGD said,~~:~~ *"I prefer most of the time, PCC ~~should~~ be given on Sunday because both wife and husband could be available at home and on this day most people are off from work. If the HEW/WDA conduct home visits after 11:00 AM to provide education about PCC half an hour is enough"* ***(28 years old mother, diploma, FGD)***

The study participants pointed out that ~~E~~education should be specific, pragmatic and model-based ~~study participicants pointed out.~~

Yes of course: it is often better to see than ~~to~~ hear to easily understand~~ing~~ and understand everything by looking at a picture: I think it would be better if he did the vegetables or other

things diet in pictures ***(Midwifery professional, female, IDI)***

If you give pretend or picture-based lessons or drama-assisted lessons, women understand easily ***(Public health professional, male, IDI)***

"Our education should be unique, which means that if we are pregnant, for example, from pre-pregnancy to pregnancy, our education should not be the same as the previous by saying "listen to her", I mean it should be practical" ***(Public health professional, male, KII)***

Participants suggested several strategies to increase awareness and uptake of PCC, emphasizing the use of various media. Broadcast media like radio and television are particularly effective due to their widespread availability. ~~Additionally, P~~print media, including posters, billboards, and leaflets, should be used at both community and facility levels. While HEWs play a crucial role, media influence is even more critical, as HEWs cannot reach every area. One participant noted that media can significantly raise awareness, stating that if people hear about PCC on the radio or television, they are more likely to seek health services and receive education through printed materials.

"The greatest power is the media. Whether it is through television, radio and magazines, the media is very crucial if you want to change the perception of the community about the PCC" ***(~~General practitioner professional~~MD, male, IDI)***

"Media has a big role. Our community follows the media. Although the role of HEWs is the biggest, the influence of media is more critical than anything because they can't reach every corner" ***(Nursing professional, female, IDI)***

Although orientation on PCC has recently commenced, it remains a new program. Consequently, nearly all individuals involved in the healthcare system, from HEWs to district health experts,

possess limited knowledge about PCC. Therefore, both short-term and long-term training are essential ~~for the effectiv~~effectivelye ~~provision~~ providing ~~of~~ these services. All participants emphasized that training and guidelines are crucial for the successful delivery of PCC services.

"Training on PCC packages is required for all actors involved in the health care system, starting from the HEW to district/woreda health experts (health care professionals)" ***(Family health professional, female, KII)***

~~**Community based PCC services:** The services should be accessible to the community with a reasonable distance, because distances to health facilities and transportation access were hinder mothers from seeking the services. A sizable portion of the study participants pointed out that, to enhance the unreasonable poor level of awareness of the community providing education to the community using the existing community platforms like women development army, women's affairs network, community gatherings and pregnant women forum through house to house.~~

~~*"We can provide education in their respective communities using the already existing platforms, such as the women's development army, the women's affairs network, community gatherings, and the pregnant women forum. We can reach the community through their already established networks and house-to-house"* ***(29 years old mothers, 10th grade, IDI)***~~

Community engagement: Some participants emphasized that creating significant awareness about preconception care at the community level requires special efforts, mainly~~particularly~~ focusing on extensive information dissemination and community engagement.

Special efforts are required, including robust community mobilization and active participation ***(HEW, female, IDI)***

Community health facility ~~L~~linkage: in general, participants noted that establishing a linkage between the community and the health facility, as well as within the health facility to selected

units providing PCC, using referral slips, would be vital for enhancing the uptake of PCC services and reducing delays in the health facility.

An MCH expert said*: a r~~R~~eferral slip is necessary~~important~~. Once the eligible woman~~women~~ is identified, the referral slip can be used to link to the service. But one thing that needs to be put in to consideration is if it is going to be free service or not. Maybe it could have some effect if it is payable service* ***(Public health professional, male, KII)***

Use high-risk mothers as role models for awareness creation:~~education~~: Some participants suggested that mothers with a history of APOs could serve as role models to educate women in women's development groups, pregnancy forums, and similar platforms. This approach could motivate the community to improve awareness and uptake of PCC.

"I consumed three or four beers until I gave birth while I was pregnant. Here, I found myself teaching others and myself about mental retardation, a health issue that my child had developed. Even if you have to consider it before getting pregnant, you should not drink while pregnant. However, since my son experienced it, I have been telling people about it once more.~~I am telling people about it once more~~. Since a baby is like a piece of paper and can be easily injured, I think that my son's alcohol abuse contributed to his mental retardation" ***(27 years old mother, 10th grade, IDI)***

"She also influenced other women who had difficulty conceiving. i.e., other women also seek health facilities after taking experience from my sister" ***(30 years old mother, diploma, FGD)***

~~**Need focus from government**~~

~~A sizable portion of study participants observed that while there was limited orientation or~~

information provided about PCC, the government should prioritize PCC on par with ANC and delivery services. There is an urgent need to enhance future PCC practices, encompassing ANC, family planning, and delivery services, to elevate community awareness. It is essential to foster meaningful engagement and collaboration among the government, stakeholders, political leaders, influential individuals, elders, and religious figures to improve awareness of and uptake in PCC.

"A kind of orientation or information was given about PCC by partners, but they still do not get that much focus from the government or partners on PPC like the other health care services. Through education, the community shows behavioural change in relation to child and maternal nutrition" ***(Family health professional, female, KII)***

Deploy healthcare providers: Participants emphasized that deploying healthcare providers, particularly HEWs, is essential for raising awareness of PCC through community platforms.

Preconception care services would be free of charge: It ensures that all individuals, regardless of their economic status, can access essential care that improves maternal and child health, reduces healthcare costs, and promotes equity and public health. Participants emphasized that PCC services should be free of charge, similar to other MCH services, to effectively reach mothers who face high rates of maternal and child health complications and have low socioeconomic status, as they might not be able to afford these services.

"The service should be free of charge. If it has a cost, those who cannot afford it would miss the service, which could contribute to the occurrence of maternal and child health complications that could have been prevented otherwise" ***(29 years old mother, 10th grade, IDI)***

"The cost of service for PCC should be free, similar to the service provided for ANC. If there is a fee, the community will refuse it. The government should provide subsidies and exempt services

related to PCC" ***(HEW, female, IDI)***

A healthcare provider working at MCH said since provide free of PCC services is also difficult to the health facility balanced payment may be good for sustain the services.

"If it is a free service, the health facility may also suffer, but it should be at a reasonable price. For example, a sugar test results in 70 or 80 birr. This should be reduced" ***(Public health professional, male, IDI)***

Services integration

Participants' view on PCC services provision:

Responsible professionals: Participants noted that since the service encompasses various interventions, multidisciplinary professionals such as nutritionists, gynecologists, midwives, and nurses would be responsible for the program. However, because HEWs frequently contact the community during home visits, midwives and gynecologists who encounter many women with a history of APOs should be given priority.

"For example, a gynecologist is needed, and you will need a nutritionist, and you will also involve him if you need one from him. For example, it is multidisciplinary because it involves others in the community, and I think PCC should be delivered by multiple professionals. For example, nutritionists, gynecologists, midwives, and nurses" ***(General practitioner professional, male, IDI)***

HCPs unanimously recommended integrating PCC into existing services, including VCT, FP units, cervical cancer screening, youth-friendly services, ANC, ART clinics, post-abortion care, EPI, and under-five clinics. This approach aims to provide services to educate women of reproductive age who visit health facilities for other reasons, using the RLP tool to identify those

eligible for PCC services effectively.

"All people who visit a health facility should get information on PCC. We can use health facilities as a means to disseminate information and create awareness" ***(HEW, Female, IDI)***

"As a PCC, I believe that any female patient in the reproductive age group should be able to see you whenever they need something, like an abortion, an STI test, an FP, a pregnancy test, or a PNC. Right now, all women should receive PCC counseling" ***(MD, male, IDI)***

~~**Integration of PCC into existing health services:** A sizable portion of the study participants suggested that PCC should be integrated or linked with existing health services. These services include ANC, VCT, ART clinics, FP units, post-abortion care units, cervical cancer screening units, youth-friendly services, EPI, and under-five units. The integration should focus on women of reproductive age who visit the health facility for other reasons, using the reproductive life plan tool as a triage method to identify eligible women for PCC services.~~

~~*"VCT, ART clinic, family planning unit, post abortion care unit and cervical cancer screening unit are the favorable places/units where reproductive age group women are found"* ***(Obstetrics and gynecology professional, male, KII)***~~

Formatted: Line spacing: Double

~~**Standalone PCC services:**~~ Some participants suggested that PCC services should be provided as standalone services in room, as this would be more user-friendly. Integrating PCC with other services might make women uncomfortable. However, due to limited space, staff, and resources, offering PCC under the ANC unit could be beneficial, as it allows for the observation and advice on pregnancy-related abnormalities. Some participant suggests that PCC services could be

offered alongside family planning services, where women typically seek to prevent pregnancy, providing an opportunity to discuss PCC.

Discussion

This study examined the experiences, challenges, and opportunities of PCC services in Tigray, Ethiopia, ~~Our uniquely explores the perspectives on PCC services in Tigray, Ethiopia,~~ following the government's prioritization~~endorsement~~ of PCC as a key strategy and the development of new guidelines (23) (34) (35).~~(23) (31) (32)~~. While previous qualitative research on PCC focused solely on barriers to its uptake (36), this study took a broader approach by exploring the awareness, experiences, challenges, and opportunities related to PCC services from the perspectives of various participants.

The study identified key themes related to PCC services, including awareness (information and perceived benefits) and experience (practices of PCC interventions and home-based preparation). Participants also explored perceived challenges, such as traditional beliefs and misconceptions, fragmented service delivery, reluctance to disclose conception intentions, inadequate counseling on contraceptive services, societal norms, heavy workloads, high costs, and shortages of medicines and medical equipment. Additionally, they noted perceived opportunities, such as a strong interest in PCC services and the availability of community platforms. Suggestions for improvement included implementing home-based initiatives, adopting diverse communication strategies, involving high-risk mothers as role models, fostering community engagement, strengthening links between communities and healthcare facilities, and integrating PCC services within the healthcare system.

~~We explored women's experiences and healthcare providers' knowledge of PCC services, uncovering key challenges such as low awareness, misconceptions, workloads, costs, poor quality services, and fear of contraceptive side effects. To improve awareness and increase~~

uptake, participants suggested promoting home-based preparation, integrating the SMART Start model, leveraging community platforms, offering free services, using high-risk mothers as role models, and focusing on diverse communication strategies.

In the study, except a few high-risk mothers, most mothers lack awareness of PCC services, which is consistent with previous studies (21, 36-38). Most women were unaware of PCC, but some high-risk women recognized its benefits and prepared at home. These women received fragmented care, and HCPs believed PCC should prioritize them by ensuring access to information, a finding consistent with other studies (21, 33-35). Many people may be unfamiliar with PCC services because the program was introduced only recently (23) (34) (35) and has not yet been fully integrated into the healthcare system. This is especially evident within the health extension program, which is vital for increasing community awareness. This study highlights the issue, showing that HEWs possess less information about the program compared to other HCPs. The study found that although most HCPs recognize the benefits of PCC in reducing APOs, they primarily offer limited PCC interventions to high-risk mothers. Although included in health policy, HCPs treat PCC as an extra benefit rather than a routine practice, leading to minimal investment and delayed care-seeking until pregnancy is confirmed. Additional challenges include poor awareness among women, a lack of dedicated HCPs, inaccessible guidelines, limited government focus, inadequate training, and insufficient knowledge among frontline healthcare providers. Participants suggested promoting home-based preparation, using varied communication methods, and highlighting high-risk mothers as role models. Research, including a study from Ethiopia (33), emphasizes the need for new communication strategies to raise awareness of PCC. These findings are consistent with a study conducted in South Africa (36). Additionally, PCC in six European nations (37) focuses on high-risk women, while others receive it opportunistically. In the United states, PCC services are

recommended as part of routine healthcare for all women, regardless of pregnancy intention or risk (38). This study found that healthcare providers did not practice most of the PCC interventions recommended by the WHO and Ethiopia's national PCC guidelines (2) (32) consistent with findings from other studies in LMICs like Ethiopia (22).

Furthermore, HCPs often view PCC as an optional benefit rather than a standard service, leading to limited investment and delayed care-seeking until pregnancy is confirmed. Such neglect can hinder health-seeking behavior and the utilization of services. Overall, participants emphasized that enhancing PCC awareness should be a government priority, leveraging community engagement through social networks like civic organizations, women's groups, and farmers' associations, which are effective for mobilization and information sharing. Home-based preparation was identified as a promising foundation, alongside diverse communication methods and integrating PCC services into healthcare facilities to improve awareness and utilization. Evidence also showed that to enhance PCC awareness, massive public awareness campaigns and education through print media, social forums, and government-led initiatives including integrating PCC into the healthcare services (19, 39).

This study revealed that while most HCPs had some knowledge of PCC services, their practice was limited. The services mainly focused on specific components targeting high-risk mothers such as contraceptive counseling for pregnancy delay, medication safety, substance use counseling, and folic acid supplementation. However, most interventions recommended by the WHO and Ethiopia's national PCC guideline were not practiced (2) (35). This is consistent with previous findings from African countries, where PCC was implemented under inaccessible guidelines and primarily driven by initiatives targeting high-risk women with a limited focus on interventions such as dietary modification counseling (22) (40) (41). The limited practice of PCC

is influenced by several factors such as the presence of a relatively new program, HCPs' lack of adequate training, and PCC guidelines not being easily accessible. Moreover, the government prioritizes ANC over PCC, leading healthcare providers to view PCC as a supplementary rather than a routine service. Evidence suggests that accessible guidelines and regular use of RLP tools can help HCPs prioritize PCC and emphasize preconception care for women (19) (22). Hence, prioritizing PCC and integrating package-based services into routine care for all women of reproductive age using RLP tools could significantly enhance awareness and utilization.

Formatted: Line spacing: Double

~~The study found that misconceptions about conception, family planning, and alcohol intake pose challenges to PCC awareness and uptake, negatively impacting maternal and child health. Additionally, HEWs' focus on ANC and institutional delivery may cause these issues to be overlooked. Integrating PCC with family planning services and raising awareness about these misconceptions is crucial, as family planning clinics can enhance preconception health by screening for pregnancy intentions using a reproductive life plan tool (39). This integration helps women prepare for pregnancy and enables healthcare providers to routinely screen for pregnancy intentions using a reproductive life plan tool. Asking, "Would you like to become pregnant in the next time?" is more acceptable than other methods for measuring pregnancy intention (40). This question helps healthcare providers decide whether to offer PCC or other reproductive health services, at community and facility levels, a practice recommended and supported by other studies. Participants in this study also recommended this approach, which is supported by other studies (36). A study in China supported the integration of planned pregnancy and preconception care services (41).~~

Formatted: Indent: Left: 0.5", Line spacing: Double

Evidence indicates that, without adequate counseling, clients often turn to friends and family for information. Unfortunately, this information is frequently influenced by misinformation (44). For example, in this study, misconceptions about contraceptives, such as the Depo-Provera injection, have led many women to be discouraged or even prevented from using them. These misconceptions, including unfounded fears that contraceptives may cause infertility or congenital anomalies, present significant challenges to raising awareness about PCC and improving its uptake. HEWs also give due emphasis to the maternal continuum of care; however, they do nothing for PCC as a package that could be considered a missed opportunity. Evidence shows that family planning unit is vital in improving preconception health by assessing pregnancy intentions and addressing misconceptions through tools like the RLP (45). Therefore, integrating PCC into family planning and antenatal services strengthens the HEP with culturally sensitive education, and utilizing health visits for PCC education can significantly enhance services and reduce misconceptions about contraceptive use.

The study revealed that traditional beliefs often compel women to keep their conception desires private avoiding discussions about PCC. Pregnancy is commonly regarded as a divine gift from St. Mary or God, and many women believe that expressing a desire to conceive before pregnancy contradicts divine will, making such discussions socially and personally taboo. The present study highlighted that undisclosed conception desires pose a major challenge for PCC service utilization. Even when women plan their pregnancies, they often hesitate to seek or discuss PCC due to cultural influences and a reluctance to share their plans with others, including health professionals. The study found that, with few exceptions, most women prefer to keep their desire to have a child confidential. They often conceal their pregnancies during the early months until they rule out the risk of miscarriage, and they typically discuss their plans only with their husbands and a very close friend. Women avoid discussing pre-pregnancy care because it is often considered taboo or

Formatted: Space Before: 0 pt, After: 0 pt, Line spacing: Double, No widow/orphan control, Don't adjust space between Latin and Asian text, Don't adjust space between Asian text and numbers

Formatted: Font:

Formatted: Line spacing: Double

embarrassing. Additionally, many view pregnancy as a divine gift from St. Mary or God, and they believe disclosing the desire to conceive before pregnancy contradicts divine will. This ~~finding aligns with the results of a previous study conducted~~ is consistent with other studies elsewhere (36, 42), indicating that women often perceive conception as a natural event requiring no preparation, resulting in limited communication with healthcare providers and missed opportunities to utilize PCC services. Evidence suggests that addressing traditional beliefs about conception requires collaboration between health institutions and religious organizations (36). Additionally, integrating preconception health discussions into routine healthcare, beginning with school-based services, has been proposed as a potential solution (43). ~~(33) (35) (42). In this study participants noted improve awareness and to decrease the cultural belief of disclosure of conception using the existing community platforms mainly HEP of HEWs and WDGs home to home visits strengthen PCC, as some women may feel uncomfortable discussing their desire to conceive openly~~. In this study, ~~P~~participants suggested that women prefer receiving PCC at health posts and health centers, as well as through ~~in addition to~~ community-based services, because these settings align with cultural norms and are closer to home, making it easier for women to disclose personal matters.

Involving husbands in PCC through screenings and counseling is vital for reducing risks and promoting healthier pregnancies, as emphasized by Ethiopian (35) and Chinese national guidelines (46). Furthermore, women who made decisions independently or in partnership with their husbands were more likely to utilize PCC services (47) (48) our findings showed that~~In the study~~, husbands often ~~frequently~~ oppose~~d~~ women's PCC uptake in rural area ~~, especially in rural and less educated settings~~. ~~Other studies found that women who made decisions independently or jointly with their husbands were more likely to use PCC than those who could not decide for themselves (43). The study highlights the lack of husband involvement in pregnancy planning,~~

especially in rural areas, and suggests couple-based services to address this issue. Involving husbands in PCC through screenings and counseling is crucial for reducing risks and promoting healthier pregnancies, as supported by Ethiopian (32) and Chinese national guidelines(41). Poor parental behaviors before conception are linked to increased illness and mortality in offspring, while healthier habits can significantly improve pregnancy outcomes. Paternal preconception care emphasizes men's direct contributions to child health, such as genetic and epigenetic factors, lifestyle choices, and environmental exposures, as well as indirect influences through partner health and relationships (49). The paternal origins of health and disease model defines (50) the preconception population as all reproductive-age men and women, highlighting the importance of paternal health. Given men's crucial role in reproductive health decisions, couple-based counseling is strongly recommended (44).

The study found that poor socioeconomic status, including service costs and distance, hinders PCC use, with some viewing pregnancy as a luxury due to basic needs and regional conflicts. These findings align with results from various other studies (33) (44). The implication is that even when women acknowledge the need for PCC and wish to access it, they face additional challenges such as service charges and transportation costs. Participants suggested that women prefer receiving PCC at health posts and centers, alongside community-based services. They feel more comfortable discussing personal matters in culturally familiar, local settings rather than hospitals. This preference helps address transportation costs, offers clearer education, and overcomes barriers related to disclosing pregnancy intentions, which is a major obstacle to PCC utilization. Besides, the current study emphasizes that healthcare providers and mothers strongly advocate for making PCC services free, as they are part of the maternal continuum of care. This could improve access, particularly for low-income mothers, enhance maternal and child health, reduce costs, and promote equity. Other study in

China support this, with China’s National Free Preconception Health initiative, for example, reaching over 95% of target couples with preconception health education (46). Additionally, free maternal healthcare including PCC aligns with the WHO's recommendation to eliminate financial barriers and ensure equitable access to healthcare for all. It supports achieving SDG 3.1, which aims to reduce maternal mortality to fewer than 70 per 100,000 live births by 2030 (53).

Formatted: Line spacing: Double

To enhance awareness of PCC and uptake ~~of PCC~~, the study suggests using marriage certificate settings to provide couples with information and pre-counseling, thereby connecting eligible women to health facilities. This approach could be practical~~effective~~ if the Ethiopian government, in collaboration with religious leaders, commits to issuing marriage certificates, particularly for newly married couples. This recommendation aligns with findings from a study in Bangladesh (45). Marriage certificate settings present an ideal opportunity to introduce PCC services~~,~~ as couples are often in the early stages of planning their future. This universal process provides an equitable platform to educate couples on reproductive health, family planning, genetic risks, and the importance of preparing for a healthy pregnancy.

In Ethiopia, the PCC program was integrated into the reproductive health strategy for 2020–2024/25(23), and the components of PCC interventions were incorporated into the 2022 ANC guidelines (34). Additionally, the 2024 PCC guidelines recommend providing integrated services to women planning to conceive within three months (35). However, in this study, PCC services were only partially implemented hindered by the absence of PCC guidelines, limited government focus, inadequate training, and fragmented service delivery which heavily relied on women's

initiation. PCC services were neither prioritized nor adequately resourced, resulting in inconsistent practices and insufficient attention to this critical care area. High-risk women were often not offered PCC unless they explicitly requested it, and PCC was not routinely utilized to assess reproductive intentions even for women who had stopped using contraception to conceive. These findings align with studies from other parts of Africa (41, 55). In contrast, studies (46, 56) indicated that fully implemented services supported by established protocols and strong government backing, improve service utilization compared to fragmented approaches. Overall, the Ethiopian government should prioritize PCC and ensure its full implementation by integrating it into the existing healthcare system. This requires the inclusion of comprehensive PCC guidelines, protocols, and assessment tools as part of routine clinical practice.

According to the Motivational Theory of Role Modeling, role model-based interventions are highly effective in encouraging others to accept new services (57). In our study, participants suggested that high-risk mothers could act as role models to enhance awareness and encourage using PCC services.

Formatted: Line spacing: Double

In the study, all participants agreed that PCC services should be free, ensuring access for all, particularly low-income mothers, to improve maternal and child health, reduce costs, and promote equity. This is supported in other study (46). In the study, many participants suggested integrating PCC with existing health services like ANC, VCT, ART clinics, FP units, and more, targeting women of reproductive age using the reproductive life planRLP tool. This is also in concurrent with other studies in Africa Concurrent with other studies in Africa (4, 5). (4, 5). Most preconception counseling was provided opportunistically by HCPs working in gynecology clinics or family planning settings (58). Integrating services can improve access for women with

limited awareness of available options, those not actively planning or considering pregnancy, those with low health-seeking behaviors, and those without identified health issues. By routinely offering these services to all women of reproductive age through an integrated approach that includes the RLP tool, the uptake of PCC services can be improved (19, 22).

Formatted: Line spacing: Double

Study limitations and s~~S~~trengths and~~ limitations~~: This first study in Ethiopia explores PCC services from multiple perspectives, including front-line HCPs in urban and rural areas. The findings significantly contribute to developing locally appropriate programs to increase awareness of PCC and uptake~~The findings significantly contribute to the development of locally appropriate programs aimed at increasing PCC utilization~~. However, due to resource constraints, our study did not explore ~~, the study has limitations. Resource constraints prevented us from including the views of~~ husbands, religious leaders, and women without pregnancy histories, highlighting the need for further research. To maintain trustworthiness, we held open discussions and regular meetings among the researchers. However, because the name of the services is unfamiliar to the community, some women may have confused PCC with ANC, potentially leading to exaggerated responses.

Conclusions ~~and recommendations~~~~:~~ This study reveals ~~Most~~that most women lack awareness ~~of~~ about PCC~~, though some high risk women are informed and make home-based preparations before pregnancy.~~. HCPs deliver fragmented PCC interventions without using standardized guidelines or assessment tools focusing mainly on high-risk cases. Key challenges and opportunities identified include fragmented PCC services, traditional beliefs and misconceptions, inadequate counseling on contraceptive services, social influences, perceived demand for PCC, and the

potential to leverage existing community platforms. Integrating services into the existing community platforms, particularly within the HEP, is recommended to enhance awareness of PCC. Diverse communication strategies, including media campaigns and educational initiatives, should address traditional beliefs, misconceptions, and low community awareness.~~Both women and healthcare professionals agreed that contraceptive counseling, folic acid supplementation, and substance use counseling are commonly practiced components of PCC interventions. However, providers often apply these services in a fragmented manner, primarily targeting high-risk women. The study highlighted some of the challenges to PCC services, including low awareness, misconceptions, fear of contraceptive side effects, and poor-quality family planning.~~

~~To enhance PCC awareness and uptake, package-based services are essential. Participants recommended using diverse communication strategies, such as training, media platforms, and educational initiatives, to address misconceptions and gaps in knowledge. Participants also suggested strengthening home-based preparation, using high-risk mothers as role models, and integrating the SMART Start model into services. Additionally, they emphasized incorporating PCC into the HEP and routine services, identifying eligible women with the RLP tool, including them in family folders, and linking them to health facilities with referral slips.~~Additionally, participants suggested to strengthening the existing home-based initiatives and leveraging high-risk mothers as role models to promote awareness of PCC. Moreover, a package-based approach need to be implemented by integrating PCC as a continuum of maternal care. Introducing the concept of RLP is essential, enabling routine use of RLP tools to identify eligible women for these services. These findings provide valuable insights for Ethiopia's Ministry of Health, guiding policymakers, and program designers to enhance PCC utilization. Additionally,

innovative interventions should focus on establishing package-based services, creating demand, and strengthening service linkages for better outcomes.

Abbreviations

ANC: Antenatal Care

APOs: Adverse Pregnancy Outcomes

ART: Anti retro-viral therapy

CDC: Centers for Disease Control and Prevention

EPI: Expanded Program Immunization

FP: Family planning

HCPs: Healthcare providers

HEP: Health Extension Program

HEWs: Health Extension Workers

MNCH: Maternal, Neonatal and child health

OPD: Out Patient Department

PCC: Preconception Care

RLP: Reproductive Life Plan

SDGs: Sustainable Development Goals

VCT: Voluntary counseling Test

WDGs: Women Development Group

WHO: World Health Organization

Formatted: Normal, Left, Line spacing: Double

Formatted: Font color: Auto

References

Formatted: Line spacing: Double

1. Dean SV, Lassi ZS, Imam AM, Bhutta ZA. Preconception care: closing the gap in the continuum of care to accelerate improvements in maternal, newborn and child health. Reproductive Health. 2014;11:1-8.

2. World Health Organization. Meeting to develop a global consensus on preconception care to reduce maternal and childhood mortality and morbidity: World Health Organization Headquarters, Geneva, 6–7 February 2012: meeting report. 2013.

3. World Health Organization.. Preconception care. WHO Regional Office for South-East Asia; 2014.

4. Health NDo. Guidelines for Maternity Care in South Africa: A Manual for Clinics, Community Health Care Centres and District Hospitals. Pretoria. . 2015;Report No.: 4th edition.

5. Health MoP, Sanitation, Services MoM. National Reproductive Health Strategy 2009–2015. 2009.

6. Ethiopia Moh. National Antenatal Care Guideline, Ensuring Positive Pregnancy Experience. 2022.

7. Ethiopia Moh. National Preconception care Guideline, Ensuring a risk free conception experience 2024.

8. World Health Organization. Trends in maternal mortality 2000 to 2017: estimates by WHO, UNICEF, UNFPA, World Bank Group and the United Nations Population Division: executive summary. World Health Organization; 2019.

9. Quenby S, Gallos ID, Dhillon-Smith RK, Podesek M, Stephenson MD, Fisher J, et al. Miscarriage matters: the epidemiological, physical, psychological, and economic costs of early pregnancy loss. The Lancet. 2021;397(10285):1658-67.

10. Chawanpaiboon S, Vogel JP, Moller A-B, Lumbiganon P, Petzold M, Hogan D, et al. Global, regional, and national estimates of levels of preterm birth in 2014: a systematic review and modelling analysis. The Lancet Global Health. 2019;7(1):e37-e46.

11. Organization WH. Congenital anomalies. Fact sheet N 370. Media centre World Health Organization, Geneva; 2014 http://www who int/mediacentre/factsheets/fs370/en/ Accessed 10 Jan 2015. 2012.

12. Moges N, Anley DT, Zemene MA, Adella GA, Solomon Y, Bantie B, et al. Congenital anomalies and risk factors in Africa: a systematic review and meta-analysis. BMJ Paediatrics Open. 2023;7(1).

13. Tamirat KS, Sisay MM, Tesema GA, Tessema ZT. Determinants of adverse birth outcome in Sub-Saharan Africa: analysis of recent demographic and health surveys. BMC Public Health. 2021;21(1):1-10.

14. Csa I. Central statistical agency (CSA)[Ethiopia] and ICF. Ethiopia demographic and health survey, Addis Ababa, Ethiopia and Calverton, Maryland, USA. 2016;1.

15. EPHI I. Ethiopian Public Health Institute (EPHI)[Ethiopia] and ICF. Ethiopia Mini Demographic and Health Survey 2019: Key Indicators. 2019.

16. Berihu BA, Welderufael AL, Berhe Y, Magana T, Mulugeta A, Asfaw S, et al. High burden of neural tube defects in Tigray, Northern Ethiopia: Hospital-based study. PLoS One. 2018;13(11):e0206212.

17. Gesesew HA, Berhe KT, Gebretsadik S, Abreha M, Haftu M. Fistula in War-Torn Tigray: A Call to Action. International journal of environmental research and public health. 2022;19(23):15954.

18. Legesse AY, Teka Tseghay H, Abraha HE, Fisseha G, Ebrahim MM, Tsadik M, et al. Maternal mortality during war time in Tigray, Ethiopia: A community-based study. BJOG: An International Journal of Obstetrics & Gynaecology. 2024;131(6):786-94.

19. Ukoha WC, Mtshali NG, Adepeju L. Current state of preconception care in sub-Saharan Africa: A systematic scoping review. African Journal of Primary Health Care & Family Medicine. 2022;14(1):3096.

20. Woldeyohannes D, Tekalegn Y, Sahiledengle B, Hailemariam Z, Erkalo D, Zegeye A, et al. Preconception care in sub-Saharan Africa: A systematic review and meta-analysis on the prevalence and

its correlation with knowledge level among women in the reproductive age group. SAGE Open Medicine. 2023;11:20503121231153511.

21. Ayele AD, Belay HG, Kassa BG, Worke MD. Knowledge and utilisation of preconception care and associated factors among women in Ethiopia: systematic review and meta-analysis. Reproductive health. 2021;18:1-15.

22. Kassa A, Human S, Gemeda H. Level of healthcare providers' preconception care (PCC) practice and factors associated with non-implementation of PCC in Hawassa, Ethiopia. Ethiopian journal of health sciences. 2019;29(1).

23. Health EMo. Health Sector Transformation Plan II (HSTP II): 2020/21-2024/25 (2013 EFY-2017 EFY). MOH; 2021.

24. Atrash H, Jack B. Preconception care to improve pregnancy outcomes: the science. Journal of Human Growth and Development. 2020;30(3):355-62.

25. Mason E, Chandra-Mouli V, Baltag V, Christiansen C, Lassi ZS, Bhutta ZA. Preconception care: advancing from 'important to do and can be done'to 'is being done and is making a difference'. Reproductive health. 2014;11:1-9.

26. Abraha H, Ebrahim M, Teka H, Yemane A, Mulugeta A. Armed conflict drives most births to occur out of health facilities: evidence from Tigray, Ethiopia. drugs. 2023;2:3.

27. Palinkas LA, Horwitz SM, Green CA, Wisdom JP, Duan N, Hoagwood K. Purposeful sampling for qualitative data collection and analysis in mixed method implementation research. Administration and policy in mental health and mental health services research. 2015;42:533-44.

28. Wong LP. Focus group discussion: a tool for health and medical research. Singapore Med J. 2008;49(3):256-60.

29. Guest G, MacQueen KM, Namey EE. Applied thematic analysis: sage; 2012.

30. Vaismoradi M, Turunen H, Bondas T. Content analysis and thematic analysis: Implications for conducting a qualitative descriptive study. Nursing & health sciences. 2013;15(3):398-405.

31. health_Ethiopia Mo. Ensuring Positive Pregnancy Experience. National Antenatal Care Guideline. 2022.

32. health_Ethopia Mo. Ensuring a risk free conception experience! National Preconception care Guideline. 2024.

33. Teshome F, Kebede Y, Abamecha F, Birhanu Z. Why do women not prepare for pregnancy? Exploring women’s and health care providers’ views on barriers to uptake of preconception care in Mana District, Southwest Ethiopia: a qualitative study. BMC Pregnancy and Childbirth. 2020;20:1-13.

34. Chutke AP, Doke PP, Gothankar JS, Pore PD, Palkar SH, Patil AV, et al. Perceptions of and challenges faced by primary healthcare workers about preconception services in rural India: A qualitative study using focus group discussion. Frontiers in Public Health. 2022;10:888708.

35. Siraha E, Mukona D, Zvinavashe M, Dodzo L. Perceptions of preconception care among pregnant women at Masvingo general hospital, Zimbabwe: a qualitative study. Journal of Midwifery and Reproductive Health. 2020;8(2):2220-9.

36. Ukoha WC, Mtshali NG, editors. Perceptions and practice of preconception care by healthcare workers and high-risk women in South Africa: A qualitative study. Healthcare; 2021: MDPI.

37. Shawe J, Delbaere I, Ekstrand M, Hegaard HK, Larsson M, Mastroiacovo P, et al. Preconception care policy, guidelines, recommendations and services across six European countries: Belgium (Flanders), Denmark, Italy, the Netherlands, Sweden and the United Kingdom. The European Journal of Contraception & Reproductive Health Care. 2015;20(2):77-87.

38. Moos M-K, Dunlop AL, Jack BW, Nelson L, Coonrod DV, Long R, et al. Healthier women, healthier reproductive outcomes: recommendations for the routine care of all women of reproductive age. American journal of obstetrics and gynecology. 2008;199(6):S280-S9.

39. Klerman LV. Family planning services: an essential component of preconception care. Maternal and child health journal. 2006;10:157-60.

40. Baldwin MK, Overcarsh P, Patel A, Zimmerman L, Edelman A. Pregnancy intention screening tools: a randomized trial to assess perceived helpfulness with communication about reproductive goals. Contraception and reproductive medicine. 2018;3:1-5.

41. Xu J, Li X, Zhou Q. Nationwide-free preconception care strategy: experience from China. Frontiers in Public Health. 2022;10:934983.

42. van der Zee B, de Beaufort ID, Steegers EA, Denktaş S. Perceptions of preconception counselling among women planning a pregnancy: a qualitative study. Family practice. 2013;30(3):341-6.

43. Asresu TT, Hailu D, Girmay B, Abrha MW, Weldearegay HG. Mothers' utilization and associated factors in preconception care in northern Ethiopia: a community based cross sectional study. BMC pregnancy and childbirth. 2019;19:1-7.

44. Lang AY, Bartlett R, Robinson T, Boyle JA. Perspectives on preconception health among migrant women in Australia: a qualitative study. Women and Birth. 2020;33(4):334-42.

45. Jahan S, Shermin S. Retracted: Preconception Care through Religious Leaders in Bangladesh–A Novel Approach. Delta Medical College Journal. 2018;6(2):59-61.

46. Zhang S, Wang Q, Shen H. Design of the national free proception health examination project in China. Zhonghua yi xue za zhi. 2015;95(3):162-5.

Date: January 11/2024

Manuscript Number: PONE-D-24-41198
Title: Awareness and uptake of preconception care services in Tigray, Northern Ethiopia – a qualitative exploration of experiences, challenges, opportunities, and prospects

By: Gebremedhin Gebreegziabher Gebretsadik[1, 2*], Alemayehu Bayray Kahsay [2], Andargachew Kassa [4], Amanuel Gessessew [3], Zohra Lassi, [5, 6], Afework Mulugeta [2]

To: PLOS one

Dear Editor,

We, the authors of this manuscript, express our gratitude to the journal editors and reviewers for their thorough review, insightful comments, and valuable suggestions that significantly enhanced the quality of our work. We have rigorously revised the manuscript as per your questions and comments. We have included the point-by-point response in the table below, framed as reviewers' comments/questions and authors' responses. The detailed revisions and changes we made in the main document are prepared with track changes attached separately. We expect our revision will enable the manuscript to fit the journal better. Should you have any further inquiries, please feel free to contact us at your convenience.

<u>Point-by-point Responses to Editor's and Reviewers Comments</u>

**Editors and Reviewers' comments**	**Authors' Response**
**Editor comments**	
3. In the ethics statement in the Methods, you have specified that verbal consent was obtained. Please provide additional details regarding how this consent was documented and witnessed, and state whether this was approved by the IRB	Thank you for your valuable comment. We incorporated your comments in the document**.** We stated that after fully explaining the study's objectives, risks, and benefits, informed consent was obtained from all participants, and the verbal informed consent was also approved

	by the IRB of the college.
**Reviewer #1: PONE-D-24-41198_Review comments**	
**Title**	
1. I suggest removing "policy implications" from the title as the manuscript does not speak to policy clearly. The current title does not align well with the stated aim of the study to explore experiences, challenges and opportunities. I suggest something along these lines "*Awareness and uptake of preconception care services in Tigray, Northern Ethiopia – a qualitative exploration of experiences, challenges and opportunities*".	I really appreciate for your perspective and your suggestion is duly accepted.
**Methods**	
Study design:<br>1. The sentence "*Since 2020, Ethiopia has strategically integrated PCC into its health system, guided by newly developed guidelines (6, 7).*" will fit better in the introduction rather than in the study setting. If available data on the service delivery so far can be provided.	Thank you very much for your critical insight and your comments are well taken and incorporated in the document. The data about PCC services in Ethiopia was included in the introduction (Page 5, line 99-100).
2. "With" needs to be deleted in this statement. "*These zones include 591,481 women of reproductive age (23.5% of the population) and employ 1,731 healthcare providers, including 350 with Health extension workers (HEWs).*"	Thank you once again for your comment. We have addressed it and made the necessary corrections in the document.
3. The reference to a war in the statement "*However, the war damaged over 80% of health facilities, leading to a 40% decline in maternal and child health services, including institutional deliveries (26).*" needs to be clarified better. It may be useful to include a paragraph in the introduction describing the state of health services before and after the war including dates and possible reasons for the unrest. This will enable readers to understand the context better.	Thank you once again for your comment. We accepted your suggestion and incorporated it in the introduction (Page 4-5, line 86-93).

Recruitment of participants:<br>1. The statement "*We identified participants communicated with HEWs and women development group (WDGs) from HEW registers using purposive sampling, considering their pregnancy and risks*." is unclear; the sentence appears incomplete.	Thank you for your comment. We accepted your suggestion and modified the statement accordingly in the document line (Page 7, line 143-145)
2. How did the authors determine the "intention to become pregnant"? Was there any screening questionnaire? Where were potential participants identified? The health facility or within the community? Who identified the participants in either instance?	Thank you for your valuable comment.<br>Mothers intending to become pregnant were specifically identified using a single reproductive life plan (RLP) tool: "Would you like to become pregnant in the next six months?" This tool is considered more acceptable than other pregnancy intention checklists (1). Hence, participants were identified and contacted through HEWs and WDGs in the community using the RLP tool mentioned above.
3. Similarly, how did the authors identify the women who had a history of adverse pregnancy outcomes?	Thank you very much and we really appreciate your insight. High-risk mothers who participated in the IDI were selected and contacted through HEWs together with WDGs. In our context (Ethiopia), HEWs are expected to perform their role in the health post, community, and households by creating a linkage with WDGs.
4. What do ESOGA & EMA mean?	**Thank you again for your comments**<br>ESOGA is an abbreviation described as Ethiopian Obstetrics and Gynecology. Besides, EMA is named the Ethiopian Midwives Association. This is also incorporated in the manuscript (Page 7, line 152-153).
5. What strategies did the research team apply to "bracket" their prior experiences?	In explorative qualitative research, bracketing is a crucial process where researchers set aside their presuppositions, biases, assumptions, and prior experiences to objectively observe and describe a phenomenon. It requires researchers to remain honest, vigilant, and self-reflective about their perspectives, pre-existing beliefs, and evolving hypotheses throughout the study. To minimize biases related to prior experience of the research team(bracket)<br>We considered the following strategies

	1. During data collection, the team held daily debriefing sessions to address emerging issues and spent extended time with participants to gain deeper insights. We extended the research period to gain an in-depth understanding of the phenomena. We shared participant transcripts for verification and incorporated their feedback<br><br>2. Member checking , involving participants in the research process by seeking their feedback on findings and interpretation can help ensure that research do not impose their own meaning on to the data. for example in this study we conducted member checking with four participants, each representing a distinct group: HCPs, MNCH experts, and the two groups of mothers<br>3. While conducting participant interviews, we conducted independently coding for creating codes, and organizing these codes into categories and themes, the researcher team bracketed their prior experiences and knowledge to enhance the quality of the results<br>4. Training was conducted to all research team mainly data collection methods and the concept of reflexivity<br>5. We pretested the topic guide in a similar setting before starting data collection with revisions made based on feedback.<br>6. We used participants who have different perspective and IDIs, KIIs, and FGD s data collection methods the concept of triangulation of the study.<br><br>For detailed description, we have incorporated the trustworthiness of the qualitative study into the Methods and Materials section (page 9, lines 188–206).

Ethics approval and consent to participate: The statement "*Before data collection, we attached a one-page consent form to the questionnaire, explaining participants' autonomy*" implies that a survey was conducted whereas the study is described as qualitative. Was there a questionnaire survey in addition to the qualitative data collection? The methods need to be clarified appropriately.	Thank you very much for your concern. In this study we only conducted qualitative research, and the statement included was corrected and included in the document (page 11, line 230-234).
Results	
1. What is the justification for including teenagers in the study? Table 1 shows that there are two intending mothers between 15 and 19 years old.	Thank you very much for your insight. Based on the Ethiopian demographic health survey(3), the age category is presented starting from 15 years. However, unfortunately in this study the minimum age was 18 years (there were no mothers whose age less than 18 years). In general we did not include teenagers in the study.
2. Figure 1 – conceptual framework: please provide a brief description of the relationships between the concepts in the framework. A legend describing the directions of the different arrows included will also be helpful.	. Thank you once again for your valuable comments. We have made corrections to the themes and sub-themes, rearranged the figure to include arrows that effectively illustrate the relationships between concepts, and incorporated all modifications into the document accordingly (page 15, lines 260–268).
3. The triangulation of results can be improved. Some sections are fairly clear with opposing ideas around the same theme well presented. In other sections the presentation is not well aligned. It will also be useful to compare the opinions of health care providers and the different groups of women on the points rose to make the discussion more robust.	Thank you very much for sharing your valuable perspective. Based on your suggestion, we have made the necessary modifications to the document.
4. In some places the authors have written *St Mary* and in others *St Merry*. If this is a	Thank you once again for your suggestion. We have consistently updated and corrected the

reference to the same religious figure the spellings should be aligned.	term to "St. Mary" throughout the document.
**Discussion**	
1. The *“SMART Start model*” is mentioned in the results and again in the discussion. Is this a model described in the Ethiopian health care system? It would be useful to provide some explanation of the model and proffer possible suggestions on why it was mentioned as a potential strategy for delivery of PCC care in the study.	The Smart Start model, launched by PSI as part of Ethiopia's National Adolescent and Youth Health Strategy (2021–2025), is a girl-centered reproductive health initiative aimed at improving pregnancy outcomes and overall well-being. The program serves as an entry point for couples to address health risks and economic challenges by integrating contraceptive education, counseling, and financial preparedness. Additionally, it advocates for the use of contraceptives among adolescent girls and promotes awareness before pregnancy. The program is implemented by health extension workers at the kebele or tabia level, Ethiopia's smallest administrative units, following “kettena” or “kushet”.<br><br>(NATIONAL ADOLESCENTS AND YOUTH HEALTH STRATEGY (2021-2025))
2. It would be useful for the authors to proffer possible solutions to the challenges to PCC service provision and uptake identified in the study rather than only restate the issues already highlighted in the results.	Thank you very much for your grate insightful suggestion and based on your suggestion we did it.
3. Some of the themes in the results can be rephrased as strategies and opportunities for PCC services.	Thank you very much and we did it.
**Reviewer #2:REVIEW FOR PONE**	
It would have been better if there were line numbering for easy reference.	Thank you very much and we did it.
**Abstract**	
1. Restructure this sentence. It is too long. ‘Some women have an awareness of PCC services, mainly among high risk; the majority of healthcare providers, especially gynecologists, and doctors, have some knowledge of PCC and understand its importance and provide some components of	Thank you again for your comment. We modified in the document (page 2, line from 36 to 40)

PCC interventions in a fragmented way, primarily targeting high-risk women.	
**INTRODUCTION**	
2.Add more information about what is known about Experiences, Challenges, and Opportunities in Ethiopia	Thank you again for your suggestion<br>In Ethiopia, the concept of PCC especially package based services is a new concept and as far as our knowledge is concern, there is little evidence about Experiences, Challenges, and Opportunities of PCC services in Ethiopia.
**METHODS**	
3. How was trustworthiness ensured?	Thank you for your valuable comments.<br><br>We ensured the trustworthiness of the study through:<br>a. During data collection, the team held daily debriefing sessions throughout data collection time to address emerging issues and spent extended time with participants to gain deeper insights. We extended the research period to gain an in-depth understanding of the phenomena. We shared participant transcripts for verification and incorporated their feedback.<br><br>b. In this study we conducted member checking with four participants, each representing a distinct group: HCPs, MNCH experts, and the two groups of mothers<br>c. While conducting participant interviews, we conducted independently coding for creating codes, and organizing these codes into categories and themes, the researcher team bracketed their prior experiences and knowledge to enhance the quality of the results<br>d. Training was conducted to all research team mainly data collection methods and the concept of reflexivity<br>e. We pretested the topic guide in a similar setting before starting data collection with revisions made based on feedback.<br>f. We used participants who have different

	perspective<br>g. We used IDIs, KIIs, and FGD s as data collection methods to improve triangulation of the study<br><br>For detailed description we incorporate trustworthiness as separate section in the methods and materials section (page 9, lines 188–206).
4. Which qualitative method checklist was used for this study?	Thank you for your comment. We followed the PLOS one guidelines for reporting qualitative research using the consolidated criteria for reporting qualitative research (COREQ) checklist.
**RESULTS**	
5. This section needs a complete overhaul	Thank you very much for your valuable comments. In response to your comments, we have made significant revisions to the results section, including the subthemes, and we incorporated the updated results into the document.
6. Change results to findings	Thank you for your concern but the guidelines for PLOS one recommends using comment. results rather than findings
7. Table 1 is so confusing; it could be made better	Thank you for your feedback. Based on your suggestion, we have revised and separated the content into two tables, which have been incorporated into the document (page 12-14)
8. In Table 2, in the narratives, you mentioned 5 themes, but the table contains only 4 themes. In the same vein, you mentioned 29 subthemes, but the table contains 30 subthemes.	Thank you very much for your comment. We accepted your comment and rearranged and incorporated in the document (page 14-15)
9. The subthemes are simply too much, some of them are unnecessary and can be merged. You need to go back and redo your analysis to make the themes and especially the subthemes better. How can you have awareness, poor awareness, and knowledge as three different subthemes?	Thank you for your valuable suggestions. We made a significant modification and incorporated in the document (page 14-15).

10. What do you mean by 'Deliver in a fragmented' as a subtheme? Each subtheme should make sense	Thank you again for your comment. The phrase "Deliver in a fragmented manner" was included as a challenge in the subtheme. We categorized this to refer specifically to PCC services provided without a structured guideline, no responsible healthcare provider, no specific unit, meaning no package-based services are in place. Healthcare providers may deliver services inconsistently, relying solely on the information they have at hand or without any proper guidance. We have revised this to clarify it as "fragmented services."
11. Influence of elder persons and religious leaders can be merged with the Husband's opposition to PCC	Thank you once again for your valuable comments. We have revised and merged the significant subthemes, including those previously identified, and collectively named them "Social Influences."
12. I still fail to understand what this home-based preparation is all about. To me, it looks more like birth preparedness and has nothing to do with any component of PCC	The main aim of identifying home-based preparations for pregnancy is to assess whether community-based preparations align with preconception care interventions commonly practiced at the community level. The study found that mothers made preparations in areas such as economics, nutrition, alcohol intake, and psychological readiness, all of which are related to preconception care. These experiences can be used to educate women and raise awareness of preconception care components within the community. Preparation in these areas is essential to ensure a risk-free conception by addressing nutrition, alcohol, psychological issues, and other lifestyle risks ultimately improving pregnancy outcomes. While home-based preparation focuses on preconception care, birth preparedness occurs during pregnancy, mainly through antenatal care (ANC). Both are part of the maternal continuum of care and play a significant role in reducing adverse pregnancy outcomes.
**DISCUSSION**	
13. What do you mean here by 'Our uniquely explores the perspectives on PCC services in Tigray'	Thank you very much again for your comment and your suggestion is duly accepted. (page 39, line 797-798).
14. Write this in full when you first	Thank you for your comment

introduced it SMART	Smart Start Inc., established in September 1992 in America, and transformed Title X family planning guidelines by reducing barriers to contraceptive access. In 1993, regulatory changes allowed oral contraceptives to be provided without a full medical exam. Developed by the Family Planning Council of Southeastern Pennsylvania, SMART START enabled teens to delay certain medical services, fostering early access to care, improved contraceptive use, and reduced teen pregnancy rates(4). In Ethiopia, the Smart Start model, launched by PSI as part of Ethiopia's National Adolescent and Youth Health Strategy (2021–2025), is a girl-centered reproductive health initiative aimed at improving pregnancy outcomes and overall well-being. The program serves as an entry point for couples to address health risks and economic challenges by integrating contraceptive education, counseling, and financial preparedness.
15. There is no justification for any of your findings	Thank you very much for your comments. We have made significant revisions to the discussion section and incorporated them into the document.

1. Baldwin MK, Overcarsh P, Patel A, Zimmerman L, Edelman A. Pregnancy intention screening tools: a randomized trial to assess perceived helpfulness with communication about reproductive goals. Contraception and reproductive medicine. 2018;3:1-5.
2. Tufford L, Newman P. Bracketing in qualitative research. Qualitative social work. 2012;11(1):80-96.
3. Yigezu B. Ethiopian demographic and health survey. Central Statistical Agency. 2016.
4. Armstrong KA, Stover MA. SMART START: an option for adolescents to delay the pelvic examination and blood work in family planning clinics. Journal of Adolescent Health. 1994;15(5):389-95.
