# Supplementary material for: Preconception care services in Northern Ethiopia: A qualitative exploration of awareness, experiences, challenges, opportunities, and prospects
Source: PLoS One. 2025 Dec 3;20(12):e0337548. doi: 10.1371/journal.pone.0337548 (PMC12674573; doi:10.1371/journal.pone.0337548)
Supplement: Attachment — Submitted filename: PONE SECOND REVIEW.pdf [file pone.0337548.s008.pdf]

Thank you for allowing me to review this manuscript on Policy Implications of Preconception Care Services: Experiences, Challenges, and Opportunities in Tigray, Northern Ethiopia; an Exploratory Qualitative Study for the second time. Thanks to the authors for improving the paper by implementing the previous comments. Receive the additional comments below, which may assist in strengthening the paper further.

TITLE

I do not see any information regarding the uptake of PCC in the results; rather, I see practice. Kindly remove the uptake from the title since this concept is not covered.

ABSTRACT

Line 20…maternal continuum care. Rephrase this to either the maternal care continuum or the maternal continuum of care

Line 23. This aim does not reflect all the concepts in the title. Be consistent when stating your aim

Line 47: What do you mean by "perceived suggestions"

METHODS

Line 123: Be consistent when stating the aim of the study

RESULTS

Table 3: What do you mean by Perceived suggestions for PCC services? Why are the suggestions "perceived"

The result section is still very long and can be made concise by removing unnecessary quotes. For example, under the subtheme of 'use various communication strategies', lines 701 to 751, you used 9 quotes to support that subtheme, and I think that some of those quotes are repetitions

REFERENCES

Line 979, 982, etc. Some of your references are not well cited, for example, the Organisation WH, should be World Health Organization. Please correct them and other organisation references. Ensure to use the appropriate reference style of the journal.
