# Supplementary material for: Preconception care services in Northern Ethiopia: A qualitative exploration of awareness, experiences, challenges, opportunities, and prospects
Source: PLoS One. 2025 Dec 3;20(12):e0337548. doi: 10.1371/journal.pone.0337548 (PMC12674573; doi:10.1371/journal.pone.0337548)
Supplement: Attachment — Submitted filename: REVIEW FOR PONE.docx [file pone.0337548.s003.docx]

Thank you for allowing me to review this manuscript on Policy Implications of Preconception Care Services: Experiences, Challenges, and Opportunities in Tigray, Northern Ethiopia; an Exploratory Qualitative Study Find my comments which may assist in strengthening the paper. It would have been better if there were line numbering for easy reference.

ABSTRACT

1. Restructure this sentence. It is too long. ‘Some women have an awareness of PCC services, mainly among high risk; the majority of healthcare providers, especially gynecologists, and doctors, have some knowledge of PCC and understand its importance and provide some components of PCC interventions in a fragmented way, primarily targeting high-risk women.

INTRODUCTION

2. Add more information about what is known about Experiences, Challenges, and Opportunities in Ethiopia

METHODS

3. How was trustworthiness ensured?
4. Which qualitative method checklist was used for this study?

RESULTS

5. This section needs a complete overhaul
6. Change results to findings
7. Table 1 is so confusing; it could be made better
8. In Table 2, in the narratives, you mentioned 5 themes, but the table contains only 4 themes. In the same vein, you mentioned 29 subthemes, but the table contains 30 subthemes.
9. The subthemes are simply too much, some of them are unnecessary and can be merged.

You need to go back and redo your analysis to make the themes and especially the subthemes better. How can you have awareness, poor awareness, and knowledge as three different subthemes?

10. What do you mean by ‘Deliver in a fragmented’ as a subtheme? Each subtheme should make sense
11. Influence of elder persons and religious leaders can be merged with the Husband's opposition to PCC
12. I still fail to understand what this home-based preparation is all about. To me, it looks more like birth preparedness and has nothing to do with any component of PCC

DISCUSSION

13. What do you mean here by ‘Our uniquely explores the perspectives on PCC services in Tigray’
14. Write this in full when you first introduced it SMART
15. There is no justification for any of your findings
