# Supplementary material for: Preconception care services in Northern Ethiopia: A qualitative exploration of awareness, experiences, challenges, opportunities, and prospects
Source: PLoS One. 2025 Dec 3;20(12):e0337548. doi: 10.1371/journal.pone.0337548 (PMC12674573; doi:10.1371/journal.pone.0337548)
Supplement: Attachment — Submitted filename: PONE-D-24-41198_Review comments.docx [file pone.0337548.s004.docx]

**PONE-D-24-41198_Review comments**

**General comments**

There is a need for English editing of the paper to correct the grammatical errors and improve the clarity of the presentation.

**Title**

*Policy Implications of Preconception Care Services: Experiences, Challenges, and Opportunities in Tigray, Northern Ethiopia; an Exploratory Qualitative Study*

I suggest removing “policy implications” from the title as the manuscript does not speak to policy clearly. The current title does not align well with the stated aim of the study to explore experiences, challenges and opportunities. I suggest something along these lines “*Awareness and uptake of preconception care services in Tigray, Northern Ethiopia – a qualitative exploration of experiences, challenges and opportunities*”.

**Methods**

*Study design*

1. The sentence “*Since 2020, Ethiopia has strategically integrated PCC into its health system, guided by newly developed guidelines (6, 7).*” will fit better in the introduction rather than in the study setting. If available data on the service delivery so far can be provided.
2. “With” needs to be deleted in this statement. “*These zones include 591,481 women of reproductive age (23.5% of the population) and employ 1,731 healthcare providers, including 350 with Health extension workers (HEWs)*.”
3. The reference to a war in the statement “*However, the war damaged over 80% of health facilities, leading to a 40% decline in maternal and child health services, including institutional deliveries (26).*” needs to be clarified better. It may be useful to include a paragraph in the introduction describing the state of health services before and after the war including dates and possible reasons for the unrest. This will enable readers to understand the context better.

*Recruitment of participants*

1. The statement “*We identified participants communicated with HEWs and women development group (WDGs) from HEW registers using purposive sampling, considering their pregnancy and risks*.” is unclear; the sentence appears incomplete.
2. How did the authors determine the “intention to become pregnant”? Was there any screening questionnaire? Where were potential participants identified? The health facility or within the community? Who identified the participants in either instance?
3. Similarly, how did the authors identify the women who had a history of adverse pregnancy outcomes?
4. What do ESOGA & EMA mean?
5. What strategies did the research team apply to “bracket” their prior experiences?

*Ethics approval and consent to participate*

The statement “*Before data collection, we attached a one-page consent form to the questionnaire, explaining participants' autonomy*” implies that a survey was conducted whereas the study is described as qualitative. Was there a questionnaire survey in addition to the qualitative data collection? The methods need to be clarified appropriately.

**Results**

1. What is the justification for including teenagers in the study? Table 1 shows that there are two intending mothers between 15 and 19 years old.
2. Figure 1 – conceptual framework: please provide a brief description of the relationships between the concepts in the framework. A legend describing the directions of the different arrows included will also be helpful.
3. The triangulation of results can be improved. Some sections are fairly clear with opposing ideas around the same theme well presented. In other sections the presentation is not well aligned. It will also be useful to compare the opinions of health care providers and the different groups of women on the points raised to make the discussion more robust.
4. In some places the authors have written *St Mary* and in others *St Merry*. If this is a reference to the same religious figure the spellings should be aligned.

**Discussion**

The *“SMART Start model*” is mentioned in the results and again in the discussion. Is this a model described in the Ethiopian health care system? It would be useful to provide some explanation of the model and proffer possible suggestions on why it was mentioned as a potential strategy for delivery of PCC care in the study.

It would be useful for the authors to proffer possible solutions to the challenges to PCC service provision and uptake identified in the study rather than only restate the issues already highlighted in the results. Some of the themes in the results can be rephrased as strategies and opportunities for PCC services.
