# Supplementary material for: Preconception care services in Northern Ethiopia: A qualitative exploration of awareness, experiences, challenges, opportunities, and prospects
Source: PLoS One. 2025 Dec 3;20(12):e0337548. doi: 10.1371/journal.pone.0337548 (PMC12674573; doi:10.1371/journal.pone.0337548)
Supplement: Attachment — Submitted filename: Response to Reviewers.docx [file pone.0337548.s007.docx]

**Date: January 11/2024**

**Manuscript Number:** PONE-D-24-41198

**Title:** Awareness and uptake of preconception care services in Tigray, Northern Ethiopia – a qualitative exploration of experiences, challenges, opportunities, and prospects

By: Gebremedhin Gebreegziabher Gebretsadik^1, 2*^, Alemayehu Bayray Kahsay ^2^, Andargachew Kassa ^4^, Amanuel Gessessew ^3^, Zohra Lassi, ^5, 6^_,_ Afework Mulugeta ^2^

To: PLOS one

Dear Editor,

We, the authors of this manuscript, express our gratitude to the journal editors and reviewers for their thorough review, insightful comments, and valuable suggestions that significantly enhanced the quality of our work. We have rigorously revised the manuscript as per your questions and comments. We have included the point-by-point response in the table below, framed as reviewers' comments/questions and authors' responses. The detailed revisions and changes we made in the main document are prepared with track changes attached separately. We expect our revision will enable the manuscript to fit the journal better. Should you have any further inquiries, please feel free to contact us at your convenience.

**Point-by-point Responses to Editor’s and Reviewers Comments**

| **Editors and Reviewers' comments** | Authors’ Response |
| --- | --- |
| **Editor comments** |  |
| 3. In the ethics statement in the Methods, you have specified that verbal consent was obtained. Please provide additional details regarding how this consent was documented and witnessed, and state whether this was approved by the IRB | Thank you for your valuable comment. We incorporated your comments in the document**.** We stated that after fully explaining the study's objectives, risks, and benefits, informed consent was obtained from all participants, and the verbal informed consent was also approved by the IRB of the college. |
| **Reviewer #1: PONE-D-24-41198_Review comments** |  |
| **Title** |  |
| 1. I suggest removing “policy implications” from the title as the manuscript does not speak to policy clearly. The current title does not align well with the stated aim of the study to explore experiences, challenges and opportunities. I suggest something along these lines “*Awareness and uptake of preconception care services in Tigray, Northern Ethiopia – a qualitative exploration of experiences, challenges and opportunities*”. | I really appreciate for your perspective and your suggestion is duly accepted. |
| **Methods** |  |
| ***Study design:***  1. The sentence “*Since 2020, Ethiopia has strategically integrated PCC into its health system, guided by newly developed guidelines (6, 7).*” will fit better in the introduction rather than in the study setting. If available data on the service delivery so far can be provided. | Thank you very much for your critical insight and your comments are well taken and incorporated in the document. The data about PCC services in Ethiopia was included in the introduction (Page 5, line 99-100). |
| 2. “With” needs to be deleted in this statement. “*These zones include 591,481 women of reproductive age (23.5% of the population) and employ 1,731 healthcare providers, including 350 with Health extension workers (HEWs)*.” | Thank you once again for your comment. We have addressed it and made the necessary corrections in the document. |
| 3. The reference to a war in the statement “*However, the war damaged over 80% of health facilities, leading to a 40% decline in maternal and child health services, including institutional deliveries (26).*” needs to be clarified better. It may be useful to include a paragraph in the introduction describing the state of health services before and after the war including dates and possible reasons for the unrest. This will enable readers to understand the context better. | Thank you once again for your comment. We accepted your suggestion and incorporated it in the introduction (Page 4-5, line 86-93). |
| ***Recruitment of participants:***  1. The statement “*We identified participants communicated with HEWs and women development group (WDGs) from HEW registers using purposive sampling, considering their pregnancy and risks*.” is unclear; the sentence appears incomplete. | Thank you for your comment. We accepted your suggestion and modified the statement accordingly in the document line (Page 7, line 143-145) |
| 2. How did the authors determine the “intention to become pregnant”? Was there any screening questionnaire? Where were potential participants identified? The health facility or within the community? Who identified the participants in either instance? | Thank you for your valuable comment. Mothers intending to become pregnant were specifically identified using a single reproductive life plan (RLP) tool: "Would you like to become pregnant in the next six months?" This tool is considered more acceptable than other pregnancy intention checklists (1). Hence, participants were identified and contacted through HEWs and WDGs in the community using the RLP tool mentioned above. |
| 3. Similarly, how did the authors identify the women who had a history of adverse pregnancy outcomes? | Thank you very much and we really appreciate your insight. High-risk mothers who participated in the IDI were selected and contacted through HEWs together with WDGs. In our context (Ethiopia), HEWs are expected to perform their role in the health post, community, and households by creating a linkage with WDGs. |
| *4.* What do ESOGA & EMA mean? | **Thank you again for your comments**  ESOGA is an abbreviation described as Ethiopian Obstetrics and Gynecology. Besides, EMA is named the Ethiopian Midwives Association. This is also incorporated in the manuscript (Page 7, line 152-153). |
| 5. What strategies did the research team apply to “bracket” their prior experiences? | In explorative qualitative research, bracketing is a crucial process where researchers set aside their presuppositions, biases, assumptions, and prior experiences to objectively observe and describe a phenomenon. It requires researchers to remain honest, vigilant, and self-reflective about their perspectives, pre-existing beliefs, and evolving hypotheses throughout the study.  To minimize biases related to prior experience of the research team(bracket)  We considered the following strategies   1. During data collection, the team held daily debriefing sessions to address emerging issues and spent extended time with participants to gain deeper insights. We extended the research period to gain an in-depth understanding of the phenomena. We shared participant transcripts for verification and incorporated their feedback 2. Member checking , involving participants in the research process by seeking their feedback on findings and interpretation can help ensure that research do not impose their own meaning on to the data. for example in this study we conducted member checking with four participants, each representing a distinct group: HCPs, MNCH experts, and the two groups of mothers 3. While conducting participant interviews, we conducted independently coding for creating codes, and organizing these codes into categories and themes, the researcher team bracketed their prior experiences and knowledge to enhance the quality of the results 4. Training was conducted to all research team mainly data collection methods and the concept of reflexivity 5. We pretested the topic guide in a similar setting before starting data collection with revisions made based on feedback. 6. We used participants who have different perspective and IDIs, KIIs, and FGD s data collection methods the concept of triangulation of the study.   For detailed description, we have incorporated the trustworthiness of the qualitative study into the Methods and Materials section (page 9, lines 188–206). |
| ***Ethics approval and consent to participate:***  The statement “*Before data collection, we attached a one-page consent form to the questionnaire, explaining participants' autonomy*” implies that a survey was conducted whereas the study is described as qualitative. Was there a questionnaire survey in addition to the qualitative data collection? The methods need to be clarified appropriately. | Thank you very much for your concern. In this study we only conducted qualitative research, and the statement included was corrected and included in the document (page 11, line 230-234). |
| ***Results*** |  |
| 1. What is the justification for including teenagers in the study? Table 1 shows that there are two intending mothers between 15 and 19 years old. | Thank you very much for your insight. Based on the Ethiopian demographic health survey(3), the age category is presented starting from 15 years. However, unfortunately in this study the minimum age was 18 years (there were no mothers whose age less than 18 years). In general we did not include teenagers in the study. |
| 2. Figure 1 – conceptual framework: please provide a brief description of the relationships between the concepts in the framework. A legend describing the directions of the different arrows included will also be helpful. | .  Thank you once again for your valuable comments. We have made corrections to the themes and sub-themes, rearranged the figure to include arrows that effectively illustrate the relationships between concepts, and incorporated all modifications into the document accordingly (page 15, lines 260–268). |
| 3. The triangulation of results can be improved. Some sections are fairly clear with opposing ideas around the same theme well presented. In other sections the presentation is not well aligned. It will also be useful to compare the opinions of health care providers and the different groups of women on the points rose to make the discussion more robust. | Thank you very much for sharing your valuable perspective. Based on your suggestion, we have made the necessary modifications to the document. |
| 4. In some places the authors have written *St Mary* and in others *St Merry*. If this is a reference to the same religious figure the spellings should be aligned. | Thank you once again for your suggestion. We have consistently updated and corrected the term to "St. Mary" throughout the document. |
| **Discussion** |  |
| 1. The *“SMART Start model*” is mentioned in the results and again in the discussion. Is this a model described in the Ethiopian health care system? It would be useful to provide some explanation of the model and proffer possible suggestions on why it was mentioned as a potential strategy for delivery of PCC care in the study. | The Smart Start model, launched by PSI as part of Ethiopia's National Adolescent and Youth Health Strategy (2021–2025), is a girl-centered reproductive health initiative aimed at improving pregnancy outcomes and overall well-being. The program serves as an entry point for couples to address health risks and economic challenges by integrating contraceptive education, counseling, and financial preparedness. Additionally, it advocates for the use of contraceptives among adolescent girls and promotes awareness before pregnancy. The program is implemented by health extension workers at the kebele or tabia level, Ethiopia's smallest administrative units, following “kettena” or “kushet”.  ( NATIONAL ADOLESCENTS AND YOUTH HEALTH STRATEGY (2021-2025)) |
| 2. It would be useful for the authors to proffer possible solutions to the challenges to PCC service provision and uptake identified in the study rather than only restate the issues already highlighted in the results. | Thank you very much for your grate insightful suggestion and based on your suggestion we did it. |
| 3. Some of the themes in the results can be rephrased as strategies and opportunities for PCC services. | Thank you very much and we did it. |
| **Reviewer #2:REVIEW FOR PONE** |  |
| It would have been better if there were line numbering for easy reference. | Thank you very much and we did it. |
| **Abstract** |  |
| 1. Restructure this sentence. It is too long. ‘Some women have an awareness of PCC services, mainly among high risk; the majority of healthcare providers, especially gynecologists, and doctors, have some knowledge of PCC and understand its importance and provide some components of PCC interventions in a fragmented way, primarily targeting high-risk women. | Thank you again for your comment. We modified in the document (page 2, line from 36 to 40) |
| **INTRODUCTION** |  |
| 2.Add more information about what is known about Experiences, Challenges, and Opportunities in Ethiopia | Thank you again for your suggestion  In Ethiopia, the concept of PCC especially package based services is a new concept and as far as our knowledge is concern, there is little evidence about Experiences, Challenges, and Opportunities of PCC services in Ethiopia. |
| **METHODS** |  |
| 3. How was trustworthiness ensured? | Thank you for your valuable comments.  We ensured the trustworthiness of the study through:   a. During data collection, the team held daily debriefing sessions throughout data collection time to address emerging issues and spent extended time with participants to gain deeper insights. We extended the research period to gain an in-depth understanding of the phenomena. We shared participant transcripts for verification and incorporated their feedback.   b. In this study we conducted member checking with four participants, each representing a distinct group: HCPs, MNCH experts, and the two groups of mothers  c. While conducting participant interviews, we conducted independently coding for creating codes, and organizing these codes into categories and themes, the researcher team bracketed their prior experiences and knowledge to enhance the quality of the results  d. Training was conducted to all research team mainly data collection methods and the concept of reflexivity  e. We pretested the topic guide in a similar setting before starting data collection with revisions made based on feedback.  f. We used participants who have different perspective  g. We used IDIs, KIIs, and FGD s as data collection methods to improve triangulation of the study  For detailed description we incorporate trustworthiness as separate section in the methods and materials section (page 9, lines 188–206). |
| 4. Which qualitative method checklist was used for this study? | Thank you for your comment. We followed the PLOS one guidelines for reporting qualitative research using the consolidated criteria for reporting qualitative research (COREQ) checklist. |
| **RESULTS** |  |
| 5. This section needs a complete overhaul | Thank you very much for your valuable comments. In response to your comments, we have made significant revisions to the results section, including the subthemes, and we incorporated the updated results into the document. |
| 6. Change results to findings | Thank you for your concern but the guidelines for PLOS one recommends using comment. results rather than findings |
| 7. Table 1 is so confusing; it could be made better | Thank you for your feedback. Based on your suggestion, we have revised and separated the content into two tables, which have been incorporated into the document (page 12-14) |
| 8. In Table 2, in the narratives, you mentioned 5 themes, but the table contains only 4 themes. In the same vein, you mentioned 29 subthemes, but the table contains 30 subthemes. | Thank you very much for your comment. We accepted your comment and rearranged and incorporated in the document (page 14-15 ) |
| 9. The subthemes are simply too much, some of them are unnecessary and can be merged. You need to go back and redo your analysis to make the themes and especially the subthemes better. How can you have awareness, poor awareness, and knowledge as three different subthemes? | Thank you for your valuable suggestions. We made a significant modification and incorporated in the document (page 14-15 ). |
| 10. What do you mean by ‘Deliver in a fragmented’ as a subtheme? Each subtheme should make sense | Thank you again for your comment. The phrase “Deliver in a fragmented manner” was included as a challenge in the subtheme. We categorized this to refer specifically to PCC services provided without a structured guideline, no responsible healthcare provider, no specific unit, meaning no package-based services are in place. Healthcare providers may deliver services inconsistently, relying solely on the information they have at hand or without any proper guidance. We have revised this to clarify it as “fragmented services.” |
| 11. Influence of elder persons and religious leaders can be merged with the Husband's opposition to PCC | Thank you once again for your valuable comments. We have revised and merged the significant subthemes, including those previously identified, and collectively named them “Social Influences.” |
| 12. I still fail to understand what this home-based preparation is all about. To me, it looks more like birth preparedness and has nothing to do with any component of PCC | The main aim of identifying home-based preparations for pregnancy is to assess whether community-based preparations align with preconception care interventions commonly practiced at the community level. The study found that mothers made preparations in areas such as economics, nutrition, alcohol intake, and psychological readiness, all of which are related to preconception care. These experiences can be used to educate women and raise awareness of preconception care components within the community. Preparation in these areas is essential to ensure a risk-free conception by addressing nutrition, alcohol, psychological issues, and other lifestyle risks ultimately improving pregnancy outcomes. While home-based preparation focuses on preconception care, birth preparedness occurs during pregnancy, mainly through antenatal care (ANC). Both are part of the maternal continuum of care and play a significant role in reducing adverse pregnancy outcomes. |
| **DISCUSSION** |  |
| 13. What do you mean here by ‘Our uniquely explores the perspectives on PCC services in Tigray’ | Thank you very much again for your comment and your suggestion is duly accepted. (page 39, line 797-798). |
| 14. Write this in full when you first introduced it SMART | Thank you for your comment  Smart Start Inc., established in September 1992 in America, and transformed Title X family planning guidelines by reducing barriers to contraceptive access. In 1993, regulatory changes allowed oral contraceptives to be provided without a full medical exam. Developed by the Family Planning Council of Southeastern Pennsylvania, SMART START enabled teens to delay certain medical services, fostering early access to care, improved contraceptive use, and reduced teen pregnancy rates(4). In Ethiopia, the Smart Start model, launched by PSI as part of Ethiopia's National Adolescent and Youth Health Strategy (2021–2025), is a girl-centered reproductive health initiative aimed at improving pregnancy outcomes and overall well-being. The program serves as an entry point for couples to address health risks and economic challenges by integrating contraceptive education, counseling, and financial preparedness. |
| 15. There is no justification for any of your findings | Thank you very much for your comments. We have made significant revisions to the discussion section and incorporated them into the document. |

1.	Baldwin MK, Overcarsh P, Patel A, Zimmerman L, Edelman A. Pregnancy intention screening tools: a randomized trial to assess perceived helpfulness with communication about reproductive goals. Contraception and reproductive medicine. 2018;3:1-5.

2.	Tufford L, Newman P. Bracketing in qualitative research. Qualitative social work. 2012;11(1):80-96.

3.	Yigezu B. Ethiopian demographic and health survey. Central Statistical Agency. 2016.

4.	Armstrong KA, Stover MA. SMART START: an option for adolescents to delay the pelvic examination and blood work in family planning clinics. Journal of Adolescent Health. 1994;15(5):389-95.
