# Supplementary material for: Preconception care services in Northern Ethiopia: A qualitative exploration of awareness, experiences, challenges, opportunities, and prospects
Source: PLoS One. 2025 Dec 3;20(12):e0337548. doi: 10.1371/journal.pone.0337548 (PMC12674573; doi:10.1371/journal.pone.0337548)
Supplement: Attachment — Submitted filename: Response to ReviewersR.docx [file pone.0337548.s009.docx]

**Date April 29/2025**

**Manuscript Number:** PONE-D-24-41198

**Title:** Awareness and uptake of preconception care services in Tigray, Northern Ethiopia – a qualitative exploration of experiences, challenges, opportunities, and prospects

By: Gebremedhin Gebreegziabher Gebretsadik^1, 2*^, Alemayehu Bayray Kahsay ^2^, Andargachew Kassa ^4^, Amanuel Gessessew ^3^, Zohra Lassi, ^5, 6^_,_ Afework Mulugeta ^2^

To: PLOS one

Dear Editor,

We, the authors of this manuscript, express our gratitude to the journal editors and reviewers for their thorough review, insightful comments, and valuable suggestions that significantly enhanced the quality of our work. We have rigorously revised the manuscript as per your questions and comments. We have included the point-by-point response in the table below, framed as reviewers' comments/questions and authors' responses. The detailed revisions and changes we made in the main document are prepared with track changes attached separately. We expect our revision will enable the manuscript to fit the journal better. Should you have any further inquiries, please feel free to contact us at your convenience.

**Point-by-point Responses to Editor’s and Reviewers Comments**

| **Editors and Reviewers' comments** | Authors’ Response |
| --- | --- |
| **Reviewer #1:** |  |
| The authors should attach the COREQ checklist | Thank you very much. We have attached the COREQ checklist as a supplementary file(S5_COREQ checklist |
| What do you mean by "topic guide"? Line 190 | Thank you once again for your constructive comments. The topic guide has been modified as interview guide and incorporated into the manuscript on page 9, line 191. |
| Rephrase the last subtheme | Thank you again for your comment; we have made the suggested modifications accordingly. |
| The discussion section is a bit too long and can be shortened | Thank you for your suggestions. We have made efforts to streamline the discussion section and have incorporated the revised version into the manuscript. |
| The authors still did not provide any justifications for some of their findings | Thank you once again for your comments. We have included a justification of the findings and incorporated it into the revised manuscript. |
| **Reviewer #2:** |  |
| The authors have revised the manuscript adequately; however some of the issues raised in the previous review were addressed in the review table but not inserted into the manuscript. The authors need to check that these revisions are made on the manuscript as appropriate. | Thank you for your suggestion. We have included information on how women with pregnancy intentions were identified in the Methods section (revised manuscript, page 7, lines 140–142). We included how high risk women were identified for IDIs and included in the methods sections (revised manuscript, page 7, lines 145–148). Additionally, using purposive sampling, we identified mothers from HEW registers who had engaged with HEWs and WDGs, based on pregnancy status and risk factors. In the Ethiopian context, HEWs work across health posts, communities, and households in coordination with WDGs. Moreover, we described the Smart Start model in the Results section (page 33, lines 658–662). |
