# Supplementary material for: Preconception care services in Northern Ethiopia: A qualitative exploration of awareness, experiences, challenges, opportunities, and prospects
Source: PLoS One. 2025 Dec 3;20(12):e0337548. doi: 10.1371/journal.pone.0337548 (PMC12674573; doi:10.1371/journal.pone.0337548)
Supplement: Attachment — Submitted filename: Response_to_Reviewers_auresp_3.docx [file pone.0337548.s010.docx]

**Date November 8/2025**

**Manuscript Number:** PONE-D-24-41198R2

**Title:**

Preconception Care Services in Northern Ethiopia: A Qualitative Exploration of Awareness, Experiences, Challenges, Opportunities, and Prospects

By: Gebremedhin Gebreegziabher Gebretsadik^1, 2*^, Alemayehu Bayray Kahsay ^2^, Andargachew Kassa ^4^, Amanuel Gessessew ^3^, Zohra Lassi, ^5, 6^_,_ Afework Mulugeta ^2^

To: PLOS one

Dear Editor,

We, the authors of this manuscript, express our gratitude to the journal editors and reviewers for

their thorough review, insightful comments, and valuable suggestions that significantly enhanced the quality of our work. We have rigorously revised the manuscript as per your questions and comments. We have included the point-by-point response in the table below, framed as Editor/reviewers' comments and authors' responses. The detailed revisions and changes we made to the main document are prepared with track changes attached separately. We expect our revision will enable the manuscript to fit the journal better. Should you have any further questions, please feel free to contact us at your convenience.

**Point-by-point Responses to Editor’s and Reviewers Comments**

| **Editors comment on Journal Requirements** | Authors’ Response |
| --- | --- |
| If the reviewer comments include a recommendation to cite specific previously published works, please review and evaluate these publications to determine whether they are relevant and should be cited. There is no requirement to cite these works unless the editor has indicated otherwise. | Thank you very much for your suggestions. None of the reviewer comments included recommendations to cite specific previously published works. |
| Please review your reference list to ensure that it is complete and correct. If you have cited papers that have been retracted, please include the rationale for doing so in the manuscript text, or remove these references and replace them with relevant current references. Any changes to the reference list should be mentioned in the rebuttal letter that accompanies your revised manuscript. If you need to cite a retracted article, indicate the article’s retracted status in the References list and also include a citation and full reference for the retraction notice. | Thank you for your valuable suggestion. We have checked all reference which included in this manuscript and we did not cite retracted papers |
| **Reviewer’s comments** |  |
| TITLE |  |
| I do not see any information regarding the uptake of PCC in the results; rather, I see practice. Kindly remove the uptake from the title since this concept is not covered. | Thank you very much for your insightful comments and suggestions. We have corrected and modified the manuscript accordingly.  The revised title is: "***Preconception Care Services in Northern Ethiopia: A Qualitative Exploration of Awareness, Experiences, Challenges, Opportunities, and Prospects"*** |
| ABSTRACT |  |
| Line 20…maternal continuum care. Rephrase this to either the maternal care continuum or the maternal continuum of care | Thank you once again for your constructive comments. We have incorporated your feedback into the abstract section of the manuscript. |
| Line 23. This aim does not reflect all the concepts in the title. Be consistent when stating your aim | Thank you again for your comment; we have incorporated the suggested modifications into the manuscript. |
| Line 47: What do you mean by “perceived suggestions” | Thank you for your suggestions. We have duly incorporated your feedback and made corrections accordingly. |
| METHODS |  |
| Line 123: Be consistent when stating the aim of the study | We thank the reviewer for this valuable input, which has now been **incorporated** into the manuscript. |
| RESULTS |  |
| Table 3: What do you mean by Perceived suggestions for PCC services? Why are the suggestions “perceived” | We thank the reviewer for this valuable insight. We agree that in qualitative research, all participant-derived data are inherently 'perceived,' making the term redundant in this context. We have corrected this by changing the theme to "Suggestions for PCC Services" throughout the manuscript. |
| The result section is still very long and can be made concise by removing unnecessary quotes. For example, under the subtheme of ‘use various communication strategies, lines 701 to 751, you used 9 quotes to support that subtheme, and I think that some of those quotes are repetitions | Thank you very much. We reduced some quotes and ideas that seemed repetitive throughout the results. |
| REFERENCES |  |
| Line 979, 982, etc. Some of your references are not well cited, for example, the Organisation WH, should be World Health Organization. Please correct them and other organisation references. Ensure to use the appropriate reference style of the journal. | We are grateful for your constructive feedback. All cited references have been reviewed and corrected as needed, and the updated list is included in the manuscript. |
